# Coupling Climate Downscaling With Species Distribution Models to Identify Potential Climate Refugia for Giant Panda Forage Bamboos

**DOI:** 10.1002/ece3.74162

**Published:** 2026-08-07

**Authors:** Xiaotong Shang, Biao Yang, Qiang Dai, Han Pan, Lan Qiu, Yihong Wang, Chengcheng Zhang, Xuyu Yang, Xiaodong Gu, Zhisong Yang, Yang Liu, Li Zhang

**Affiliations:** ^1^ The State Key Laboratory of Biocontrol School of Ecology at Sun Yat‐Sen University Shenzhen China; ^2^ Key Laboratory of Biodiversity and Ecological Engineering, School of Life Sciences, Ministry of Education Beijing Normal University Beijing China; ^3^ School of Life Science (School of Giant Panda) China West Normal University Nanchong China; ^4^ Society of Entrepreneurs and Ecology (SEE) Foundation Beijing China; ^5^ Chengdu Institute of Biology Chinese Academy of Sciences Chengdu China; ^6^ University of Chinese Academy of Sciences Beijing China; ^7^ Sichuan Station of Wildlife Survey and Management Chengdu China; ^8^ Giant Panda National Park Administration Chengdu China; ^9^ Sichuan Academy of Giant Panda Chengdu China

**Keywords:** bamboo conservation, climate change, dispersal capacity, fine‐resolution, giant panda habitat, mountainous refuge

## Abstract

Climate change profoundly reshapes montane biodiversity patterns. A fine‐grained climatic resolution is indispensable to anticipate species‐specific habitat redistributions for effective conservation planning. Focusing on 20 giant panda forage bamboo (GPFB) species across 5 genera that constitute the bulk of the giant panda's ( *Ailuropoda melanoleuca* ) diet, we developed a “species‐distribution modelling based on climate‐delta downscaling” framework to project potential geographic shifts at a 30 m resolution across the 21st century under SSP1‐2.6, SSP2‐4.5, and SSP5‐8.5 scenarios. We found three primary results: (1) while the total climatically suitable area for GPFB expands under future scenarios, localized species richness within these habitats declines. (2) A mid‐elevation bamboo buffer zone (3000–4500 m) emerges as a critical thermal sanctuary where 7 out of 20 species expand under an unlimited dispersal assumption. However, incorporating a realistic biological dispersal constraint (10 m/year) severely truncates this elevational advantage, precipitating an overall habitat contraction for 11 species and reducing accessible refugia to merely 44%–65% of their theoretical potential. At localized scales, range shifts exhibit non‐monotonic trajectories and multidirectional movements rather than uniform upslope or northward migrations. (3) Integrating baseline and 12 future projections, we delineated stable macroclimate refugia governed by steep topographies, high hydrological availability, long‐term annual precipitation stability, and distinct seasonal environmental variations. 78.6% to 89.2% of the bamboo climate refuges overlap with the current (in 2025) forest distribution. Alarmingly, only 38.46%–44.11% of these crucial refugia fall within the current boundaries of the Giant Panda National Park, leaving extensive, contiguous refugia in the Liangshan mountain region largely unprotected. We recommend the establishment of a climate‐buffered conservation network that integrates extra‐park bamboo resources into unified spatial planning and deploys targeted connectivity corridors to mitigate dispersal bottlenecks. This 30 m workflow provides a high‐fidelity, transferable technical template for climate‐adaptation forecasting of threatened montane specialized diet species globally.

## Graphical abstract

This study addresses a critical gap in understanding how climate change will affect the foundation of giant panda habitat—its bamboo food resources—at a biologically meaningful scale. While previous assessments often relied on coarse‐resolution climate data, we present the first 30 m‐resolution projections for all 20 key bamboo species across the panda's range under multiple SSP scenarios (SSP1‐2.6 to SSP5‐8.5). Our novel “climate‐delta down‐scaling–species‐distribution modelling” framework reveals complex, fine‐grained dynamics that coarse‐scale models fail to capture.

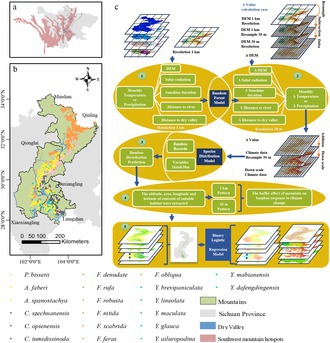

## 1 Introduction

Climate change poses a major challenge to global biodiversity conservation (Bellard et al. 2012; IPCC 2023). The current biodiversity conservation framework is based on existing patterns of biodiversity and the distribution of endangered species. However, at the landscape scale, the migration rates of most species cannot keep pace with the rapid processes of climate change (Ash et al. 2017; Lovejoy and Hannah 2019). Differences in migration rates can lead to profound changes in community composition and disrupt established ecological dynamics (Gillings et al. 2015; Hiddink et al. 2015; Urban et al. 2016). Integrating climate change into biodiversity conservation planning is crucial for achieving the 2030–2050 goals of the Kunming‐Montreal Global Biodiversity Framework (KM‐GBF, CBD 2022).

Climate, as a fundamental driver of biodiversity patterns, has a different impact on biodiversity in mountainous regions than in lowland regions (Körner 2004; Rahbek, Borregaard, Antonelli, et al. 2019; Rahbek, Borregaard, Colwell, et al. 2019; Perrigo et al. 2020). The uniqueness of mountains lies in their highly varied and complex topography, creating a mosaic of microclimates that provide rich niches for the survival of a variety of species. High species diversity is characteristic of the mountain (Körner 2004; Rahbek, Borregaard, Antonelli, et al. 2019; Rahbek, Borregaard, Colwell, et al. 2019; Perrigo et al. 2020). However, although Alexander von Humboldt began his interpretation of alpine biodiversity patterns as early as 1799, until now, there are still gaps in our understanding of mountain biodiversity patterns (Perrigo et al. 2020; Chang et al. 2023). Predicting the effects of climate change on the distribution of species in mountain areas is a cornerstone for developing future biodiversity conservation measures and a key factor in validating and extending theories on the formation of biodiversity patterns in mountain areas (Körner 2004; Urban et al. 2016).

Accurately predicting the distribution and biodiversity of mountain species is a complex challenge (Körner 2004; Bellard et al. 2012; Urban et al. 2016; Rahbek, Borregaard, Colwell, et al. 2019). Most climate data resolutions are at spatial scales of 30 arc‐seconds or higher. This coarse resolution obfuscates the diversity of mountain climate niches, which are fundamental to the formation and maintenance of mountain biodiversity patterns (Perrigo et al. 2020). The limitations of climate variable resolution hinder the predictive capabilities of species distribution models and our understanding of mountain biodiversity patterns (Randin et al. 2009; Maclean et al. 2019). While broad spatial trends can be identified with coarse resolution, it is less reliable for predicting the future distribution effects of climate change on localized species in complex mountainous regions, especially those species with limited dispersal capacity (Körner 2004; Randin et al. 2009). Utilizing climate data resolutions that match the terrain and activity scale of the studied species can yield more accurate species distribution predictions aligned with natural laws (Randin et al. 2009; Maclean et al. 2019). This can enhance the scientific basis and effectiveness of nature conservation planning decisions for mountain community management and conservation (Dax 2022; Xu et al. 2022).

The giant panda, one of China's most successfully protected species, relies on bamboo for 99% of its diet, which covers 90.4% of its total habitat area (National Forestry and Grassland Administration 2021). For over a decade, the impacts of climate change on the future population dynamics, habitat, and food resources of giant pandas have been continuously studied and monitored (Tuanmu et al. 2013; Li et al. 2015a, 2015b; Yan et al. 2017; Zhang et al. 2018; Wang et al. 2018). Previous modeling studies have presented a range of projections regarding how giant panda habitats and their primary food sources might respond to climate change. For instance, Tuanmu et al. (2013) analyzed and predicted the distribution of three bamboo species at a 30 arc‐second resolution in the Qinling Mountains, suggesting that the distribution range of these dominant species could face reductions in the 21st century, which might potentially lead to food shortages for the local panda populations. Similarly, Yan et al. (2017) modeled the bamboo foraging and habitat distribution of giant pandas in the Qionglai Mountain system, noting that while bamboo foraging areas were projected to expand to higher altitudes, the overall area was anticipated to decrease. Focusing on a different region, Zhang et al. (2018) analyzed the habitats of six bamboo species in the Daxiangling area. Their findings showed that under the RCP 2.6, 4.5, and 8.5 scenarios, the predicted area of suitable habitats was expected to decline by 45.6%, 57.4%, and 86.9%, respectively. Tang, Winkler, et al. (2018) emphasized the uncertainty of climate change data in predicting species distribution and highlighted the importance of considering uncertainty factors, climate baseline data, and research scales for future species conservation decisions. However, all the aforementioned studies were conducted at a resolution of 30 arc‐second (~1 km). This resolution obscures topographic and microclimatic details of giant panda habitats, potentially biasing projections of how bamboo in complex mountain regions will respond to climate change (Körner 2004; Randin et al. 2009).

**FIGURE 1:**
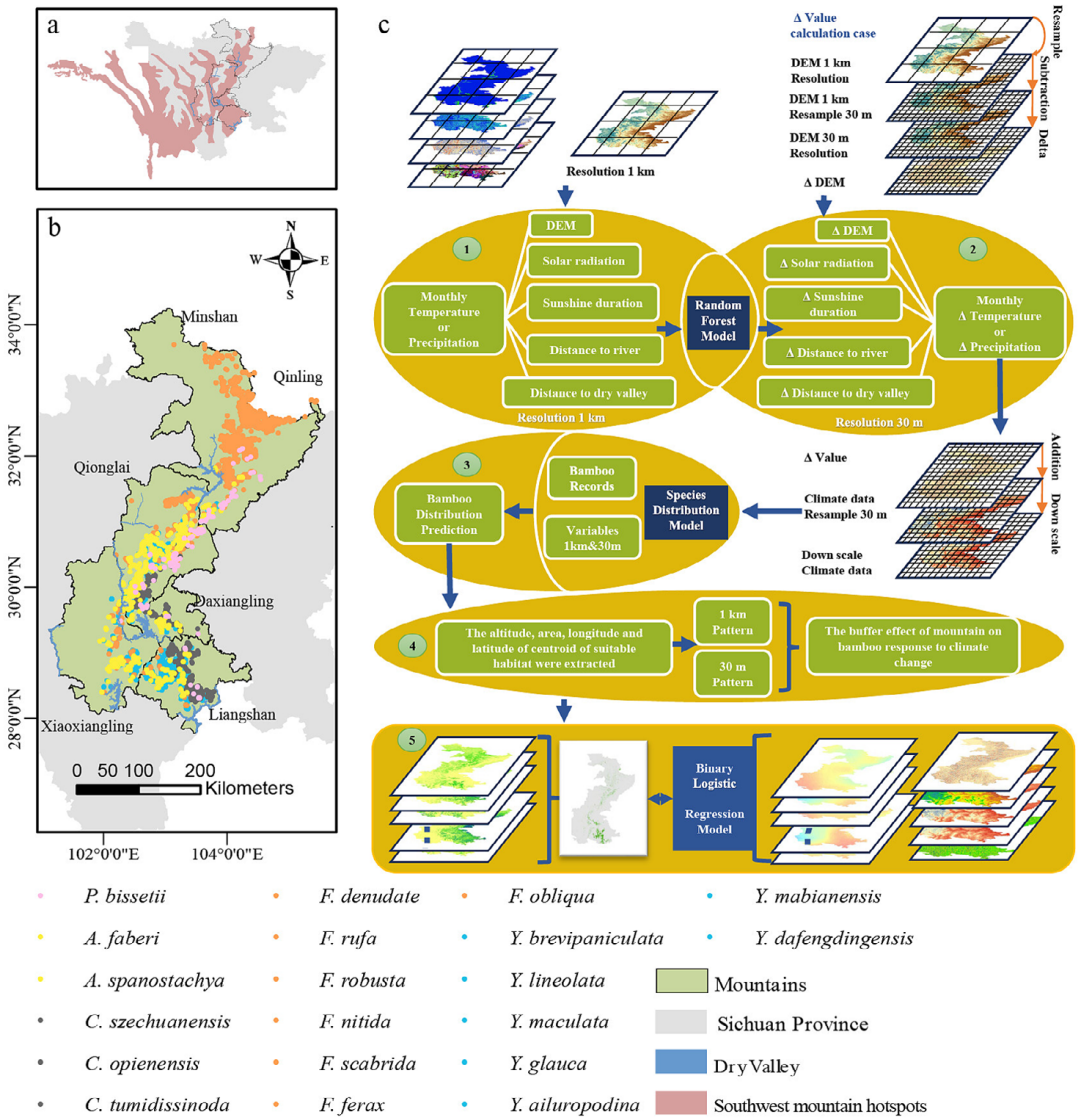
Study area, bamboo sites, and the workflow of the climate data delta‐downscaling and bamboo refuge identification methodological approach.

Moreover, in mountain ecosystems, specific small valleys retain “air pockets” formed by the inversion layer, providing a highly stable climate that serves as a refuge for species adapting to climate change (Tang, Matsui, et al. 2018; Rahbek, Borregaard, Colwell, et al. 2019). The habitat of giant pandas is characterized by mountains and deep valleys, including unique climatic zones such as the West China Rain Screen and Dry Valleys (Li et al. 2007; Yang et al. 2019; Fan et al. 2020) (Figure 1a,b). Additionally, the area has been proven to be a climate refuge for relict plants such as *Ginkgo biloba* and *Davidia* (Tang, Matsui, et al. 2018). The potential effects of mountain features on bamboo deserve further study.

Despite the proliferation of research on giant panda habitats, previous species distribution models (SDMs) operating at a coarse resolution of ~1 km (30 arc‐seconds) are insufficient for complex mountainous terrains. Such coarse scales effectively smooth out local topographic variations and critical microclimatic gradients, thereby obscuring the true environmental heterogeneity that sustains understory vegetation. To bridge this methodological gap, this study introduces a high‐resolution 30 m forecasting framework. This 30 m framework specifically adds the capacity to capture topographically sheltered “microclimate pockets” and high‐resolution climate suitability envelopes that are invisible at the 1 km scale, which is essential for species with limited dispersal capacities.

Here, we test three central hypotheses: (1) high‐resolution environmental data reveal more stable microrefugia for understory bamboo than coarse‐resolution models under climate change; (2) topography exerts a significant buffering effect, allowing mountain ecosystems to act as directional elevational buffers for specific bamboo genera; and (3) a constant dispersal limitation drastically reduces the functional connectivity and accessible area of future climate refugia. Furthermore, to prevent overinterpreting model‐specific projections, we explicitly evaluated model uncertainty across 3 Shared Socioeconomic Pathways (SSPs) and 2 General Circulation Models (GCMs). Specifically, our study is structured around three explicit objectives: (1) to map and summarize the potential future distribution and community feeding patterns of 20 giant panda forage bamboo (GPFB) species across 5 genera within giant panda habitats at both 1 km and 30 m resolutions. (2) To quantify the spatial buffering and microrefugia effects exerted by complex mountain topography at the community level. (3) To identify protection gaps by comparing the locations of simulated functional climate refugia against the current boundaries of the Giant Panda National Park and land cover layers. By addressing these objectives, this research aims to refine the scientific basis for adaptive conservation planning in mountain ecosystems.

## 2 Materials and Methods

### 2.1 Study Area and Bamboo Occurrence Sites

This study was carried out in the giant panda habitat in Sichuan Province, China (Figure 1a,b), utilizes understory bamboo occurrence data obtained during the 4th National Giant Panda Survey (2011–2014). From this dataset, we selected 20 dominant bamboo species with more than 30 occurrence records as our primary research subjects (Figure 1b, Table S1). To avoid artificially inflated model performance caused by overly broad background selections, the calibration region (background area) for our species distribution models (SDMs) was strictly constrained to the assemblage of panda‐presence counties in Sichuan Province. This spatial extent precisely corresponds to the macro‐ecological footprint of the 4th National Survey. Given the systematic nature of this nation‐level sampling campaign—which traversed all representative topographic matrices—and the widespread, overlapping understory distributions of montane bamboos, this designated region represents a shared, biologically accessible area for the studied species, effectively balancing pseudo‐absence selection with realistic colonization potential. The sampling design focused heavily on the key and potential habitats of giant pandas (Tang et al. 2015). The key survey areas were subdivided into 8 km^2^ vegetation survey zones, while the general survey areas were divided into larger 24 km^2^ zones. Each area was traversed by a vegetation survey transect line extending from lower to higher altitudes, covering all terrain types within the region. Along each transect line, numerous 20 × 20 m quadrats were established. Bamboo quadrats were nested within the vegetation quadrats, and three additional 1 × 1 m quadrats were placed within the bamboo quality patterns. For bamboo species with greater plant height, 2 × 2 m small quadrats were used (Tang et al. 2015). To address potential sampling bias, geographical spatial filtering was performed by keeping only one unique occurrence record per grid cell using cell numbers extracted via the raster package. To maintain rigorous taxonomic validation across decadal intervals, our research team carried out targeted field ground‐truthing from September to November 2022 across major giant panda core habitats in Sichuan. Ground surveys spanned key protected areas within the Minshan, Qionglai, Daxiangling, and Liangshan mountain ranges (including the Wanglang, Xiaohegou, Guanba, Laohegou, Xuebaoding, Xiaozhaizigou, Daxiangling, Shenguozhuang, and Meigu nature reserves, as well as the subalpine bamboo‐distributed areas in Kangding City and Luding County). The field‐verified taxonomic assignments and spatial persistence profiles highly converged with the baseline data from the 4th National Survey, ensuring high fidelity in species identification for our predictive modeling.

### 2.2 Climate Data Selection and Downscale

In this study, climate data from two General Circulation Models (GCMs) in the WorldClim database were used to build climate downscaling models and predict the distribution of bamboo species (Yang et al. 2021). These two specific GCMs were selected based on their proven individualistic accuracy in the East Asian monsoon region. The first model, GISS‐E2‐1‐G (NASA Goddard Institute for Space Studies, G model), excelled in simulating maximum and minimum temperature variations and their temporal evolution within China from 1961 to 2014 (Stark and Fridley 2022; Wenqiang et al. 2022). The second model, MRI‐ESM2‐0 (Meteorological Research Institute, Japan Meteorological Agency, M model), demonstrated proficiency in predicting humid areas in China (Li et al. 2021). By coupling a thermally optimized model with a hydrologically proficient model, this dual‐GCM framework captures the primary environmental factors limiting bamboo niche configurations. Our analysis was conducted under the framework of three distinct Shared Socioeconomic Pathways (SSPs): SSP1‐2.6 (limiting warming to 2°C, green‐growth paradigm, radiative forcing 2.6 W/m^2^), SSP2‐4.5 (limiting warming to 3°C, middle‐of‐the‐road development, radiative forcing 4.5 W/m^2^), and SSP5‐8.5 (limiting warming to 4°C, high energy demand with extensive fossil fuel use, radiative forcing 8.5 W/m^2^) (IPCC 2023). These pathways were defined by Rogelj (2018) and can be accessed at WorldClim (https://worldclim.org/).

The structural workflow of our climate data statistical delta‐downscaling and validation approach is illustrated in Figure 1c. To account for their distinct spatial boundaries and physical behaviors in rugged terrain, temperature and precipitation datasets were treated through decoupled downscaling pathways. The step‐by‐step reproducible protocol is executed as follows:

#### 2.2.1 Step 1: Topographic Delta and Predictor Generation

We computed the baseline elevation delta (ΔDEM) by subtracting the resampled 30 m Digital Elevation Model (DEM) from the original 1 km resolution DEM (Figure 1c, Delta calculation case). Concurrently, microclimatic raster predictors—including seasonal/monthly solar radiation, sunshine duration, distance to rivers, and distance to dry valleys—were generated at both 1 km and 30 m resolutions using SAGA‐GIS 8.2.1 (Conrad et al. 2015).

#### 2.2.2 Step 2: Microclimate Random Forest Regression Model

Using the baseline 1 km layers, month‐specific ensemble Random Forest (RF) regression models (ntree = 500) were constructed (Figure 1c, Circle 1). These models established localized statistical relationships between the geographic topoclimatic predictors and baseline monthly climate grids to mathematically capture fine‐scale environmental forcing:

$${\text{Climate}}_{1\mathrm{km}}^{\left(i\right)}=f_{\mathrm{RF}}^{\left(i\right)}\left({\mathrm{DEM}}_{1\mathrm{km}},{\text{Solar}}_{1\mathrm{km}}^{\left(i\right)},{\text{Sunshine}}_{1\mathrm{km}}^{\left(i\right)},{\text{DryValley}}_{1\mathrm{km}}\right)$$

where i denotes the corresponding month of the annual cycle ($i\in \left[1,12\right]$), and $f_{\mathrm{RF}}^{\left(i\right)}$ represents the trained month‐specific RF regression operator.

#### 2.2.3 Step 3: Month‐Specific Residual Delta Downscaling

To extract macroclimate anomalies while respecting the high‐resolution topographic gradients, the high‐resolution 30 m environmental predictors (Figure 1c, Resolution 30 m) were first transformed into delta surfaces relative to the 1 km baseline: ΔDEM, ΔSolar, ΔSunshine, and ΔDryValley. These delta predictors were then fed into the month‐specific trained Random Forest (RF) models (1 km resolution) to directly generate a localized correction term, rather than a residual between coarse and fine scales.

For monthly temperature updates, the final downscaled grid was obtained by adding this correction term to the coarsely resampled GCM baseline:

$$\Delta T_{\text{residual},30m}^{\left(i\right)}=f_{\mathrm{RF},\mathrm{tm}}^{\left(i\right)}\left(\Delta {\mathrm{DEM}}_{30m},{\text{ΔSolar}}_{30m}^{\left(i\right)},{\text{ΔSunshine}}_{30m}^{\left(i\right)},{\text{ΔDryValley}}_{30m}\right)$$

$$T_{\text{downscaled},30m}^{\left(i\right)}=T_{\text{baseline},30m}^{\left(i\right)}+\Delta T_{\text{residual},30m}^{\left(i\right)}$$

For monthly precipitation updates, a parallel approach was applied using only the topographic and dry‐valley delta predictors. In contrast to the original residual formulation, no subtraction of the 1 km resampled precipitation was performed, and no zero‐bounded truncation was applied to the final output:

$$\Delta P_{\text{residual},30m}^{\left(i\right)}=f_{\mathrm{RF},\mathrm{pr}}^{\left(i\right)}\left(\Delta {\mathrm{DEM}}_{30m},{\text{ΔDryValley}}_{30m}\right)$$

$$P_{\text{downscaled},30m}^{\left(i\right)}=P_{\text{baseline},30m}^{\left(i\right)}+\Delta P_{\text{residual},30m}^{\left(i\right)}$$

Here $T_{\text{baseline},30m}^{\left(i\right)}$ and $P_{\text{baseline},30m}^{\left(i\right)}$ represent the coarse future GCM projections directly resampled to a 30 m spatial grid (labeled as tm and pr in the workflow).

#### 2.2.4 Step 4: Bias Correction and Empirical Validation

We extracted the downscaled predicted values from the corresponding pixels of 42 independent meteorological stations distributed across the study area (Figure 1b) and compared them with the observed records. Concurrently, accuracy assessment was performed based on calibrated grids (1970–2000, 2041–2060, and 2081–2100) sourced from the National Tibetan Plateau Data Center. The accuracy of the downscaled 30 m data was rigorously validated using Mean Absolute Error (MAE), Root Mean Square Error (RMSE), and Pearson Correlation Coefficients (PCC) (Figure 1c; Tables S44–S49).

We subsequently computed the bio1–bio19 bioclimatic variables in R 4.2.1 (R Core Team 2022) following established formulations (O'Donnell and Ignazio 2012). In conjunction with regional seasonal characteristics (Li et al. 2007; Shibo et al. 2018), seasons were delineated as follows: spring (Feb 6–Mar 31), summer (Apr 1–Sep 5), autumn (Sep 6–Oct 10), and winter (Oct 11–Feb 5 of the following year). Seasonal solar radiation arrays were mapped via SAGA‐GIS based on the corresponding 1 km and 30 m DEM substrates. The technical precision of the downscaled 30 m grids was evaluated using a 10‐fold spatial cross‐validation within the Random Forest model. Furthermore, empirical validation was conducted by comparing monthly, seasonal, and derived bioclimatic variables against ground‐truth records from 42 independent meteorological stations (Figure 1b). The downscaled 30 m outputs significantly minimized systematic errors compared to the original 1 km baseline fields (Tables S43–S48), validating the biological and microclimatic reliability of our high‐resolution layers for subsequent species distribution modeling.

### 2.3 SDM Variable Selection, Modeling, and Prediction

To fully capture the ecological requirements of the understory bamboo, an initial pool of 59 environmental candidate variables was assembled. This included 19 baseline bioclimatic factors (Bio1–Bio19), 16 seasonal climate variables (maximum, minimum, and mean temperatures, alongside total precipitation for each season), 20 seasonal solar radiation‐related variables, slope, soil type, and two variables representing the distance to hydrological features (dry valleys and other rivers).

To eliminate multi‐collinearity and prevent model overfitting, a stringent variable selection procedure was executed via Pearson correlation analysis. For any pair of variables with a high correlation coefficient (|*r*| > 0.75), we strictly retained only the predictor that possessed the most direct and well‐established physiological relevance to bamboo growth and shooting dynamics (Table 1). In ecological distribution modeling, a threshold of |*r*| > 0.70–0.75 is widely recognized as a highly robust alternative to inflation diagnostics. Critically, to ensure strict direct comparability between the spatial predictions across different resolutions, an identical set of these 13 selected predictors was implemented for both the 1 km and 30 m scales.

**TABLE 1:** Selection and description of environmental variables in the MaxEnt model of GPFB.

Variables	Description	Unit
Bio4	Temperature Seasonality	/
Bio7	Temperature Annual Range	°C
Bio12	Annual Precipitation	Mm
Bio15	Precipitation Seasonality	Mm
Spring Precipitation	Add up the rainfall in February and March	Mm
Summer Mean Temperature	Average monthly temperature from April to August	°C
The amount of direct sunlight in spring	Direct sunlight from February 6 to March 31	kWh/m^2^
The amount of direct sunlight in summer	Direct sunlight from April 1 to September 5	kWh/m^2^
Summer Sunshine Duration	First calculate the duration of sunshine on April 1, June 22, June 23, and September 5, Summer Sunshine Duration = [(4.1 + 6.22 + 6.23 + 9.5)/4]*158	Hours
Distance to Dry Valleys	Log‐transformed distance (capped at 1000 m)	/
Distance to other rivers		/
Agrotype	classified variable	/
Slope	Sin(n), 0° ≤ *n* ≤ 90°	/

Species occurrence data and environmental layers were integrated into a Species With Data (SWD) object using the prepareSWD function in the R package SDMtune (v1.3.1; Vignali et al. 2020). To address potential sampling bias, geographical spatial filtering was performed by keeping only one unique occurrence record per grid cell using cell numbers extracted via the raster package. A total of 10,000 random pseudo‐absence/background points were generated across the study area, excluding the filtered presence grids. To minimize spatial autocorrelation and robustly evaluate model performance, a 4‐fold spatial block cross‐validation was implemented using the get.checkerboard2 function from the ENMeval package (v2.0.4; Muscarella et al. 2014).

Before final modeling, we conducted preliminary model experiments at two scales—the nature reserve scale (Wolong Nature Reserve) and the county scale—and performed an automated data‐driven optimization (tuning) procedure. We performed a grid search over regularization multipliers (ranging from 0.5 to 4.0 with an interval of 0.5) and feature classes (L, LQ, H, LQH, LQHP, LQPHT) using SDMtune. The optimal configuration was selected based on the lowest Akaike Information Criterion corrected (ΔAICc < 2) and low omission rates. For our bamboo species, the tuning confirmed that a Regularization Multiplier of 1.0 paired with the Linear‐Quadratic‐Product‐Hinge‐Threshold (LQPHT) feature combination yielded the best balance between predictive power and complexity. The final Maximum Entropy (MaxEnt v3.4.4) models were then fitted with 500 maximum iterations.

Model predictive performance was comprehensively evaluated using the Area Under the Receiver Operating Characteristic Curve (AUC), True Skill Statistic (TSS), 10% training omission rate, and the Continuous Boyce Index (CBI). Model prediction uncertainty was assessed via the standard deviation (SD) across the 4‐fold spatial replicates (Table S2). The optimized models were applied to project continuous Habitat Suitability Index (HSI) values under current and future climate scenarios at both scales. Finally, continuous maps were transformed into binary presence‐absence distributions using the Maximum Training Sensitivity plus Specificity (Max SSS) thresholding method via the plotPA function in SDMtune. All spatial analyses and graphics were streamlined in R environment v4.2.1 (R Core Team 2022) using packages including raster, pROC, and ggplot2.

All spatial analyses and macro‐ecological forecasting were streamlined in the R environment v4.2.1 (R Core Team 2022). The key algorithmic setting and versions applied include SDMtune (v1.3.1) for parameter tuning, ENMeval (v2.0.4) for spatial block cross‐validation, and megaSDM (v1.0; Shipley et al. 2022) for multi‐time‐step cumulative dispersal simulation and community richness mapping.

### 2.4 Acquisition of Bamboo Feeding Community Data of Giant Panda

The suitable habitats of 20 species of bamboo with and without diffusion restriction at 1 km resolution and 30 m resolution were superimposed. The diversity and distribution changes of GPFB communities under different resolutions and diffusion conditions were obtained for further analysis. Habitats suitable for four or more species of bamboo at the same time were considered areas of high diversity of bamboo.

### 2.5 Analysis of Area Changes and Altitude‐Dependent Dispersal Patterns

We extracted predicted distribution area results for all species, using the current predicted distribution area (*A*_cur_) as a baseline. The percentage change in area for each bamboo species by mid‐century and by the end of the century was calculated as PCT_area_. *A*_t_ represents the suitable habitat or distribution area for the corresponding future period, enabling the calculation of the percentage increase or decrease in area in the future.

$${\mathrm{PCT}}_{\text{area}}=\frac{A_{t}-A_{\mathrm{cur}}}{A_{\mathrm{cur}}}\times 100\%$$

Compute centroids for Suitable Habitat (1970–2000), Future Suitable Habitat (2041–2060, 2081–2100), and Future Suitable Habitat under Dispersal Constraints (10 m/year, 2041–2060, 2081–2100) using binary raster layers. Assess the angles formed by lines connecting mid‐century and end‐of‐century centroids to the current centroid and true north. Wilcoxon matched‐pairs signed rank test was performed on the matched data.

Due to the paucity of empirical field data regarding long‐term bamboo migration rates under contemporary climate change, we utilized a dispersal limit of 10 m/year as an optimistic biological benchmark, derived from average rhizome elongation and shooting frequencies. To rigorously implement these biological dispersal limitations and track transitory range dynamics across consecutive periods, we utilized the advanced cellular‐filtering framework within the R package megaSDM (Shipley et al. 2022). Range shifts were simulated recursively across multiple time steps from the current baseline to the mid‐century (2050) and late‐century (2100) horizons, meaning that dispersal constraints at later steps were cumulative and fully conditioned upon the realized, restricted distributions of the preceding time step. We emphasize that this parameter is not intended to model exact species‐specific trajectories, given that individualistic range shifts on decadal timescales can be highly heterogeneous and nonlinear (Freeman et al. 2026). Rather, this threshold functions as a baseline constraint to evaluate the severe consequences of migration lags—specifically, whether imposing biological boundaries reveals critical trends of suitable habitat contraction or elevated extinction risk compared to unconstrained niche tracking models.

Based on the elevation characteristics of the study area and bamboo species' altitude data from the Giant Panda's fourth survey, categorize the entire study area into four elevation intervals: “< 1500 m,” “1500–3000 m,” “3001–4500 m,” and “> 4500 m.” For each species, calculate the area and proportion of “Future Suitable Habitat under Dispersal Constraints” and “Future Suitable Habitat” within these elevation intervals. Analyze area and proportion changes across these elevation bands. This approach elucidates altitude‐dependent dispersal patterns of bamboo in localized regions, influenced by mountainous terrain.

### 2.6 Bamboo Climate Refugia Identification and Environmental Determinants Analysis

To delineate stable habitats under climate change, we superimposed 13 binary species distribution model (SDM) layers of bamboo communities, encompassing the current distribution along with six future climate scenarios for both the mid‐century (2041–2060) and late‐century (2081–2100) horizons. Grid cells predicted as suitable across all 13 integrated layers were strictly defined as macroclimate refugia for giant panda forage bamboo.

To rigorously disentangle the environmental determinants driving refugia formation, we analyzed a comprehensive suite of predictors, including topographic features (elevation and slope sine), micro‐climate and radiation regimes (summer sunshine duration and direct solar radiation), hydrological accessibility (log‐transformed, 1000 m‐capped distance to rivers and to dry valleys), and long‐term climatic stability metrics (calculated as the standard deviation (SD) of BIOCLIM variables, bio1–bio19, across all 13 scenarios).

Given the immense volume of the 30‐m high‐resolution raster data, a stratified balanced random sampling protocol was deployed to circumvent computational memory saturation and mitigate sample size imbalance. Specifically, we leveraged the pixel‐dimensional index positioning technique via the terra package to identify the spatial coordinates of all refugia (Presence, R_1_) and non‐refugia (Absence, R_0_) cells. A total of 10,000 pixels (5000 for R_1_ and 5000 for R_0_, respectively) were randomly extracted to construct a balanced modeling dataset. All continuous predictors were subsequently standardized using *Z*‐score transformation to eliminate dimensional scales and permit direct comparison of effect sizes (β).

To safeguard against the inflation of type I errors caused by severe multi‐collinearity among the 19 bioclimatic stability metrics, a rigorous Variance Inflation Factor (VIF) diagnostic was performed using a global generalized linear model (GLM). Variables exhibiting a VIF higher than 5 were stepwise excluded, retaining a parsimonious subset of 10 noncollinear core drivers (elevation, slope sine, soil type, summer sunshine duration, summer direct radiation, distance to rivers, distance to dry valleys, sd_bio4, sd_bio12, and sd_bio15) for formal regression.

Finally, a binary logistic regression model embedded within a 10‐fold cross‐validation framework (implemented via the caret package) was executed. This iterative cross‐validation mechanism randomly partitioned the sample into ten subsets, utilizing nine for model training and one for independent validation per cycle, to evaluate model generalizability and stability. The relative importance and effect sizes of each environmental determinant on bamboo refugia occurrence were quantified using standardized regression coefficients (β), odds ratios (OR), and their corresponding 95% confidence intervals, and visualized via a standardized forest plot.

### 2.7 Land Cover Filtering and Composition Analysis

To evaluate the compatibility of the identified climate refugia with conservation planning, the multi‐scenario refugia boundaries were overlayed with the 2025 China Land Cover Dataset (CLCD) (Yang and Xin Huang 2026). Four refugia scenarios—differentiated by spatial resolutions (1 km vs. 30 m) and species dispersal constraints (10 m/yr. vs. unlimited)—were utilized as spatial masks. To eliminate geometric incompatibility during overlay analysis, the coarse‐resolution (1 km) refugia matrices were downscaled to the 30 m grid template via spatial resampling (nearest neighbor). The landscape matrix within the whole study area and the four isolated refugia zones was quantified across the 9 categorical CLCD classes. The total physical area (km^2^) and the relative proportion (%) for each land‐use class were calculated.

## 3 Results

### 3.1 Species Diversity and Distribution of GPFB Communities

**FIGURE 2:**
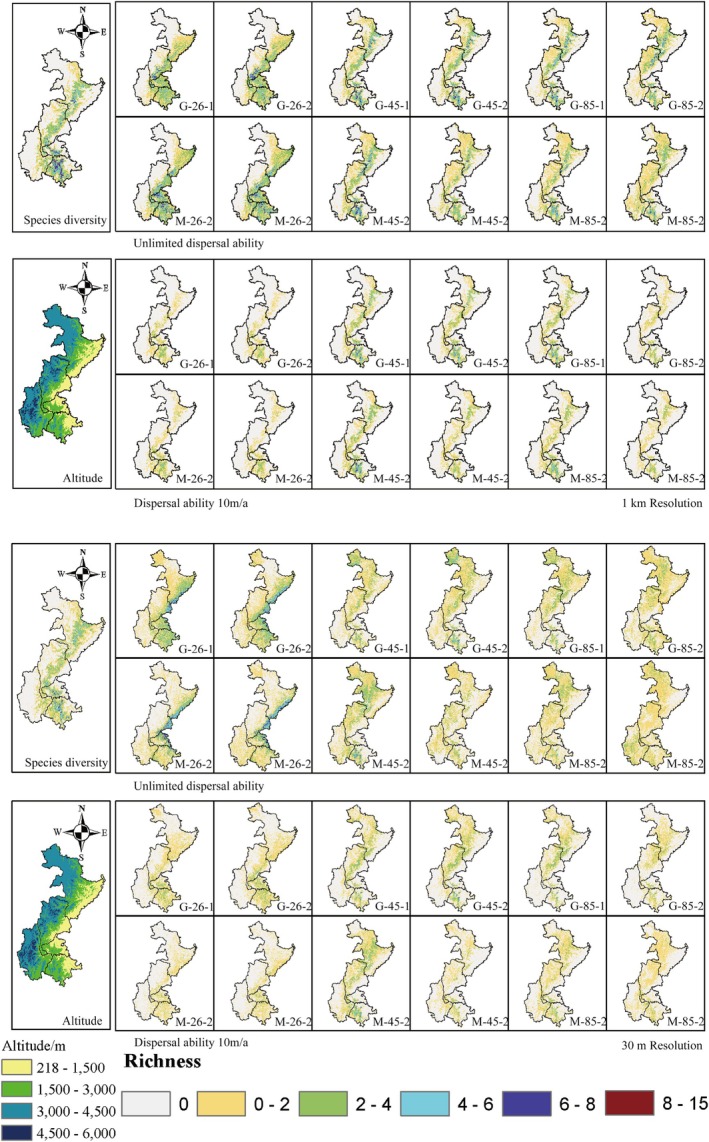
Responses of biodiversity and distribution of GPFB communities to climate change. “G” represents GISS‐E2‐1‐G, “M” represents MRI‐ESM2‐0, and “25,” “45,” and “85” correspond to the Shared Socioeconomic Pathways (SSPs). “1” denotes the mid‐century (2050), and “2” signifies the end of the century (2100).

The area of the current bamboo community accounts for 39.63% (52,149 km^2^) to 40.69% (52,296.87 km^2^) of the total area of the giant panda distribution counties in Sichuan Province, China, of which high bamboo diversity area accounts for 9.95% (13,128.39 km^2^) to 12.75% (16,342 km^2^) (Figure 2). Under unconstrained dispersal scenarios, the area predicted at a 30 m resolution was systematically larger than at 1 km, whereas this trend became scenario‐dependent under dispersal constraints (Figure 2). However, once the bamboo species' dispersal ability is taken into account, the area of the bamboo community shrinks by approximately 1/3 to 1/2, and the area of the highly diverse zone even drops to less than half of its original size, even falling below 0.2% in some scenarios. Regardless of whether there are limitations on the diffusion ability, the area changes brought about by different climate scenarios (G−/M‐ series) in the future will be significant, causing the area of bamboo communities to fluctuate between 21% and 66%, and the area of high‐diversity zones to range from 0.2% to 16% (Figure 2). Detailed species‐level results are provided in Figures S3–S22 and Tables S3–S42, area data of each bamboo's habitat with or without limitation is in Tables S51–56.

### 3.2 Buffer Effect of Mountain Elevation on Bamboo Response to Climate Change

**FIGURE 3:**
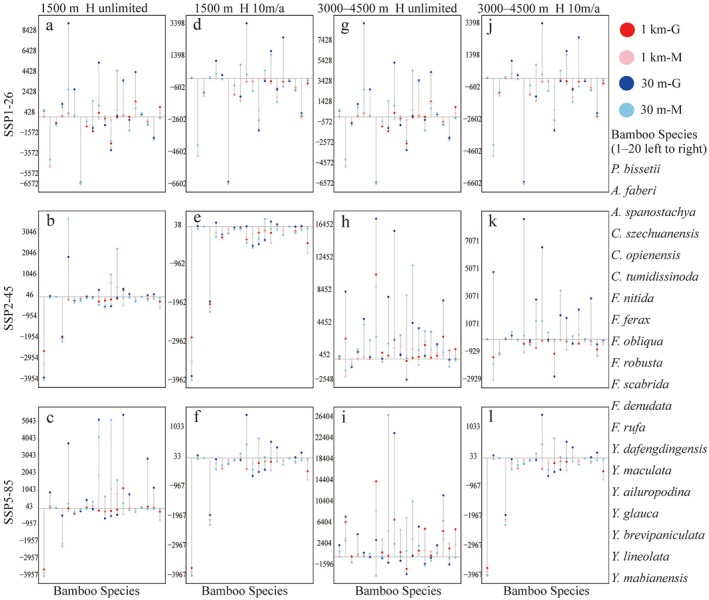
An overview of the alterations in suitable habitat areas for 20 bamboo species across low‐altitude regions below 1500 m and high‐altitude regions (3000–4500 m). The red dots correspond to results projected by the G model at a 1 km resolution. The pink dots signify predictions made by the M model at a 1 km resolution. The deep blue dots indicate forecasts generated by the G model at a finer 30 m resolution, and the light blue dots depict predictions from the M model at a 30 m resolution. Moving from left to right on the horizontal axis, species 1 through 20 can be observed, respectively.

Based on the dominant climate types in the study region, we selected the M model plus SSP2‐4.5 scenario—the combination closest to the current development trajectory—as an example. Details regarding different climate models and time series across the altitudinal range of 3000–4500 m are presented in Tables S51–S56. At both 1 km and 30 m resolutions, the results consistently show that high‐elevation zones at 3000–4500 m significantly buffer climate change impacts, with roughly one‐third (7 of 20) of bamboo species maintaining continuous expansion of suitable habitat within this belt (Figure 3). Once dispersal limitation is introduced, however, all species shift to persistent decline at 1 km resolution, and the high‐elevation segment no longer outperforms mid‐elevation areas; at 30 m resolution, a clear majority (11 species) likewise decline, eroding the former elevational advantage. Finer‐scale (30 m) projections further reveal more complex “decrease‐then‐increase” or “increase‐then‐decrease” trajectories, yet dispersal limitation invariably drives an overall contraction in suitable area across species (Figure S1).

### 3.3 Future Bamboo Climate Refuge in Giant Panda Habitat

**FIGURE 4:**
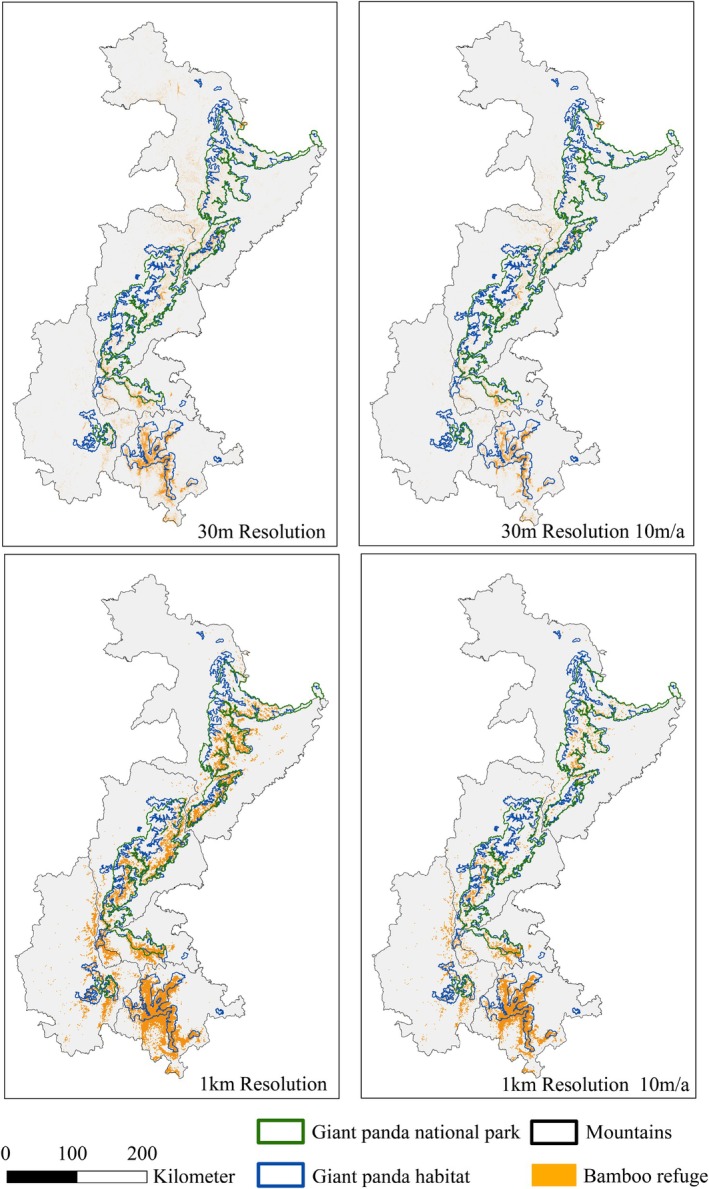
Prediction of climate refuge distribution of GPFB communities of giant pandas in this century.

Integrating projections from all climate models and emission scenarios, the theoretical area of climate refuge for giant‐panda feeding bamboo communities ranges from 1.29 × 10^4^ to 1.37 × 10^4^ km^2^. Yet dispersal limitation truncates the accessible potential refuge to 0.61–0.84 × 10^4^ km^2^, only 44%–65% of the potential total (Figure 4). Spatially, refuge is scattered across the five major mountain systems, with the Liangshan and Minshan ranges harboring the largest shares (16.8% and 12.5%, respectively; 13.8% and 6.8% after accounting for dispersal), whereas Xiaoxiangling holds < 2%. The Giant Panda National Park alone conserves 38%–44% of this refuge, and the overlap with current giant‐panda habitat covers 54.9% of the refuge area, indicating effective protection of high‐elevation zones but leaving low‐elevation portions largely unprotected.

**FIGURE 5:**
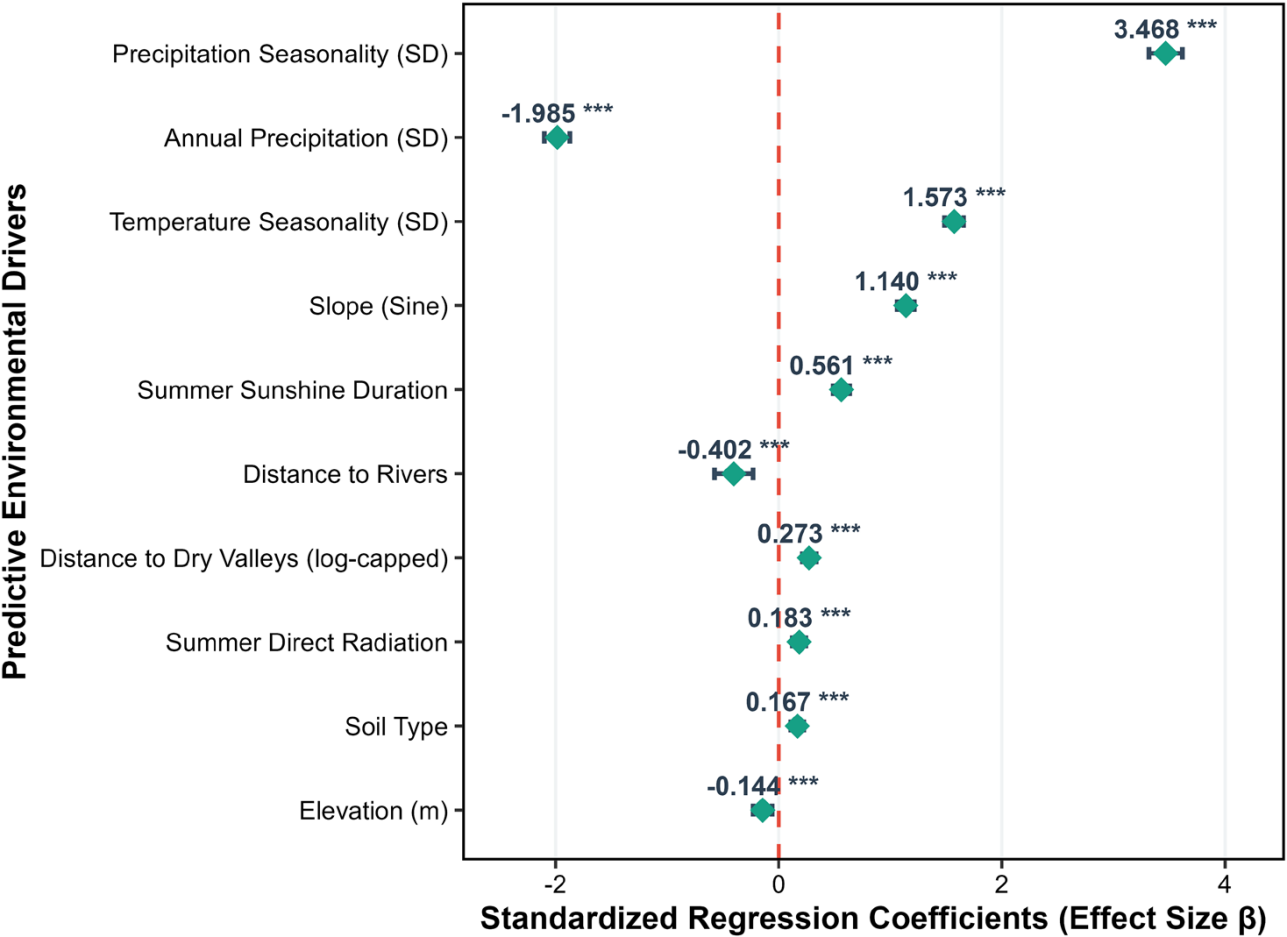
Standardized regression coefficients (β) and 95% confidence intervals of environmental determinants driving bamboo climate refugia. Predictors are arranged hierarchically along the y‐axis according to the absolute value of their effect sizes. The emerald diamonds denote the standardized regression coefficients, and the error bars represent the 95% confidence intervals. The vertical red dashed line indicating zero (β = 0) serves as the baseline for statistical significance; error bars that do not cross this line denote statistically significant effects (*p* < 0.001, as indicated by ***). Predictors with positive coefficients (β > 0) positively contribute to the probability of refugium formation, whereas those with negative coefficients (β < 0) act as inhibitors. All continuous environmental variables were standardized using *Z*‐score transformation prior to modeling. Model evaluation is based on a rigorous 10‐fold cross‐validation framework (mean AUC = 0.8905 ± 0.0107).

The 10‐fold cross‐validation logistic regression model rigorously unveiled that the spatial emergence of bamboo climate refugia is multi‐dimensionally governed by the interplay of long‐term climatic stability, macroclimate topography, hydrological accessibility, and localized solar radiation regimes (Figure 5). Bioclimatically, multi‐scenario climatic variances exerted the most predominant yet divergent influences on refugia formation. Specifically, precipitation seasonality stability (sd_bio15) emerged as the most critical positive driver (β = +3.468, *p* < 0.001), indicating that areas with dynamic seasonal precipitation fluctuations across scenarios are highly prone to harbor stable bamboo refugia. Conversely, a prominent negative effect was detected for annual precipitation variability (sd_bio12, β = −1.985, *p* < 0.001), demonstrating that long‐term baseline precipitation constancy is a prerequisite for refugia persistence. Temperature seasonality variance (sd_bio4) also exhibited a significant positive relationship (β = +1.573, *p* < 0.001), suggesting that localized thermodynamic fluctuations might offer favorable macroclimatic buffering pockets. Topographic complexity and radiation regimes further facilitated microclimate buffering. Slope steepness (Slope Sine) demonstrated a strong positive association with refugia occurrence (β = +1.140, *p* < 0.001), implying that complex, steep terrains provide crucial physical shelter against macroclimatic anomalies. Refugia probability was also significantly enhanced by prolonged summer sunshine duration (β = +0.561, *p* < 0.001) and elevated summer direct solar radiation (β = +0.183, *p* < 0.001), alongside a distinct preference for specific soil properties (Soil Type, β = +0.167, *p* < 0.001). In contrast, elevation displayed a weak but statistically significant negative effect (β = −0.144, *p* < 0.001). Water accessibility, evaluated through the refined hydrological variables, played an indispensable role in maintaining refugia. Distance to perennial rivers exhibited a significant negative coefficient (β = −0.402, *p* < 0.001), revealing that proximity to stable water sources substantially accentuates refugia establishment. Concurrently, a significant positive coefficient was observed for the distance to dry valleys (β = +0.273, *p* < 0.001), indicating that refugia are strongly constrained to stay away from chronic arid valley segments. Collectively, these high‐resolution (30 m) statistical constraints corroborate that “steep topographies, high hydrological availability, long‐term annual precipitation stability, and distinct seasonal environmental variations” constitute the core environmental attributes defining future climate refugia for giant panda forage bamboo. Statistical results for land cover are summarized in Tables S49 and S50.

### 3.4 Landscape Feasibility and Dispersal Constraints of Climate Refugia

**FIGURE 6:**
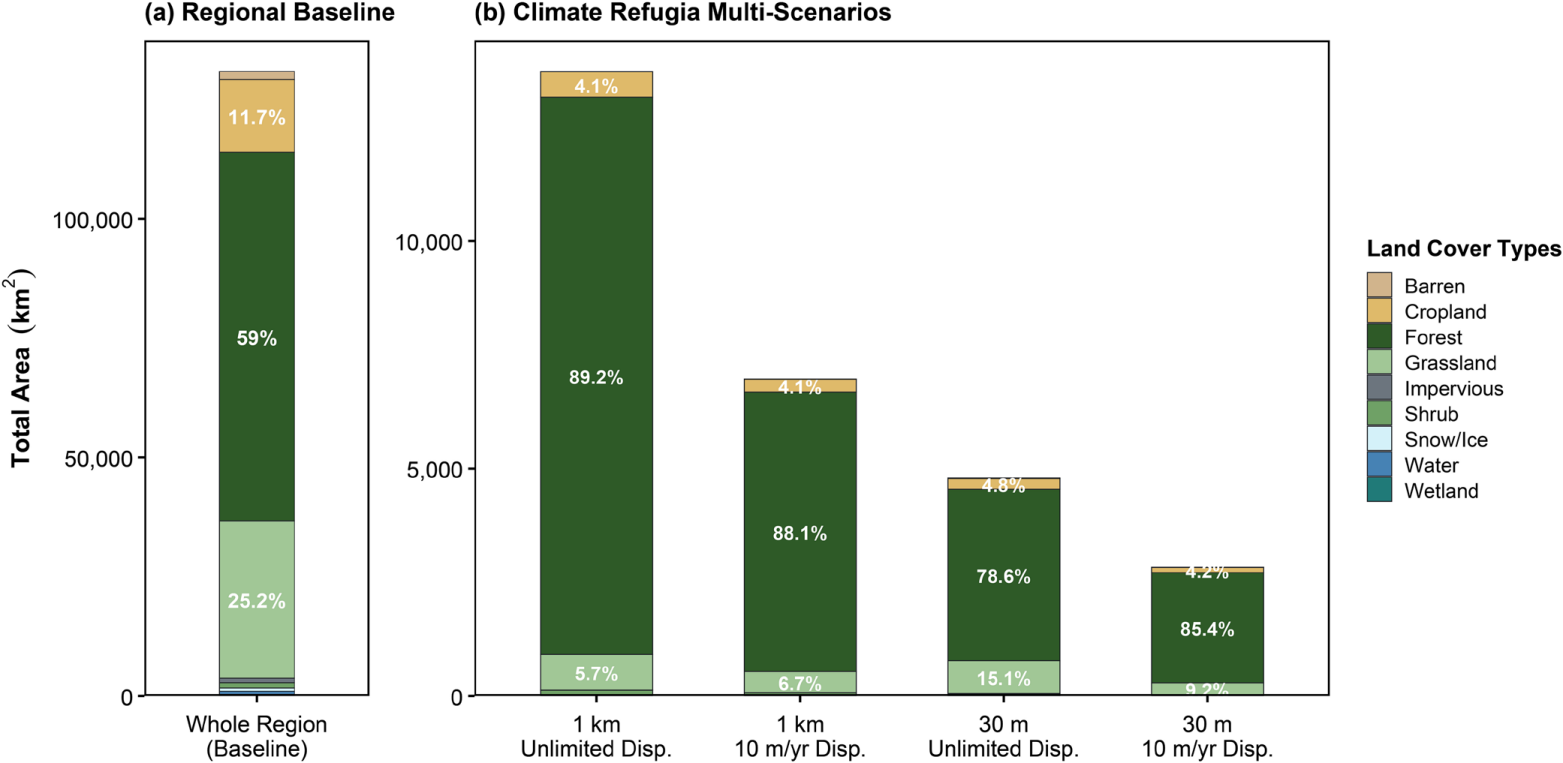
Comparison of landscape composition between the regional baseline and climate refugia under different spatial resolutions and dispersal scenarios. (a) Total area and land cover proportions of the whole study area (baseline). (b) Total area and land cover distributions within climate refugia projected under four scenarios combining grid sizes (1 km and 30 m) and dispersal capacities (Unlimited Dispersal vs. 10 m/year Dispersal). Values within the stacked bars indicate the percentage contribution of major land cover types (> 3%). Note the different scales on the y‐axes between panels (a) and (b).

Landscape composition analysis indicated that the projected climate refugia exhibited pronounced nonrandom environmental filtering characteristics (Figure 6). As the ecological backbone for giant panda habitats, the proportion of forest significantly increased from 59.0% at the regional baseline to 78.6%–89.2% across various refugia scenarios, confirming that forested environments offer higher ecological stability under climate fluctuations. Conversely, anthropogenic disturbance types, such as croplands (which account for 11.7% of the entire region) and impervious surfaces, were heavily filtered out; the proportion of cropland contracted to 4.1%–4.8%, while impervious surfaces were almost completely excluded (< 0.15%), ensuring a high degree of conservation purity within the identified refugia.

Comparison across spatial resolutions revealed a distinct scale‐assimilation effect and microhabitat‐capturing mechanisms. Under the unlimited dispersal scenario, the 1 km coarse resolution tended to assimilate scattered grasslands and shrubs into the matrix of continuous forests due to the majority‐pixel effect, yielding an inflated forest proportion of 89.2% and omitting key habitat heterogeneity. In sharp contrast, the 30 m high‐resolution data successfully captured complex terrain configurations, modulating the forest proportion to a more realistic 78.6% while successfully identifying critical non‐forest microhabitats, as evidenced by the dramatic increase in grassland proportion from 5.7% (at 1 km) to 15.1% (at 30 m).

The incorporation of a conservative “10 m/yr” dispersal limitation exerted a severe spatial trimming effect on the refugia, whereas the internal landscape proportions remained relatively stable. Dispersal constraint acted as a spatial filter, precipitating a drastic shrinkage in the absolute area of available refugia (e.g., a substantial drop in total area at the 30 m scale), yet inducing only minor shifts in the percentage contributions of major land covers (e.g., forest shifting to 85.4%). This pattern implies that while realistic dispersal barriers and intrinsic migration capacities drastically curtail the absolute extent of accessible future refugia, the remaining accessible segments maintain consistent habitat quality. These findings provide vital empirical support for delineating pragmatic and high‐priority spatial conservation redlines.

## 4 Discussion

### 4.1 Patterns of GPFB Community Response to Climate Change

At the community scale, the total suitable habitat of GPFB expands under SSP126–585 scenarios, yet species richness within these habitats declines, generating an “area–diversity paradox”. Owing to inherently limited dispersal capacity, a considerable fraction of climatically suitable pixels remains unreachable by bamboo. Model resolution deeply influences the location and size of future diversity hotspots. At 1 km resolution, GPFB communities exhibit a highly consistent spatial pattern across scenarios, with high‐diversity cores concentrated in central Minshan, central Qionglai, central Liangshan, or along the eastern margin of the study area (only under SSP126). In contrast, 30 m resolution yields more heterogeneous patterns that hotspots shift to the northwestern mountains and the eastern edge of the Tibetan Plateau. For montane species with restricted dispersal, multi‐scale ensemble forecasting is therefore indispensable when assessing community level responses to climate change.

The projected decline in bamboo species richness coupled with the spatial expansion of monodominant bamboo may undermine the stability of subalpine forest ecosystems (Fadrique et al. 2021). Reduced bamboo diversity erodes the seasonal dietary flexibility of obligate bamboo feeders such as the giant panda and red panda (*Ailuropoda styani*) (Hu et al. 2024), as observed in other montane specialists (Kathayat et al. 2020). Simultaneous contraction of historical bamboo groves, colonization of new areas, and elevational redistribution will likely alter existing forest vegetation structure and cascade to broader biodiversity impacts (Gu et al. 2021; Shipley et al. 2022). We advocate integrating remote sensing and LiDAR‐based rapid monitoring to increase the frequency of assessments on bamboo distribution. Thereby facilitating the transition of Giant Panda National Park management from single‐species conservation to resilience‐oriented ecosystem governance.

### 4.2 The Function of Mountains in Bamboo Response to Climate Change

The giant panda's core range almost perfectly overlays the Hengduan Mountains, a topographically complex region forced by both Indian and Pacific monsoons (wet summers, dry winters) and an elevation gradient spanning 219–7469 m a.s.l. (Wu et al. 2013). This mountainous template offers an exceptional natural laboratory for dissecting how species' distribution responds to climate change. SDM models indicate that the 3000–4500 m mountain belt currently supplies optimum climatic‐buffering space for bamboo taxa. When dispersal limitation is ignored, seven of twenty focal species are projected to expand contiguously within this band. However, the montane “elevational advantage” collapses once potential vegetative dispersal limits of bamboo are considered. Therefore, whether the mountain buffering effect can actually materialize depends on the species' real migration capacity rather than the elevational gradient itself. A core strategy for future conservation of giant‐panda food bamboos could be to create and widen effective pathways that allow bamboo to spread into suitable habitat, introducing targeted assisted interventions where necessary to help the mountain buffering effect take hold.

Additionally, fine‐resolution projections reveal that, owing to the complexity of mountain topography and climate, not all GPFB respond to climate change through a simple areal contraction. At 1 km resolution every species shows net habitat loss, yet at 30 m resolution only 11 of 20 species decline (Figure S1).

Vertical shift patterns are likewise multidirectional: most northern bamboo taxa expand into higher‐latitude zones, southern bamboo toward higher‐elevation areas in the west or south, while under the SSP126 scenario some even spread to lower elevations (Figure S2). This intricacy mirrors the finding that Chinese montane seed plants do not universally migrate upslope as temperatures rise (Zu et al. 2023), underscoring that ascent is not the sole strategy for coping with climate change in complex mountain terrain. Under continuous global warming, species whose climatic niches shift downward are likely to face immediate habitat loss or severe human‐wildlife conflict as their potential ranges overlap with human‐dominated landscapes, whereas those moving upslope could face heightened habitat‐loss risk.

### 4.3 The Distribution and Causes of Mountain Bamboo Climate Refuge

South‐west China and northern Viet Nam long served as enduring refuge for many Paleogene–Neogene and even older relict plants (Tang, Matsui, et al. 2018). Such refuges are sites whose buffering attributes allowed distinct genetic lineages to persist through Tertiary or Quaternary climate oscillations, and can also be defined as the minimal geographic areas occupied by a species during glacial–interglacial contractions (Stewart et al. 2009; Hampe et al. 2013). Here we adopt a conservation‐biology perspective: a refuge is any area that, within a given time‐frame, enables a species to escape specific threatening factors (Selwood and Zimmer 2020). Securing this climate refuge is vital for the long‐term health of biodiversity and ecosystems (Tang, Matsui, et al. 2018; Selwood and Zimmer 2020). The giant panda itself is a relict species confined to the mountains of south‐western China. From the late Neogene to the Middle Pleistocene, the small‐sized panda diverged from its ancestors, evolved into the modern population, and became the emblem of the Middle–Late Pleistocene Ailuropoda–Stegodon fauna (Pan et al. 2001). Today's pandas are thought to have originated during the Holocene hypsithermal (Hu et al. 2024), coinciding with the Neolithic expansion of humans. Subsequent agricultural and developmental pressure progressively compressed their range into the narrow belt of mountains between the southern slopes of the Qinling and the Daliang Shan (Pan et al. 2001). Although we only identify climatic refuge for GPFB under 21st century change, the long‐term stability of bamboo distribution is critical for a bamboo‐specialist species and may ultimately determine the panda's final persistence in these very areas (Han et al. 2019).

The deeply incised, rugged topography of the Hengduan Mountains in southwestern China creates a wide and complex mosaic of microclimates (Rahbek, Borregaard, Colwell, et al. 2019; Zu and Wang 2022), providing stable refugia for bamboo communities facing climate change (Figure 4). Across all climate models and emission scenarios, the theoretical area of climate refugia for giant panda‐foraging bamboo ranges from 1.29 × 10^4^ to 1.37 × 10^4^ km^2^. However, due to dispersal limitations, the actual accessible refugia account for only 44%–65% of the total potential area (Figure 4). Spatially, these refugia are patchily distributed across five major mountain ranges, with the Liangshan and Minshan mountains holding the largest shares (16.8% and 12.5%, respectively, without considering dispersal; decreasing to 13.8% and 6.8% when dispersal is integrated), while the Xiaoxiangling mountains account for less than 2%.

Mechanistic analysis corroborates that the spatial configuration of these refugia is strictly nonrandom, being interactively controlled by multidimensional biophysical constraints. First, multi‐scenario climatic variance establishes the foundational bioclimatic envelope: while baseline precipitation constancy is a prerequisite for long‐term refugia persistence, high seasonal environmental variations in temperature and precipitation across emission pathways amplify the probability of refugia formation. Second, topographic complexity delivers critical physical shelter; steep landscapes effectively decouple local microenvironments from regional macroclimatic anomalies, creating localized buffering pockets that are further enhanced by prolonged summer sunshine and elevated direct solar radiation. Finally, hydrological availability is indispensable in mitigating localized aridization risks, driving a distribution pattern whereby future bamboo sanctuaries are strictly confined to perennial river networks while systematically remaining distant from chronically arid valley floors.

By integrating current land‐use data to assess the realistic feasibility of the identified refugia, landscape composition analysis reveals an environmental “filtering mechanism” (Figure 6). While forests, serving as the primary landscape matrix, account for 58.99% of the entire study area, their proportion increases significantly to 78.64%–89.18% within the identified refugia zones. In the species distribution models, soil type exerts a significant positive driving effect on the distribution of bamboo communities (β = +0.167, *p* < 0.001). This environmental constraint naturally isolates bamboo refugia from areas of intensive human disturbance. Furthermore, the dense forest canopy and complex structure possess excellent microclimate regulation capacities, thereby providing relatively stable biophysical shelter for bamboo communities. In contrast, anthropogenic land‐use types, such as cropland and impervious surfaces, constitute extremely low proportions within the refugia (below 4% and 0.13%, respectively). This demonstrates the validity and conservation value of our predictions—namely, even without the direct inclusion of human disturbance variables, the predictions driven by natural environmental factors did not erroneously place refugia within regions of dense human activity.

In addition, comparisons across different spatial resolutions reveal a pronounced scale dependency. The high‐resolution (30 m) framework effectively captures microrefugia such as local topographic depressions, ravines, and riparian zones, whereas lower resolutions produce a “spatial smoothing effect” that conceals these microclimatic patches. This scale‐dependent variance indicates that utilizing high‐resolution modeling is crucial for accurately evaluating the microecological corridors and diversified foraging patches indispensable to giant pandas.

When integrating realistic biological dispersal limitations (10 m/year), the absolute area of the refugia undergoes a substantial contraction (e.g., a 35.7% loss of forest refugia at the 30 m scale), although the internal landscape composition proportions remain relatively stable (with forest cover adjusting slightly to 85.43%). This divergence indicates that real‐world topographic resistance and intrinsic migration bottlenecks severely curtail the actual accessible refugium area for bamboo species. Without sufficient landscape connectivity, many potentially suitable bioclimatic refugia will face functional isolation due to the inability of species to colonize them. Consequently, future conservation planning must shift its focus from broad, idealized bioclimatic envelope analyses toward geospatial planning based on realistic accessibility.

The future viability of bamboos, giant‐panda habitat and panda populations under climate change will be jointly determined by their ecophysiological traits and the magnitude of the climatic shift (Malanoski et al. 2024). Moreover, bamboo‐community refugia are far more than mere “Panda's natural lunchroom.” These refuges constitute natural gene banks that safeguard both species‐level and genetic diversity within bamboos (Stewart et al. 2009). In addition to supplying forage for endangered species such as the giant panda, red panda and so on (Pan et al. 2001), bamboos are dominant understorey components of sub‐alpine forests across the mountains of south‐western China (Gu et al. 2021; Shipley et al. 2022). Furthermore, bamboos deliver multiple ecosystem services (Nfornkah et al. 2023): they conserve soil and water (Gu et al. 2021), regulate hydrological cycles and buffer microclimate (Fadrique et al. 2021). Therefore, protecting bamboo forest helps mountain landscapes cope with climate‐driven extreme events and enhances the resilience and stability of the broader forest ecosystem (Naderi and Fallahi Akhlamad 2025).

From the perspective of conservation managers, currently, the Giant Panda National Park covers 38.46%–44.11% of these refugia, and the overlap between the refugia and existing giant panda habitats constitutes 54.9% of the total refugium area. Notably, both the giant panda habitats and bamboo refugia within the Liangshan mountain range lie outside the protection and management boundaries of the Giant Panda National Park. This means that protecting bamboo resources will require the national park and local communities to act in concert, tailoring diversified, site‐specific strategies (Shang et al. 2024), specifically focusing on establishing ecological corridors that bridge the gap between idealized climatic suitability and actual geographic accessibility.

### 4.4 Limitations and Further Considerations

There are limitations to our study. First, SDM prediction results are uncertain from a single‐species perspective. GCM models, SSP scenarios, and prediction resolutions all cause uncertainty because the main drivers of bamboo species distribution are climate‐related factors, so the factors that cause climate change will cause changes in distribution predictions. This uncertainty is undeniable; for instance, while our dual‐GCM approach captures key regional temperature and moisture bounds, it remains a conservative projection that does not span the full multi‐model envelope of the CMIP6 archive. This means that in the future we will need refined predictions based on actual climate change processes on small timescales that can help achieve effective conservation management. Thus, we will focus on the changes at the community level and the distribution of refuge (bamboo stable distribution area) under different climate change scenarios (Selwood and Zimmer 2020). Such results are stable areas in climate variability and have reliability.

The downscaling method adopted in this study can simulate the mountain climate and the law of climate change. However, compared with the current climate data, the data after downscaling is higher than the actual value on the whole, and the difference between the values of different months from January to December shows a bell curve, which means that the downscaling method also magnifies the climate differences between seasons while highlighting the microclimate caused by regional altitude differences. In the future, more climate detection equipment can be placed in panda habitats at different altitudes and latitudes to obtain more detailed climate data and correct the results of downscaling models.

The bamboo occurrence data used in this study were derived from the Fourth National Giant Panda Survey (2011–2014). This introduces two potential limitations. First, there is a temporal mismatch between the bamboo survey period and the baseline climate data used for future projections, which was sourced from a unified global database. Second, particularly anthropogenic impacts may have undergone non‐negligible changes over the past decade. To maintain rigorous taxonomic validation across decadal intervals, our research team carried out targeted field ground‐truthing from September to November 2022 across major giant panda core habitats in Sichuan. Ground surveys spanned key protected areas within the Minshan, Qionglai, Daxiangling, and Liangshan mountain ranges. The field‐verified taxonomic assignments and spatial persistence profiles highly converged with the baseline data from the 4th National Survey, ensuring high fidelity in species identification for our predictive modeling. However, this dataset currently remains the most comprehensive, high‐resolution, and recent field‐verified inventory of giant panda bamboo forage available in China, as the fifth national survey is still under preparation. Furthermore, from an ecological standpoint, bamboo species possess exceptionally long generation times, typically flowering and dying back only every 30 to 60 years. Due to this slow life cycle and stable vegetative growth, macroscale distribution shifts of bamboo are relatively buffered compared to other fast‐growing species, and historical baseline patterns remain highly informative for long‐term trend forecasting (Li et al. 2020).

In addition, the current national survey of giant panda forage bamboos primarily focused on areas within the well‐documented giant panda habitats (typically above 1500 m), leaving a spatial data gap for bamboo distributions at lower elevations. While giant pandas themselves are widely excluded from these low‐altitude areas due to intense human activities, certain bamboo species do persist there. Excluding these low‐elevation bamboo records may introduce truncation bias into our ecological niche models, particularly when projecting the potential upslope migration of bamboo ranges driven by climate change. It is worth noting that while current human presence drastically limits the realized niche of giant pandas, it does not entirely inhibit the macroclimatic suitability and dispersal potential of the bamboo itself. Therefore, future extensive botanical surveys at lower elevations are warranted.

Lastly, while this study identifies high‐resolution microclimate pockets that could potentially serve as long‐term refuges or migration corridors for bamboo, it does not fully incorporate dynamic human disturbances or real‐time land‐use changes. So, incorporating spatially explicit land cover layers as a post‐modeling filter would be a valuable next step to reconcile these climate‐driven potential trends with real‐world conservation planning. We emphasize that our model functions at a macro‐ecological scale to delineate the fundamental climatic suitability and long‐term trends—essentially mapping out where ecological opportunities and evolutionary rescue might exist, rather than immediate, on‐the‐ground spatial zoning plans. In practice, microhabitat availability is strictly constrained by real‐world human presence, infrastructure, and socioeconomic dynamics. Nonetheless, our findings provide critical optimism, indicating a high probability that giant pandas' food resources will remain persistently available and stable under climate change, offering clear entry points for adaptive management. Moreover, this study assumes a constant bamboo diffusion rate of 10 m per year for all bamboo species (Liese and Köhl 2015), which may not accurately reflect inter‐species diffusion differences in real environments. This is primarily due to the limited understanding of the physiological and ecological mechanisms of bamboo responses to climate change. Further basic research in this area is crucial for improving predictions of bamboo habitat responses to climate change.

## 5 Conclusion

This study establishes a high‐resolution workflow to track the climate‐driven redistribution of understory mountain biodiversity by coupling a statistical delta‐downscaling framework with optimized MaxEnt modeling. By evaluating 20 giant panda forage bamboo species under multiple 21st‐century emission pathways and dispersal scenarios, we quantified how complex rugged terrain modulates ecological responses and alters conservation priorities. The definitive conclusions are as follows: First, fine‐grained resolution is indispensable for eliminating scale‐assimilation bias in montane SDMs. Moving from a coarse 1 km grid to a 30 m microclimatic layer reveals that while broad bioclimatic envelopes for bamboo appear to expand, community level species richness undergoes a widespread decline. Second, mountain topographies exert multi‐directional buffering effects. Although the 3000–4500 m elevation band functions as a climatic buffer zone under an unlimited dispersal premise, it is strictly bound by biological dispersal limitations. Bamboo's migration lags may need artificial actions to reach idealized future habitats. Furthermore, finer‐scale trajectories demonstrate that montane bamboo species do not respond through uniform monotonic upslope shifts. Instead, their tracking of shifting niches is highly individualized, following multiple directional vectors. Third, future bamboo microrefugia are tightly governed by localized hydro‐topographic filters and exhibit severe conservation gaps. Standardized logistic diagnostics confirm that steep slopes, stable annual precipitation baseline, and adjacent river networks constitute the foundational biophysical footprint of persistent refugia. However, more than half of these modeled sanctuaries, particularly the massive, contiguous core refugia situated in the Liangshan mountain range, currently sit outside the administrative boundaries of the Giant Panda National Park. Consequently, we advocate for an immediate transition from static, single‐species management to a dynamic, climate‐resilient ecosystem governance framework. Conservation planning must urgently implement differentiated management zones inside and outside the national park, focusing on the preservation of unprotected extra‐park refugia and the deployment of assisted migration or ecological corridors to bridge the gap between bioclimatic suitability and actual geographic accessibility. This methodology offers an empirically validated, transferable standard for executing spatial planning and risk‐assessment for threatened mountain ecosystems worldwide.

## Author Contributions

**Xiaotong Shang:** conceptualization (equal), data curation (lead), methodology (lead), software (lead), visualization (lead), writing – original draft (lead), writing – review and editing (lead). **Biao Yang:** funding acquisition (equal), resources (equal), supervision (equal), writing – review and editing (equal). **Qiang Dai:** data curation (equal), formal analysis (equal), funding acquisition (equal), methodology (equal), software (equal). **Han Pan:** data curation (equal), methodology (equal). **Lan Qiu:** data curation (equal), methodology (equal). **Yihong Wang:** data curation (equal), methodology (equal). **Chengcheng Zhang:** data curation (equal), methodology (equal). **Xuyu Yang:** investigation (equal). **Xiaodong Gu:** investigation (equal). **Zhisong Yang:** investigation (equal). **Yang Liu:** funding acquisition (equal), investigation (equal). **Li Zhang:** conceptualization (equal), project administration (equal), resources (equal), supervision (equal).

## Funding

This work was supported by the National Natural Science Foundation of China, 32070520 (QD), 32470535 (BY). Fauna and Flora Baseline Survey of Huanglong Natural Reserve, N5132112023000495 (YL).

## Conflicts of Interest

The authors declare no conflicts of interest.

## Supporting information


**Table S1:** The 20 GPFB species in This Study (With the number of occurrence records > 30).
**Table S2:** TSS and AUC values for the Maxent model evaluation of 20 GPFB species.
**Table S3:** Variable contribution and permutation importance in environmental factors affecting habitat suitability of *P. bissetii* at 30 m resolution.
**Table S4:** Variable contribution and permutation importance in environmental factors affecting habitat suitability of *P. bissetii* at 1 km resolution.
**Table S5:** Variable contribution and permutation importance in environmental factors affecting habitat suitability of *A. faberi* at 30 m resolution.
**Table S6:** Variable contribution and permutation importance in environmental factors affecting habitat suitability of *A. faberi* at 1 km resolution.
**Table S7:** Variable contribution and permutation importance in environmental factors affecting habitat suitability of *A. spanostachya* at 30 m resolution.
**Table S8:** Variable contribution and permutation importance in environmental factors affecting habitat suitability of *A. spanostachya* at 1 km resolution.
**Table S9:** Variable contribution and permutation importance in environmental factors affecting habitat suitability of *C. szechuanensis* at 30 m resolution.
**Table S10:** Variable contribution and permutation importance in environmental factors affecting habitat suitability of *C. szechuanensis* at 1 km resolution.
**Table S11:** Variable contribution and permutation importance in environmental factors affecting habitat suitability of *C. opienensis* at 30 m resolution.
**Table S12:** Variable contribution and permutation importance in environmental factors affecting habitat suitability of *C. opienensis* at 1 km resolution.
**Table S13:** Variable contribution and permutation importance in environmental factors affecting habitat suitability of *C. tumidissinoda* at 30 m resolution.
**Table S14:** Variable contribution and permutation importance in environmental factors affecting habitat suitability of *C. tumidissinoda* at 1 km resolution.
**Table S15:** Variable contribution and permutation importance in environmental factors affecting habitat suitability of *F. denudate* at 30 m resolution.
**Table S16:** Variable contribution and permutation importance in environmental factors affecting habitat suitability of *F. denudate* at 1 km resolution.
**Table S17:** Variable contribution and permutation importance in environmental factors affecting habitat suitability of *F. rufa* at 30 m resolution.
**Table S18:** Variable contribution and permutation importance in environmental factors affecting habitat suitability of *F. rufa* at 1 km resolution.
**Table S19:** Variable contribution and permutation importance in environmental factors affecting habitat suitability of *F. robusta* at 30 m resolution.
**Table S20:** Variable contribution and permutation importance in environmental factors affecting habitat suitability of *F. robusta* at 1 km resolution.
**Table S21:** Variable contribution and permutation importance in environmental factors affecting habitat suitability of *F. nitida* at 30 m resolution.
**Table S22:** Variable contribution and permutation importance in environmental factors affecting habitat suitability of *F. nitida* at 1 km resolution.
**Table S23:** Variable contribution and permutation importance in environmental factors affecting habitat suitability of *F. scabrida* at 30 m resolution.
**Table S24:** Variable contribution and permutation importance in environmental factors affecting habitat suitability of *F. scabrida* at 1 km resolution.
**Table S25:** Variable contribution and permutation importance in environmental factors affecting habitat suitability of *F. ferax* at 30 m resolution.
**Table S26:** Variable contribution and permutation importance in environmental factors affecting habitat suitability of *F. ferax* at 1 km resolution.
**Table S27:** Variable contribution and permutation importance in environmental factors affecting habitat suitability of *F. obliqua* at 30 m resolution.
**Table S28:** Variable contribution and permutation importance in environmental factors affecting habitat suitability of *F. obliqua* at 1 km resolution.
**Table S29:** Variable contribution and permutation importance in environmental factors affecting habitat suitability of *Y. brevipaniculata* at 30 m resolution.
**Table S30:** Variable contribution and permutation importance in environmental factors affecting habitat suitability of *Y. brevipaniculata* at 1 km resolution.
**Table S31:** Variable contribution and permutation importance in environmental factors affecting habitat suitability of *Y. lineolate* at 30 m resolution.
**Table S32:** Variable contribution and permutation importance in environmental factors affecting habitat suitability of *Y. lineolate* at 1 km resolution.
**Table S33:** Variable contribution and permutation importance in environmental factors affecting habitat suitability of *Y. maculate* at 30 m resolution.
**Table S34:** Variable contribution and permutation importance in environmental factors affecting habitat suitability of *Y. maculate* at 1 km resolution.
**Table S35:** Variable contribution and permutation importance in environmental factors affecting habitat suitability of *Y. glauca* at 30 m resolution.
**Table S36:** Variable contribution and permutation importance in environmental factors affecting habitat suitability of *Y. glauca* at 1 km resolution.
**Table S37:** Variable contribution and permutation importance in environmental factors affecting habitat suitability of *Y. ailuropodine* at 1 km resolution.
**Table S38:** Variable contribution and permutation importance in environmental factors affecting habitat suitability of *Y. ailuropodine* at 1 km resolution.
**Table S39:** Variable contribution and permutation importance in environmental factors affecting habitat suitability of *Y. mabianensis* at 30 m resolution.
**Table S40:** Variable contribution and permutation importance in environmental factors affecting habitat suitability of *Y. mabianensis* at 1 km resolution.
**Table S41:** Variable contribution and permutation importance in environmental factors affecting habitat suitability of *Y. dafengdingensis* at 30 m resolution.
**Table S42:** Variable contribution and permutation importance in environmental factors affecting habitat suitability of *Y. dafengdingensis* at 1 km resolution.
**Table S43:** Monthly average temperature data verification from 1970 to 2000.
**Table S44:** Monthly average precipitation data verification from 1970 to 2000.
**Table S45:** Monthly average temperature and monthly precipitation data verification for 2041–2060, 2081–2100 (Model M SSP1‐26).
**Table S46:** Monthly average temperature and monthly precipitation data verification for 2041–2060, 2081–2100 (Model M SSP2‐45).
**Table S47:** Monthly average temperature and monthly precipitation data verification for 2041–2060, 2081–2100 (Model M SSP5‐85).
**Table S48:** Test of the fit degree between the 30 m resolution climate downscaling data and the monthly average temperature and monthly precipitation of 1 km.
**Table S49:** Total area (km^2^) of different land cover classes within the whole study area and mapped climate refugia across multi‐scenarios in 2025.
**Table S50:** Percentage composition (%) of different land cover classes within the whole study area and mapped climate refugia across multi‐scenarios in 2025.
**Table S51:** Statistics of total and high‐altitude (3000–4500 m) distribution characteristics for 20 bamboo species under the SSP1‐2.6 scenario based on the GISS‐E2‐1‐G model.
**Table S52:** Statistics of total and high‐altitude (3000–4500 m) distribution characteristics for 20 bamboo species under the SSP2‐4.5 scenario based on the GISS‐E2‐1‐G model.
**Table S53:** Statistics of total and high‐altitude (3000–4500 m) distribution characteristics for 20 bamboo species under the SSP5‐8.5 scenario based on the GISS‐E2‐1‐G model.
**Table S54:** Statistics of total and high‐altitude (3000–4500 m) distribution characteristics for 20 bamboo species under the SSP1‐2.6 scenario based on the MRI‐ESM2‐0 model.
**Table S55:** Statistics of total and high‐altitude (3000–4500 m) distribution characteristics for 20 bamboo species under the SSP2‐4.5 scenario based on the MRI‐ESM2‐0 model.
**Table S56:** Statistics of total and high‐altitude (3000–4500 m) distribution characteristics for 20 bamboo species under the SSP5‐8.5 scenario based on the MRI‐ESM2‐0 model.
**Figure S1:** Changes in Suitable Habitat for Bamboo Species. The alterations in suitable habitat areas for a total of 20 bamboo species, both with and without diffusion constraints. To elucidate these changes, we subtract the projected habitat areas based on two climate models and three climate scenarios from the habitat distribution observed in the year 2000. The resulting difference is expressed as a proportion relative to the original area. For a clearer understanding, we calculate the mean change proportion along with its corresponding 95% confidence interval. In the figure, you will find black dots denoting the mean change proportion, while the yellow lines encompass the 95% confidence interval. The gray vertical lines pinpoint positions where no change has occurred, indicated by a value of 0. Specifically, panels a‐b (without diffusion constraints) and c‐d (with diffusion constraints) portray alterations in suitable habitat proportions for the 20 bamboo species at a 1‐km resolution, both during the middle and toward the end of this century. Similarly, panels e‐f (without diffusion constraints) and g‐h (with diffusion constraints) provide insights into the changes in suitable habitat proportis for the same 20 bamboo species, also at a 1‐km resolution, across the middle and end of this century. note:We use “FSH” to denote future suitable habitat and “FSHUDL” to represent future suitable habitat under diffusion constraints.
**Figure S2:** Visually depicts the migration direction and distance of centroid positions for suitable habitats of 20 bamboo species. This figure specifically focuses on changes in suitable habitat centroids at a 30 m resolution while considering diffusion constraints. (a–f) Correspond to scenarios for the mid and late stages of this century, utilizing the SSP1‐26, SSP2‐45, and SSP5‐85 scenarios with the MRI‐ESM2‐0 climate model. (g–l) Illustrate scenarios for the mid and late stages of this century, employing the SSP1‐26, SSP2‐45, and SSP5‐85 scenarios with the GISS‐E2‐1‐G climate model.
**Figure S3:** Response of suitable habitat for *P. bissetii* to climate change and the impact of dispersal ability on its distribution at 1 km and 30 m resolutions. The left side shows the prediction results with a resolution of 1 km, and the right side shows the prediction results with a resolution of 30 m. 2000 represents the situation at the beginning of this century, “1” denotes the middle of the century (2041–2060) and “2” denotes the end of the century (2081–2100). G represents the GISS‐E2‐1‐G model, M represents the MRI‐ESM2‐0 model, 26 represents the sharing economy path SSP1‐26, 45 represents the sharing economy path SSP2‐45, 85 represents the sharing economy path SSP5‐85. Gray represents the study area, blue indicates suitable habitats, and pink denotes suitable habitats that are inaccessible under a dispersal limitation of 10 m/yr. “1 km” and “30 m” represent the spatial resolutions.
**Figure S4:** Response of suitable habitat for *A. faberi* to climate change and the impact of dispersal ability on its distribution at 1 km and 30 m resolutions. The left side shows the prediction results with a resolution of 1 km, and the right side shows the prediction results with a resolution of 30 m. 2000 represents the situation at the beginning of this century, “1” denotes the middle of the century (2041–2060) and “2” denotes the end of the century (2081–2100). G represents the GISS‐E2‐1‐G model, M represents the MRI‐ESM2‐0 model, 26 represents the sharing economy path SSP1‐26, 45 represents the sharing economy path SSP2‐45, 85 represents the sharing economy path SSP5‐85. Gray represents the study area, blue indicates suitable habitats, and pink denotes suitable habitats that are inaccessible under a dispersal limitation of 10 m/yr. “1 km” and “30 m” represent the spatial resolutions.
**Figure S5:** Response of suitable habitat for *A. spanostachya* to climate change and the impact of dispersal ability on its distribution at 1 km and 30 m resolutions. The left side shows the prediction results with a resolution of 1 km, and the right side shows the prediction results with a resolution of 30 m. 2000 represents the situation at the beginning of this century, “1” denotes the middle of the century (2041–2060) and “2” denotes the end of the century (2081–2100). G represents the GISS‐E2‐1‐G model, M represents the MRI‐ESM2‐0 model, 26 represents the sharing economy path SSP1‐26, 45 represents the sharing economy path SSP2‐45, 85 represents the sharing economy path SSP5‐85. Gray represents the study area, blue indicates suitable habitats, and pink denotes suitable habitats that are inaccessible under a dispersal limitation of 10 m/yr. “1 km” and “30 m” represent the spatial resolutions.
**Figure S6:** Response of suitable habitat for *C. szechuanensis* to climate change and the impact of dispersal ability on its distribution at 1 km and 30 m resolutions. The left side shows the prediction results with a resolution of 1 km, and the right side shows the prediction results with a resolution of 30 m. 2000 represents the situation at the beginning of this century, “1” denotes the middle of the century (2041–2060) and “2” denotes the end of the century (2081–2100). G represents the GISS‐E2‐1‐G model, M represents the MRI‐ESM2‐0 model, 26 represents the sharing economy path SSP1‐26, 45 represents the sharing economy path SSP2‐45, 85 represents the sharing economy path SSP5‐85. Gray represents the study area, blue indicates suitable habitats, and pink denotes suitable habitats that are inaccessible under a dispersal limitation of 10 m/yr. “1 km” and “30 m” represent the spatial resolutions.
**Figure S7:** Response of suitable habitat for *C. opienensis* to climate change and the impact of dispersal ability on its distribution at 1 km and 30 m resolutions. The left side shows the prediction results with a resolution of 1 km, and the right side shows the prediction results with a resolution of 30 m. 2000 represents the situation at the beginning of this century, “1” denotes the middle of the century (2041–2060) and “2” denotes the end of the century (2081–2100). G represents the GISS‐E2‐1‐G model, M represents the MRI‐ESM2‐0 model, 26 represents the sharing economy path SSP1‐26, 45 represents the sharing economy path SSP2‐45, 85 represents the sharing economy path SSP5‐85. Gray represents the study area, blue indicates suitable habitats, and pink denotes suitable habitats that are inaccessible under a dispersal limitation of 10 m/yr. “1 km” and “30 m” represent the spatial resolutions.
**Figure S8:** Response of suitable habitat for *C. tumidissinoda* to climate change and the impact of dispersal ability on its distribution at 1 km and 30 m resolutions. The left side shows the prediction results with a resolution of 1 km, and the right side shows the prediction results with a resolution of 30 m. 2000 represents the situation at the beginning of this century, “1” denotes the middle of the century (2041–2060) and “2” denotes the end of the century (2081–2100). G represents the GISS‐E2‐1‐G model, M represents the MRI‐ESM2‐0 model, 26 represents the sharing economy path SSP1‐26, 45 represents the sharing economy path SSP2‐45, 85 represents the sharing economy path SSP5‐85. Gray represents the study area, blue indicates suitable habitats, and pink denotes suitable habitats that are inaccessible under a dispersal limitation of 10 m/yr. “1 km” and “30 m” represent the spatial resolutions.
**Figure S9:** Response of suitable habitat for *F. denudate* to climate change and the impact of dispersal ability on its distribution at 1 km and 30 m resolutions. The left side shows the prediction results with a resolution of 1 km, and the right side shows the prediction results with a resolution of 30 m. 2000 represents the situation at the beginning of this century, “1” denotes the middle of the century (2041–2060) and “2” denotes the end of the century (2081–2100). G represents the GISS‐E2‐1‐G model, M represents the MRI‐ESM2‐0 model, 26 represents the sharing economy path SSP1‐26, 45 represents the sharing economy path SSP2‐45, 85 represents the sharing economy path SSP5‐85. Gray represents the study area, blue indicates suitable habitats, and pink denotes suitable habitats that are inaccessible under a dispersal limitation of 10 m/yr. “1 km” and “30 m” represent the spatial resolutions. Gray represents the study area, blue indicates suitable habitats, and pink denotes suitable habitats that are inaccessible under a dispersal limitation of 10 m/yr. “1 km” and “30 m” represent the spatial resolutions.
**Figure S10:** Response of suitable habitat for *F. rufa* to climate change and the impact of dispersal ability on its distribution at 1 km and 30 m resolutions. The left side shows the prediction results with a resolution of 1 km, and the right side shows the prediction results with a resolution of 30 m. 2000 represents the situation at the beginning of this century, “1” denotes the middle of the century (2041–2060) and “2” denotes the end of the century (2081–2100). G represents the GISS‐E2‐1‐G model, M represents the MRI‐ESM2‐0 model, 26 represents the sharing economy path SSP1‐26, 45 represents the sharing economy path SSP2‐45, 85 represents the sharing economy path SSP5‐85. Gray represents the study area, blue indicates suitable habitats, and pink denotes suitable habitats that are inaccessible under a dispersal limitation of 10 m/yr. “1 km” and “30 m” represent the spatial resolutions.
**Figure S11:** Response of suitable habitat for *F. robusta* to climate change and the impact of dispersal ability on its distribution at 1 km and 30 m resolutions. The left side shows the prediction results with a resolution of 1 km, and the right side shows the prediction results with a resolution of 30 m. 2000 represents the situation at the beginning of this century, “1” denotes the middle of the century (2041–2060) and “2” denotes the end of the century (2081–2100). G represents the GISS‐E2‐1‐G model, M represents the MRI‐ESM2‐0 model, 26 represents the sharing economy path SSP1‐26, 45 represents the sharing economy path SSP2‐45, 85 represents the sharing economy path SSP5‐85. Gray represents the study area, blue indicates suitable habitats, and pink denotes suitable habitats that are inaccessible under a dispersal limitation of 10 m/yr. “1 km” and “30 m” represent the spatial resolutions.
**Figure S12:** Response of suitable habitat for *F. nitida* to climate change and the impact of dispersal ability on its distribution at 1 km and 30 m resolutions. The left side shows the prediction results with a resolution of 1 km, and the right side shows the prediction results with a resolution of 30 m. 2000 represents the situation at the beginning of this century, “1” denotes the middle of the century (2041–2060) and “2” denotes the end of the century (2081–2100). G represents the GISS‐E2‐1‐G model, M represents the MRI‐ESM2‐0 model, 26 represents the sharing economy path SSP1‐26, 45 represents the sharing economy path SSP2‐45, 85 represents the sharing economy path SSP5‐85. Gray represents the study area, blue indicates suitable habitats, and pink denotes suitable habitats that are inaccessible under a dispersal limitation of 10 m/yr. “1 km” and “30 m” represent the spatial resolutions.
**Figure S13:** Response of suitable habitat for *F. scabrida* to climate change and the impact of dispersal ability on its distribution at 1 km and 30 m resolutions. The left side shows the prediction results with a resolution of 1 km, and the right side shows the prediction results with a resolution of 30 m. 2000 represents the situation at the beginning of this century, “1” denotes the middle of the century (2041–2060) and “2” denotes the end of the century (2081–2100). G represents the GISS‐E2‐1‐G model, M represents the MRI‐ESM2‐0 model, 26 represents the sharing economy path SSP1‐26, 45 represents the sharing economy path SSP2‐45, 85 represents the sharing economy path SSP5‐85. Gray represents the study area, blue indicates suitable habitats, and pink denotes suitable habitats that are inaccessible under a dispersal limitation of 10 m/yr. “1 km” and “30 m” represent the spatial resolutions.
**Figure S14:** Response of suitable habitat for *F. ferax* to climate change and the impact of dispersal ability on its distribution at 1 km and 30 m resolutions. The left side shows the prediction results with a resolution of 1 km, and the right side shows the prediction results with a resolution of 30 m. 2000 represents the situation at the beginning of this century, “1” denotes the middle of the century (2041–2060) and “2” denotes the end of the century (2081–2100). G represents the GISS‐E2‐1‐G model, M represents the MRI‐ESM2‐0 model, 26 represents the sharing economy path SSP1‐26, 45 represents the sharing economy path SSP2‐45, 85 represents the sharing economy path SSP5‐85. Gray represents the study area, blue indicates suitable habitats, and pink denotes suitable habitats that are inaccessible under a dispersal limitation of 10 m/yr. “1 km” and “30 m” represent the spatial resolutions.
**Figure S15:** Response of suitable habitat for *F. obliqua* to climate change and the impact of dispersal ability on its distribution at 1 km and 30 m resolutions. The left side shows the prediction results with a resolution of 1 km, and the right side shows the prediction results with a resolution of 30 m. 2000 represents the situation at the beginning of this century, “1” denotes the middle of the century (2041–2060) and “2” denotes the end of the century (2081–2100). G represents the GISS‐E2‐1‐G model, M represents the MRI‐ESM2‐0 model, 26 represents the sharing economy path SSP1‐26, 45 represents the sharing economy path SSP2‐45, 85 represents the sharing economy path SSP5‐85. Gray represents the study area, blue indicates suitable habitats, and pink denotes suitable habitats that are inaccessible under a dispersal limitation of 10 m/yr. “1 km” and “30 m” represent the spatial resolutions.
**Figure S16:** Response of suitable habitat for *Y. brevipaniculata* to climate change and the impact of dispersal ability on its distribution at 1 km and 30 m resolutions. The left side shows the prediction results with a resolution of 1 km, and the right side shows the prediction results with a resolution of 30 m. 2000 represents the situation at the beginning of this century, “1” denotes the middle of the century (2041–2060) and “2” denotes the end of the century (2081–2100). G represents the GISS‐E2‐1‐G model, M represents the MRI‐ESM2‐0 model, 26 represents the sharing economy path SSP1‐26, 45 represents the sharing economy path SSP2‐45, 85 represents the sharing economy path SSP5‐85. Gray represents the study area, blue indicates suitable habitats, and pink denotes suitable habitats that are inaccessible under a dispersal limitation of 10 m/yr. “1 km” and “30 m” represent the spatial resolutions.
**Figure S17:** Response of suitable habitat for *Y. lineolate* to climate change and the impact of dispersal ability on its distribution at 1 km and 30 m resolutions. The left side shows the prediction results with a resolution of 1 km, and the right side shows the prediction results with a resolution of 30 m. 2000 represents the situation at the beginning of this century, “1” denotes the middle of the century (2041–2060) and “2” denotes the end of the century (2081–2100). G represents the GISS‐E2‐1‐G model, M represents the MRI‐ESM2‐0 model, 26 represents the sharing economy path SSP1‐26, 45 represents the sharing economy path SSP2‐45, 85 represents the sharing economy path SSP5‐85. Gray represents the study area, blue indicates suitable habitats, and pink denotes suitable habitats that are inaccessible under a dispersal limitation of 10 m/yr. “1 km” and “30 m” represent the spatial resolutions.
**Figure S18:** Response of suitable habitat for *Y. maculate* to climate change and the impact of dispersal ability on its distribution at 1 km and 30 m resolutions. The left side shows the prediction results with a resolution of 1 km, and the right side shows the prediction results with a resolution of 30 m. 2000 represents the situation at the beginning of this century, “1” denotes the middle of the century (2041–2060) and “2” denotes the end of the century (2081–2100). G represents the GISS‐E2‐1‐G model, M represents the MRI‐ESM2‐0 model, 26 represents the sharing economy path SSP1‐26, 45 represents the sharing economy path SSP2‐45, 85 represents the sharing economy path SSP5‐85. Gray represents the study area, blue indicates suitable habitats, and pink denotes suitable habitats that are inaccessible under a dispersal limitation of 10 m/yr. “1 km” and “30 m” represent the spatial resolutions.
**Figure S19:** Response of suitable habitat for *Y. glauca* to climate change and the impact of dispersal ability on its distribution at 1 km and 30 m resolutions. The left side shows the prediction results with a resolution of 1 km, and the right side shows the prediction results with a resolution of 30 m. 2000 represents the situation at the beginning of this century, “1” denotes the middle of the century (2041–2060) and “2” denotes the end of the century (2081–2100). G represents the GISS‐E2‐1‐G model, M represents the MRI‐ESM2‐0 model, 26 represents the sharing economy path SSP1‐26, 45 represents the sharing economy path SSP2‐45, 85 represents the sharing economy path SSP5‐85. Gray represents the study area, blue indicates suitable habitats, and pink denotes suitable habitats that are inaccessible under a dispersal limitation of 10 m/yr. “1 km” and “30 m” represent the spatial resolutions.
**Figure S20:** Response of suitable habitat for *Y. ailuropodine* to climate change and the impact of dispersal ability on its distribution at 1 km and 30 m resolutions. The left side shows the prediction results with a resolution of 1 km, and the right side shows the prediction results with a resolution of 30 m. 2000 represents the situation at the beginning of this century, “1” denotes the middle of the century (2041–2060) and “2” denotes the end of the century (2081–2100). G represents the GISS‐E2‐1‐G model, M represents the MRI‐ESM2‐0 model, 26 represents the sharing economy path SSP1‐26, 45 represents the sharing economy path SSP2‐45, 85 represents the sharing economy path SSP5‐85. Gray represents the study area, blue indicates suitable habitats, and pink denotes suitable habitats that are inaccessible under a dispersal limitation of 10 m/yr. “1 km” and “30 m” represent the spatial resolutions.
**Figure S21:** Response of suitable habitat for *Y. mabianensis* to climate change and the impact of dispersal ability on its distribution at 1 km and 30 m resolutions. The left side shows the prediction results with a resolution of 1 km, and the right side shows the prediction results with a resolution of 30 m. 2000 represents the situation at the beginning of this century, “1” denotes the middle of the century (2041–2060) and “2” denotes the end of the century (2081–2100). G represents the GISS‐E2‐1‐G model, M represents the MRI‐ESM2‐0 model, 26 represents the sharing economy path SSP1‐26, 45 represents the sharing economy path SSP2‐45, 85 represents the sharing economy path SSP5‐85. Gray represents the study area, blue indicates suitable habitats, and pink denotes suitable habitats that are inaccessible under a dispersal limitation of 10 m/yr. “1 km” and “30 m” represent the spatial resolutions.
**Figure S22:** Response of suitable habitat for *Y. dafengdingensis* to climate change and the impact of dispersal ability on its distribution at 1 km and 30 m resolutions. The left side shows the prediction results with a resolution of 1 km, and the right side shows the prediction results with a resolution of 30 m. 2000 represents the situation at the beginning of this century, “1” denotes the middle of the century (2041–2060) and “2” denotes the end of the century (2081–2100). G represents the GISS‐E2‐1‐G model, M represents the MRI‐ESM2‐0 model, 26 represents the sharing economy path SSP1‐26, 45 represents the sharing economy path SSP2‐45, 85 represents the sharing economy path SSP5‐85. Gray represents the study area, blue indicates suitable habitats, and pink denotes suitable habitats that are inaccessible under a dispersal limitation of 10 m/yr. “1 km” and “30 m” represent the spatial resolutions.

## Data Availability

Data available from the Zenodo Digital Repository: Shang, X., Yang, B., Dai, Q., Pan, H., Qiu, L., Wang, Y., Zhang, C., Yang, X., Gu, X., Yang, Z., Liu, Y., & Zhang, L. (2025). Coupling climate downscaling with species distribution models: A 30‐m resolution workflow to identify microrefugia for giant panda forage bamboos. Zenodo. https://doi.org/10.5281/zenodo.17779606.
