# Supplementary material for: Coupling Climate Downscaling With Species Distribution Models to Identify Potential Climate Refugia for Giant Panda Forage Bamboos
Source: Ecol Evol. 2026 Aug 7;16(8):e74162. doi: 10.1002/ece3.74162 (PMC13451157; doi:10.1002/ece3.74162)
Supplement: Supplementary file 1 [file ECE3-16-e74162-s001.docx]

Table S1 The 20 GPFB species in This Study (With the number of occurrence records > 30)

| Generic name | Species | Number of Sites | Altitude range |
| --- | --- | --- | --- |
| *Phyllostachys* | *Phyllostachys bissetii* | 119 | <1600m |
| *Arundinaria* | *Arundinaria faberi* | 624 | 2300-3500m |
|  | *Arundinaria spanostachya* | 66 | 3200-3900m |
| *Chimonobambusa* | *Chimonobambusa szechuanensis* | 338 | 1400-3000m |
|  | *Chimonobambusa opienensis* | 144 | 1500-2200m(2600m) |
|  | *Chimonobambusa tumidissinoda* | 88 | 1500-2200m |
| *Fargesia* | *Fargesia denudate* | 649 | 1920-3200(3600) |
|  | *Fargesia rufa* | 287 | 1580-2300 (2500) m |
|  | *Fargesia robusta* | 265 | (1200)1700-2800m |
|  | *Fargesia nitida* | 243 | 2450-3200m |
|  | *Fargesia scabrida* | 195 | 1450-2520 |
|  | *Fargesia ferax* | 106 | 1700-2800m |
|  | *Fargesia obliqua* | 69 | 2400-3300m |
| *Yushania* | *Yushania brevipaniculata* | 354 | 1800-3400m |
|  | *Yushania lineolata* | 242 | 2400-3150m |
|  | *Yushania maculata* | 183 | (1800)2700-3500m |
|  | *Yushania glauca* | 93 | 2500-3200m |
|  | *Yushania ailuropodina* | 52 | 2600-3000m |
|  | *Yushania mabianensis* | 35 | 1430-1900m |
|  | *Yushania dafengdingensis* | 32 | 2200-2600m |

Table S2 TSS and AUC values for the Maxent model evaluation of 20 GPFB species.

| Species | Resolution 30m | | Resolution 1km | |
| --- | --- | --- | --- | --- |
|  | AUC | TSS | AUC | TSS |
| *Phyllostachys bissetii* | 0.987687 | 0.921526 | 0.987052 | 0.915446 |
| *Arundinaria faberi* | 0.971758 | 0.838087 | 0.966301 | 0.825705 |
| *Arundinaria spanostachya* | 0.995643 | 0.94159 | 0.998457 | 0.977499 |
| *Chimonobambusa szechuanensis* | 0.990114 | 0.913265 | 0.988722 | 0.919595 |
| *Chimonobambusa opienensis* | 0.994957 | 0.952435 | 0.993956 | 0.943215 |
| *Chimonobambusa tumidissinoda* | 0.998243 | 0.965001 | 0.998641 | 0.9786 |
| *Fargesia denudate* | 0.984974 | 0.89208 | 0.984531 | 0.901939 |
| *Fargesia rufa* | 0.99282 | 0.933102 | 0.994034 | 0.939446 |
| *Fargesia robusta* | 0.991785 | 0.918617 | 0.986289 | 0.913311 |
| *Fargesia nitida* | 0.969105 | 0.830791 | 0.959192 | 0.808242 |
| *Fargesia scabrida* | 0.987575 | 0.919699 | 0.991971 | 0.940049 |
| *Fargesia ferax* | 0.990673 | 0.932856 | 0.981586 | 0.888483 |
| *Fargesia obliqua* | 0.993417 | 0.931654 | 0.990733 | 0.938999 |
| *Yushania brevipaniculata* | 0.975565 | 0.873622 | 0.977615 | 0.860968 |
| *Yushania lineolata* | 0.97814 | 0.869116 | 0.978859 | 0.873538 |
| *Yushania maculata* | 0.993293 | 0.930297 | 0.991481 | 0.935946 |
| *Yushania glauca* | 0.99747 | 0.96448 | 0.99495 | 0.950122 |
| *Yushania ailuropodina* | 0.992443 | 0.932723 | 0.996145 | 0.965243 |
| *Yushania mabianensis* | 0.995143 | 0.959049 | 0.993726 | 0.934086 |
| *Yushania dafengdingensis* | 0.985884 | 0.923085 | 0.992182 | 0.926446 |

Figure S1 Changes in Suitable Habitat for Bamboo Species. The alterations in suitable habitat areas for a total of 20 bamboo species, both with and without diffusion constraints. To elucidate these changes, we subtract the projected habitat areas based on two climate models and three climate scenarios from the habitat distribution observed in the year 2000. The resulting difference is expressed as a proportion relative to the original area. For a clearer understanding, we calculate the mean change proportion along with its corresponding 95% confidence interval. In the figure, you will find black dots denoting the mean change proportion, while the yellow lines encompass the 95% confidence interval. The gray vertical lines pinpoint positions where no change has occurred, indicated by a value of 0. Specifically, panels a-b (without diffusion constraints) and c-d (with diffusion constraints) portray alterations in suitable habitat proportions for the 20 bamboo species at a 1-kilometer resolution, both during the middle and towards the end of this century. Similarly, panels e-f (without diffusion constraints) and g-h (with diffusion constraints) provide insights into the changes in suitable habitat proportis for the same 20 bamboo species, also at a 1-kilometer resolution, across the middle and end of this century. note：We use "FSH" to denote future suitable habitat and "FSHUDL" to represent future suitable habitat under diffusion constraints.

Figure S2 Visually depicts the migration direction and distance of centroid positions for suitable habitats of 20 bamboo species. This figure specifically focuses on changes in suitable habitat centroids at a 30m resolution while considering diffusion constraints. a-f correspond to scenarios for the mid and late stages of this century, utilizing the SSP1-26, SSP2-45, and SSP5-85 scenarios with the MRI-ESM2-0 climate model. g-l illustrate scenarios for the mid and late stages of this century, employing the SSP1-26, SSP2-45, and SSP5-85 scenarios with the GISS-E2-1-G climate model.

SDM results for each bamboo species:

### 1.1.1*Phyllostachys*

#### 1.1.1.1*Phyllostachys bissetii*

Table S3 Variable contribution and permutation importance in environmental factors affecting habitat suitability of *P. bissetii* at 30m resolution.

| Variable | Percent contribution | Permutation importance |
| --- | --- | --- |
| Summer Mean Temperature | 39.54 | 22.35 |
| Spring Precipitation | 16.42 | 27.07 |
| Bio7 Temperature Annual Range | 11.50 | 6.81 |
| Bio15 Precipitation Seasonality | 8.65 | 8.85 |
| Bio12 Annual Precipitation | 7.09 | 6.71 |
| Slope | 4.96 | 13.08 |
| Agrotype | 4.86 | 3.27 |
| Summer Sunshine Duration | 1.78 | 2.76 |
| Distance to other rivers | 1.74 | 1.28 |
| Bio4 Temperature Seasonality | 1.62 | 1.31 |
| Distance to Dry Valleys | 0.66 | 1.17 |
| The amount of direct sunlight in summer | 0.59 | 3.00 |
| The amount of direct sunlight in spring | 0.58 | 2.35 |

Table S4 Variable contribution and permutation importance in environmental factors affecting habitat suitability of *P. bissetii* at 1km resolution.

| Variable | Percent contribution | Permutation importance |
| --- | --- | --- |
| Summer Mean Temperature | 35.37 | 25.90 |
| Spring Precipitation | 17.96 | 27.83 |
| Bio7 Temperature Annual Range | 11.99 | 5.86 |
| Bio12 Annual Precipitation | 10.03 | 9.37 |
| Bio15 Precipitation Seasonality | 7.31 | 8.52 |
| Summer Sunshine Duration | 6.93 | 8.72 |
| Bio4 Temperature Seasonality | 4.42 | 4.50 |
| Agrotype | 2.20 | 2.74 |
| The amount of direct sunlight in summer | 1.20 | 1.92 |
| The amount of direct sunlight in spring | 0.89 | 1.50 |
| Slope | 0.76 | 1.06 |
| Distance to other rivers | 0.60 | 0.87 |
| Distance to Dry Valleys | 0.33 | 1.22 |

Figure S3 Response of suitable habitat for *P. bissetii* to climate change and the impact of dispersal ability on its distribution at 1km and 30m resolutions. The left side shows the prediction results with a resolution of 1km, and the right side shows the prediction results with a resolution of 30m. 2000 represents the situation at the beginning of this century, “1” denotes the middle of the century (2041-2060) and “2” denotes the end of the century (2081-2100). G represents the GISS-E2-1-G model, M represents the MRI-ESM2-0 model, 26 represents the sharing economy path SSP1-26, 45 represents the sharing economy path SSP2-45, 85 represents the sharing economy path SSP5-85. Grey represents the study area, blue indicates suitable habitats, and pink denotes suitable habitats that are inaccessible under a dispersal limitation of 10m/yr. "1km" and "30m" represent the spatial resolutions.

### 1.1.2*Arundinaria*

#### 1.1.2.1*Arundinaria faberi*

Table S5 Variable contribution and permutation importance in environmental factors affecting habitat suitability of *A. faberi* at 30m resolution.

| Variable | Percent contribution | Permutation importance |
| --- | --- | --- |
| Summer Mean Temperature | 19.91 | 27.04 |
| Bio7 Temperature Annual Range | 19.46 | 28.63 |
| Bio15 Precipitation Seasonality | 18.79 | 11.72 |
| Bio4 Temperature Seasonality | 18.39 | 20.56 |
| Slope | 7.70 | 3.09 |
| Agrotype | 6.49 | 3.28 |
| Bio12 Annual Precipitation | 3.17 | 1.84 |
| Spring Precipitation | 1.47 | 0.98 |
| The amount of direct sunlight in summer | 1.13 | 0.91 |
| Summer Sunshine Duration | 1.10 | 0.50 |
| Distance to Dry Valleys | 0.95 | 0.59 |
| The amount of direct sunlight in spring | 0.93 | 0.42 |
| Distance to other rivers | 0.52 | 0.42 |

Table S6 Variable contribution and permutation importance in environmental factors affecting habitat suitability of *A. faberi* at 1km resolution.

| Variable | Percent contribution | Permutation importance |
| --- | --- | --- |
| Summer Mean Temperature | 25.49 | 27.88 |
| Bio4 Temperature Seasonality | 21.45 | 22.47 |
| Bio7 Temperature Annual Range | 19.95 | 19.24 |
| Bio15 Precipitation Seasonality | 18.15 | 15.72 |
| Agrotype | 5.35 | 1.87 |
| Bio12 Annual Precipitation | 3.41 | 4.56 |
| Slope | 1.69 | 1.06 |
| Spring Precipitation | 1.27 | 1.58 |
| The amount of direct sunlight in summer | 0.88 | 2.24 |
| Distance to Dry Valleys | 0.77 | 0.55 |
| Distance to other rivers | 0.64 | 0.32 |
| Summer Sunshine Duration | 0.49 | 1.82 |
| The amount of direct sunlight in spring | 0.46 | 0.69 |

Figure S4 Response of suitable habitat for *A. faberi* to climate change and the impact of dispersal ability on its distribution at 1km and 30m resolutions. The left side shows the prediction results with a resolution of 1km, and the right side shows the prediction results with a resolution of 30m. 2000 represents the situation at the beginning of this century, “1” denotes the middle of the century (2041-2060) and “2” denotes the end of the century (2081-2100). G represents the GISS-E2-1-G model, M represents the MRI-ESM2-0 model, 26 represents the sharing economy path SSP1-26, 45 represents the sharing economy path SSP2-45, 85 represents the sharing economy path SSP5-85. Grey represents the study area, blue indicates suitable habitats, and pink denotes suitable habitats that are inaccessible under a dispersal limitation of 10m/yr. "1km" and "30m" represent the spatial resolutions.

#### 1.1.2.2*Arundinaria spanostachya*

Table S7 Variable contribution and permutation importance in environmental factors affecting habitat suitability of *A. spanostachya* at 30m resolution.

| Variable | Percent contribution | Permutation importance |
| --- | --- | --- |
| Spring Precipitation | 18.81 | 23.71 |
| Bio4 Temperature Seasonality | 17.15 | 0.33 |
| Bio7 Temperature Annual Range | 16.95 | 27.29 |
| Bio12 Annual Precipitation | 15.66 | 12.43 |
| Summer Mean Temperature | 14.22 | 25.66 |
| Agrotype | 4.88 | 3.57 |
| Slope | 4.11 | 2.65 |
| The amount of direct sunlight in summer | 2.48 | 0.31 |
| Bio15 Precipitation Seasonality | 2.37 | 2.19 |
| Distance to other rivers | 1.63 | 1.06 |
| The amount of direct sunlight in spring | 1.02 | 0.42 |
| Summer Sunshine Duration | 0.69 | 0.38 |
| Distance to Dry Valleys | 0.03 | 0.01 |

Table S8 Variable contribution and permutation importance in environmental factors affecting habitat suitability of *A. spanostachya* at 1km resolution.

| Variable | Percent contribution | Permutation importance |
| --- | --- | --- |
| Bio4 Temperature Seasonality | 21.23 | 0.36 |
| Bio7 Temperature Annual Range | 19.45 | 12.33 |
| Spring Precipitation | 17.75 | 35.87 |
| Bio12 Annual Precipitation | 14.40 | 20.70 |
| Summer Mean Temperature | 14.19 | 13.54 |
| Agrotype | 4.30 | 1.44 |
| The amount of direct sunlight in summer | 3.14 | 4.91 |
| Distance to other rivers | 1.21 | 0.60 |
| Slope | 1.19 | 6.27 |
| Bio15 Precipitation Seasonality | 1.12 | 0.31 |
| Summer Sunshine Duration | 1.07 | 2.96 |
| The amount of direct sunlight in spring | 0.93 | 0.72 |
| Distance to Dry Valleys | 0.02 | 0.00 |

Figure S5 Response of suitable habitat for *A. spanostachya* to climate change and the impact of dispersal ability on its distribution at 1km and 30m resolutions. The left side shows the prediction results with a resolution of 1km, and the right side shows the prediction results with a resolution of 30m. 2000 represents the situation at the beginning of this century, “1” denotes the middle of the century (2041-2060) and “2” denotes the end of the century (2081-2100). G represents the GISS-E2-1-G model, M represents the MRI-ESM2-0 model, 26 represents the sharing economy path SSP1-26, 45 represents the sharing economy path SSP2-45, 85 represents the sharing economy path SSP5-85. Grey represents the study area, blue indicates suitable habitats, and pink denotes suitable habitats that are inaccessible under a dispersal limitation of 10m/yr. "1km" and "30m" represent the spatial resolutions.

### 1.1.3*Chimonobambusa*

#### 1.1.3.1*Chimonobambusa szechuanensis*

Table S9 Variable contribution and permutation importance in environmental factors affecting habitat suitability of *C. szechuanensis* at 30m resolution.

| Variable | Percent contribution | Permutation importance |
| --- | --- | --- |
| Bio12 Annual Precipitation | 24.47 | 36.35 |
| Summer Mean Temperature | 23.27 | 17.27 |
| Spring Precipitation | 13.48 | 18.26 |
| Bio4 Temperature Seasonality | 12.46 | 11.00 |
| Bio7 Temperature Annual Range | 9.39 | 5.86 |
| Bio15 Precipitation Seasonality | 6.96 | 3.65 |
| Slope | 2.93 | 2.82 |
| Agrotype | 2.03 | 0.67 |
| The amount of direct sunlight in summer | 1.51 | 1.33 |
| Distance to other rivers | 1.48 | 0.87 |
| The amount of direct sunlight in spring | 1.13 | 1.01 |
| Summer Sunshine Duration | 0.64 | 0.21 |
| Distance to Dry Valleys | 0.25 | 0.70 |

Table S10 Variable contribution and permutation importance in environmental factors affecting habitat suitability of *C. szechuanensis* at 1km resolution.

| Variable | Percent contribution | Permutation importance |
| --- | --- | --- |
| Bio12 Annual Precipitation | 25.28 | 35.53 |
| Summer Mean Temperature | 19.57 | 22.47 |
| Bio4 Temperature Seasonality | 14.09 | 12.97 |
| Bio15 Precipitation Seasonality | 12.47 | 3.62 |
| Spring Precipitation | 10.85 | 10.71 |
| Bio7 Temperature Annual Range | 8.90 | 6.00 |
| Agrotype | 2.94 | 1.62 |
| The amount of direct sunlight in spring | 1.55 | 1.25 |
| Distance to other rivers | 1.32 | 0.58 |
| Slope | 1.29 | 1.75 |
| Summer Sunshine Duration | 0.86 | 1.66 |
| The amount of direct sunlight in summer | 0.67 | 1.10 |
| Distance to Dry Valleys | 0.21 | 0.75 |

Figure S6 Response of suitable habitat for *C. szechuanensis* to climate change and the impact of dispersal ability on its distribution at 1km and 30m resolutions. The left side shows the prediction results with a resolution of 1km, and the right side shows the prediction results with a resolution of 30m. 2000 represents the situation at the beginning of this century, “1” denotes the middle of the century (2041-2060) and “2” denotes the end of the century (2081-2100). G represents the GISS-E2-1-G model, M represents the MRI-ESM2-0 model, 26 represents the sharing economy path SSP1-26, 45 represents the sharing economy path SSP2-45, 85 represents the sharing economy path SSP5-85. Grey represents the study area, blue indicates suitable habitats, and pink denotes suitable habitats that are inaccessible under a dispersal limitation of 10m/yr. "1km" and "30m" represent the spatial resolutions.

#### 1.1.3.2*Chimonobambusa opienensis*

Table S11 Variable contribution and permutation importance in environmental factors affecting habitat suitability of *C. opienensis* at 30m resolution.

| Variable | Percent contribution | Permutation importance |
| --- | --- | --- |
| Bio12 Annual Precipitation | 29.88 | 44.56 |
| Spring Precipitation | 14.18 | 11.90 |
| Bio4 Temperature Seasonality | 12.65 | 21.52 |
| Bio15 Precipitation Seasonality | 11.57 | 8.91 |
| Summer Mean Temperature | 9.44 | 9.23 |
| Bio7 Temperature Annual Range | 6.31 | 1.30 |
| Agrotype | 4.52 | 0.59 |
| Slope | 3.27 | 0.30 |
| The amount of direct sunlight in summer | 2.72 | 0.17 |
| Distance to other rivers | 1.98 | 0.40 |
| The amount of direct sunlight in spring | 1.95 | 0.97 |
| Summer Sunshine Duration | 1.10 | 0.11 |
| Distance to Dry Valleys | 0.43 | 0.05 |

Table S12 Variable contribution and permutation importance in environmental factors affecting habitat suitability of *C. opienensis* at 1km resolution.

| 变量  Variable | 贡献百分比  Percent contribution | 排列重要性  Permutation importance |
| --- | --- | --- |
| Bio12 Annual Precipitation | 33.84 | 43.08 |
| Agrotype | 17.41 | 3.32 |
| Bio4 Temperature Seasonality | 16.11 | 25.00 |
| Bio15 Precipitation Seasonality | 14.32 | 8.51 |
| Spring Precipitation | 6.54 | 1.49 |
| Summer Mean Temperature | 3.67 | 10.67 |
| The amount of direct sunlight in summer | 2.80 | 1.40 |
| Bio7 Temperature Annual Range | 1.60 | 1.79 |
| The amount of direct sunlight in spring | 1.19 | 0.56 |
| Slope | 0.98 | 1.84 |
| Distance to other rivers | 0.74 | 0.40 |
| Summer Sunshine Duration | 0.68 | 1.90 |
| Distance to Dry Valleys | 0.11 | 0.05 |

Figure S7 Response of suitable habitat for *C. opienensis* to climate change and the impact of dispersal ability on its distribution at 1km and 30m resolutions. The left side shows the prediction results with a resolution of 1km, and the right side shows the prediction results with a resolution of 30m. 2000 represents the situation at the beginning of this century, “1” denotes the middle of the century (2041-2060) and “2” denotes the end of the century (2081-2100). G represents the GISS-E2-1-G model, M represents the MRI-ESM2-0 model, 26 represents the sharing economy path SSP1-26, 45 represents the sharing economy path SSP2-45, 85 represents the sharing economy path SSP5-85. Grey represents the study area, blue indicates suitable habitats, and pink denotes suitable habitats that are inaccessible under a dispersal limitation of 10m/yr. "1km" and "30m" represent the spatial resolutions.

#### 1.1.3.3*Chimonobambusa tumidissinoda*

Table S13 Variable contribution and permutation importance in environmental factors affecting habitat suitability of *C. tumidissinoda* at 30m resolution.

| Variable | Percent contribution | Permutation importance |
| --- | --- | --- |
| Bio15 Precipitation Seasonality | 16.51 | 8.80 |
| Bio7 Temperature Annual Range | 16.39 | 12.48 |
| Bio12 Annual Precipitation | 15.02 | 15.41 |
| Agrotype | 11.05 | 6.46 |
| Summer Mean Temperature | 9.61 | 7.39 |
| Bio4 Temperature Seasonality | 9.40 | 30.79 |
| Spring Precipitation | 8.17 | 10.16 |
| Slope | 5.38 | 3.14 |
| Summer Sunshine Duration | 2.24 | 1.49 |
| The amount of direct sunlight in spring | 2.13 | 1.53 |
| The amount of direct sunlight in summer | 1.97 | 0.95 |
| Distance to other rivers | 1.94 | 1.26 |
| Distance to Dry Valleys | 0.20 | 0.13 |

Table S14 Variable contribution and permutation importance in environmental factors affecting habitat suitability of *C. tumidissinoda* at 1km resolution.

| Variable | Percent contribution | Permutation importance |
| --- | --- | --- |
| Agrotype | 33.45 | 13.52 |
| Bio15 Precipitation Seasonality | 15.37 | 14.27 |
| Bio7 Temperature Annual Range | 9.19 | 8.41 |
| Bio12 Annual Precipitation | 8.67 | 4.48 |
| Bio4 Temperature Seasonality | 8.26 | 20.81 |
| Summer Mean Temperature | 6.29 | 6.09 |
| Spring Precipitation | 6.22 | 16.53 |
| Slope | 3.51 | 7.90 |
| The amount of direct sunlight in spring | 3.01 | 3.97 |
| Distance to other rivers | 2.74 | 1.46 |
| The amount of direct sunlight in summer | 1.57 | 0.40 |
| Summer Sunshine Duration | 1.43 | 1.80 |
| Distance to Dry Valleys | 0.30 | 0.37 |

Figure S8 Response of suitable habitat for *C. tumidissinoda* to climate change and the impact of dispersal ability on its distribution at 1km and 30m resolutions. The left side shows the prediction results with a resolution of 1km, and the right side shows the prediction results with a resolution of 30m. 2000 represents the situation at the beginning of this century, “1” denotes the middle of the century (2041-2060) and “2” denotes the end of the century (2081-2100). G represents the GISS-E2-1-G model, M represents the MRI-ESM2-0 model, 26 represents the sharing economy path SSP1-26, 45 represents the sharing economy path SSP2-45, 85 represents the sharing economy path SSP5-85. Grey represents the study area, blue indicates suitable habitats, and pink denotes suitable habitats that are inaccessible under a dispersal limitation of 10m/yr. "1km" and "30m" represent the spatial resolutions.

### 1.1.4*Fargesia*

#### 1.1.4.1*Fargesia denudate*

Table S15 Variable contribution and permutation importance in environmental factors affecting habitat suitability of *F. denudate* at 30m resolution.

| Variable | Percent contribution | Permutation importance |
| --- | --- | --- |
| Bio15 Precipitation Seasonality | 33.38 | 13.28 |
| Bio4 Temperature Seasonality | 20.32 | 33.46 |
| Summer Mean Temperature | 12.51 | 17.21 |
| Bio12 Annual Precipitation | 9.52 | 5.15 |
| Bio7 Temperature Annual Range | 5.53 | 12.65 |
| Distance to other rivers | 4.34 | 2.09 |
| Slope | 4.08 | 2.85 |
| Agrotype | 3.95 | 1.86 |
| Spring Precipitation | 2.30 | 8.83 |
| The amount of direct sunlight in spring | 2.17 | 1.15 |
| The amount of direct sunlight in summer | 0.96 | 0.48 |
| Summer Sunshine Duration | 0.80 | 0.42 |
| Distance to Dry Valleys | 0.14 | 0.56 |

Table S16 Variable contribution and permutation importance in environmental factors affecting habitat suitability of *F. denudate* at 1km resolution.

| Variable | Percent contribution | Permutation importance |
| --- | --- | --- |
| Bio15 Precipitation Seasonality | 39.65 | 15.02 |
| Bio4 Temperature Seasonality | 19.67 | 44.03 |
| Bio7 Temperature Annual Range | 15.41 | 9.77 |
| Summer Mean Temperature | 11.39 | 10.92 |
| The amount of direct sunlight in summer | 2.92 | 1.15 |
| Distance to other rivers | 2.41 | 1.78 |
| Summer Sunshine Duration | 2.03 | 2.17 |
| Bio12 Annual Precipitation | 1.94 | 2.27 |
| Spring Precipitation | 1.70 | 9.30 |
| Slope | 1.39 | 2.06 |
| Agrotype | 0.60 | 0.37 |
| The amount of direct sunlight in spring | 0.55 | 0.83 |
| Distance to Dry Valleys | 0.34 | 0.34 |

Figure S9 Response of suitable habitat for *F. denudate* to climate change and the impact of dispersal ability on its distribution at 1km and 30m resolutions. The left side shows the prediction results with a resolution of 1km, and the right side shows the prediction results with a resolution of 30m. 2000 represents the situation at the beginning of this century, “1” denotes the middle of the century (2041-2060) and “2” denotes the end of the century (2081-2100). G represents the GISS-E2-1-G model, M represents the MRI-ESM2-0 model, 26 represents the sharing economy path SSP1-26, 45 represents the sharing economy path SSP2-45, 85 represents the sharing economy path SSP5-85. Grey represents the study area, blue indicates suitable habitats, and pink denotes suitable habitats that are inaccessible under a dispersal limitation of 10m/yr. "1km" and "30m" represent the spatial resolutions. Grey represents the study area, blue indicates suitable habitats, and pink denotes suitable habitats that are inaccessible under a dispersal limitation of 10m/yr. "1km" and "30m" represent the spatial resolutions.

#### 1.1.4.2*Fargesia rufa*

Table S17 Variable contribution and permutation importance in environmental factors affecting habitat suitability of *F. rufa* at 30m resolution.

| Variable | Percent contribution | Permutation importance |
| --- | --- | --- |
| Summer Mean Temperature | 23.83 | 41.05 |
| Bio4 Temperature Seasonality | 18.05 | 20.23 |
| Bio12 Annual Precipitation | 15.38 | 15.85 |
| Spring Precipitation | 9.98 | 11.50 |
| Bio7 Temperature Annual Range | 9.98 | 4.52 |
| Bio15 Precipitation Seasonality | 8.76 | 0.39 |
| Slope | 4.42 | 2.04 |
| The amount of direct sunlight in summer | 3.09 | 1.78 |
| Agrotype | 2.71 | 1.15 |
| Summer Sunshine Duration | 1.50 | 0.59 |
| The amount of direct sunlight in spring | 1.28 | 0.38 |
| Distance to other rivers | 0.88 | 0.12 |
| Distance to Dry Valleys | 0.15 | 0.42 |

Table S18 Variable contribution and permutation importance in environmental factors affecting habitat suitability of *F. rufa* at 1km resolution.

| Variable | Percent contribution | Permutation importance |
| --- | --- | --- |
| Summer Mean Temperature | 23.57 | 27.87 |
| Bio4 Temperature Seasonality | 17.67 | 26.52 |
| Bio12 Annual Precipitation | 17.41 | 7.45 |
| Spring Precipitation | 11.71 | 16.27 |
| Bio15 Precipitation Seasonality | 10.62 | 4.27 |
| Bio7 Temperature Annual Range | 8.86 | 6.79 |
| Agrotype | 3.44 | 2.68 |
| The amount of direct sunlight in spring | 1.63 | 1.12 |
| Slope | 1.62 | 2.61 |
| The amount of direct sunlight in summer | 1.45 | 2.18 |
| Summer Sunshine Duration | 1.32 | 1.12 |
| Distance to other rivers | 0.53 | 0.68 |
| Distance to Dry Valleys | 0.15 | 0.43 |

Figure S10 Response of suitable habitat for *F. rufa* to climate change and the impact of dispersal ability on its distribution at 1km and 30m resolutions. The left side shows the prediction results with a resolution of 1km, and the right side shows the prediction results with a resolution of 30m. 2000 represents the situation at the beginning of this century, “1” denotes the middle of the century (2041-2060) and “2” denotes the end of the century (2081-2100). G represents the GISS-E2-1-G model, M represents the MRI-ESM2-0 model, 26 represents the sharing economy path SSP1-26, 45 represents the sharing economy path SSP2-45, 85 represents the sharing economy path SSP5-85. Grey represents the study area, blue indicates suitable habitats, and pink denotes suitable habitats that are inaccessible under a dispersal limitation of 10m/yr. "1km" and "30m" represent the spatial resolutions.

#### 1.1.4.3*Fargesia robusta*

Table S19 Variable contribution and permutation importance in environmental factors affecting habitat suitability of *F. robusta* at 30m resolution

| Variable | Percent contribution | Permutation importance |
| --- | --- | --- |
| Bio15 Precipitation Seasonality | 22.65 | 9.72 |
| Spring Precipitation | 16.99 | 44.15 |
| Summer Mean Temperature | 16.35 | 25.74 |
| Bio7 Temperature Annual Range | 14.24 | 4.98 |
| Bio4 Temperature Seasonality | 7.63 | 8.58 |
| Slope | 6.10 | 2.38 |
| Agrotype | 5.59 | 1.23 |
| Bio12 Annual Precipitation | 3.49 | 1.05 |
| Distance to other rivers | 2.73 | 0.53 |
| The amount of direct sunlight in spring | 1.54 | 0.70 |
| Summer Sunshine Duration | 1.52 | 0.36 |
| The amount of direct sunlight in summer | 0.73 | 0.23 |
| Distance to Dry Valleys | 0.43 | 0.36 |

Table S20 Variable contribution and permutation importance in environmental factors affecting habitat suitability of *F. robusta* at 1km resolution

| Variable | Percent contribution | Permutation importance |
| --- | --- | --- |
| Bio15 Precipitation Seasonality | 23.29 | 7.93 |
| Bio7 Temperature Annual Range | 18.82 | 11.10 |
| Summer Mean Temperature | 15.80 | 22.35 |
| Spring Precipitation | 13.73 | 38.42 |
| Bio4 Temperature Seasonality | 7.80 | 6.80 |
| Bio12 Annual Precipitation | 4.60 | 6.00 |
| Distance to Dry Valleys | 4.28 | 0.39 |
| Agrotype | 2.77 | 0.85 |
| Slope | 2.44 | 2.41 |
| The amount of direct sunlight in summer | 2.12 | 1.76 |
| Distance to other rivers | 1.62 | 0.36 |
| Summer Sunshine Duration | 1.45 | 0.88 |
| The amount of direct sunlight in spring | 1.28 | 0.75 |

Figure S11 Response of suitable habitat for *F. robusta* to climate change and the impact of dispersal ability on its distribution at 1km and 30m resolutions. The left side shows the prediction results with a resolution of 1km, and the right side shows the prediction results with a resolution of 30m. 2000 represents the situation at the beginning of this century, “1” denotes the middle of the century (2041-2060) and “2” denotes the end of the century (2081-2100). G represents the GISS-E2-1-G model, M represents the MRI-ESM2-0 model, 26 represents the sharing economy path SSP1-26, 45 represents the sharing economy path SSP2-45, 85 represents the sharing economy path SSP5-85. Grey represents the study area, blue indicates suitable habitats, and pink denotes suitable habitats that are inaccessible under a dispersal limitation of 10m/yr. "1km" and "30m" represent the spatial resolutions.

#### 1.1.4.4*Fargesia nitida*

Table S21 Variable contribution and permutation importance in environmental factors affecting habitat suitability of *F. nitida* at 30m resolution

| Variable | Percent contribution | Permutation importance |
| --- | --- | --- |
| Bio15 Precipitation Seasonality | 34.28 | 2.71 |
| Summer Mean Temperature | 19.53 | 49.53 |
| Agrotype | 12.42 | 5.30 |
| Bio7 Temperature Annual Range | 11.36 | 21.30 |
| Spring Precipitation | 8.30 | 8.84 |
| Distance to other rivers | 5.01 | 2.06 |
| Summer Sunshine Duration | 2.11 | 1.33 |
| Slope | 1.96 | 2.95 |
| Bio4 Temperature Seasonality | 1.34 | 2.25 |
| Bio12 Annual Precipitation | 1.24 | 0.60 |
| The amount of direct sunlight in spring | 1.10 | 0.68 |
| The amount of direct sunlight in summer | 1.05 | 1.53 |
| Distance to Dry Valleys | 0.30 | 0.92 |

Table S22 Variable contribution and permutation importance in environmental factors affecting habitat suitability of *F. nitida* at 1km resolution

| Variable | Percent contribution | Permutation importance |
| --- | --- | --- |
| Bio15 Precipitation Seasonality | 38.50 | 4.39 |
| Bio7 Temperature Annual Range | 21.96 | 29.79 |
| Summer Mean Temperature | 19.25 | 45.18 |
| Spring Precipitation | 5.05 | 9.27 |
| The amount of direct sunlight in summer | 4.09 | 1.18 |
| Distance to other rivers | 2.97 | 1.43 |
| Agrotype | 2.06 | 0.39 |
| Summer Sunshine Duration | 1.93 | 0.30 |
| Bio4 Temperature Seasonality | 1.22 | 4.02 |
| Distance to Dry Valleys | 1.10 | 1.60 |
| Bio12 Annual Precipitation | 0.87 | 1.68 |
| The amount of direct sunlight in spring | 0.52 | 0.41 |
| Slope | 0.49 | 0.35 |

Figure S12 Response of suitable habitat for *F. nitida* to climate change and the impact of dispersal ability on its distribution at 1km and 30m resolutions. The left side shows the prediction results with a resolution of 1km, and the right side shows the prediction results with a resolution of 30m. 2000 represents the situation at the beginning of this century, “1” denotes the middle of the century (2041-2060) and “2” denotes the end of the century (2081-2100). G represents the GISS-E2-1-G model, M represents the MRI-ESM2-0 model, 26 represents the sharing economy path SSP1-26, 45 represents the sharing economy path SSP2-45, 85 represents the sharing economy path SSP5-85. Grey represents the study area, blue indicates suitable habitats, and pink denotes suitable habitats that are inaccessible under a dispersal limitation of 10m/yr. "1km" and "30m" represent the spatial resolutions.

#### 1.1.4.5*Fargesia scabrida*

Table S23 Variable contribution and permutation importance in environmental factors affecting habitat suitability of *F. scabrida* at 30m resolution

| Variable | Percent contribution | Permutation importance |
| --- | --- | --- |
| Summer Mean Temperature | 20.25 | 14.79 |
| Bio12 Annual Precipitation | 16.91 | 16.19 |
| Bio7 Temperature Annual Range | 15.67 | 36.78 |
| Bio15 Precipitation Seasonality | 15.04 | 1.90 |
| Bio4 Temperature Seasonality | 13.25 | 19.36 |
| The amount of direct sunlight in spring | 3.90 | 0.96 |
| Slope | 3.89 | 1.52 |
| Distance to other rivers | 3.57 | 1.26 |
| Spring Precipitation | 3.07 | 5.02 |
| The amount of direct sunlight in summer | 1.62 | 1.02 |
| Summer Sunshine Duration | 1.42 | 0.83 |
| Agrotype | 1.29 | 0.31 |
| Distance to Dry Valleys | 0.12 | 0.05 |

Table S24 Variable contribution and permutation importance in environmental factors affecting habitat suitability of *F. scabrida* at 1km resolution

| Variable | Percent contribution | Permutation importance |
| --- | --- | --- |
| Summer Mean Temperature | 23.39 | 23.70 |
| Bio7 Temperature Annual Range | 17.30 | 17.41 |
| Bio15 Precipitation Seasonality | 16.42 | 1.25 |
| Bio4 Temperature Seasonality | 15.89 | 39.41 |
| Bio12 Annual Precipitation | 13.62 | 5.39 |
| Agrotype | 4.41 | 3.61 |
| Distance to other rivers | 3.64 | 1.50 |
| The amount of direct sunlight in summer | 1.45 | 1.47 |
| Spring Precipitation | 1.19 | 2.89 |
| Slope | 0.93 | 1.83 |
| Summer Sunshine Duration | 0.82 | 0.36 |
| The amount of direct sunlight in spring | 0.81 | 0.99 |
| Distance to Dry Valleys | 0.14 | 0.18 |

Figure S13 Response of suitable habitat for *F. scabrida* to climate change and the impact of dispersal ability on its distribution at 1km and 30m resolutions. The left side shows the prediction results with a resolution of 1km, and the right side shows the prediction results with a resolution of 30m. 2000 represents the situation at the beginning of this century, “1” denotes the middle of the century (2041-2060) and “2” denotes the end of the century (2081-2100). G represents the GISS-E2-1-G model, M represents the MRI-ESM2-0 model, 26 represents the sharing economy path SSP1-26, 45 represents the sharing economy path SSP2-45, 85 represents the sharing economy path SSP5-85. Grey represents the study area, blue indicates suitable habitats, and pink denotes suitable habitats that are inaccessible under a dispersal limitation of 10m/yr. "1km" and "30m" represent the spatial resolutions.

#### 1.1.4.6*Fargesia ferax*

Table S25 Variable contribution and permutation importance in environmental factors affecting habitat suitability of *F. ferax* at 30m resolution.

| Variable | Percent contribution | Permutation importance |
| --- | --- | --- |
| Summer Mean Temperature | 18.573025 | 22.9907 |
| Bio4 Temperature Seasonality | 15.225425 | 7.157775 |
| Spring Precipitation | 15.0618 | 4.028075 |
| Bio15 Precipitation Seasonality | 11.6902 | 20.560875 |
| Bio7 Temperature Annual Range | 10.44385 | 17.1349 |
| Agrotype | 9.40675 | 5.258025 |
| Distance to other rivers | 7.5575 | 1.721325 |
| The amount of direct sunlight in summer | 3.4082 | 6.15945 |
| Bio12 Annual Precipitation | 3.347425 | 10.35725 |
| Slope | 2.32365 | 1.76935 |
| The amount of direct sunlight in spring | 1.57395 | 0.972625 |
| Summer Sunshine Duration | 0.749775 | 0.6728 |
| Distance to Dry Valleys | 0.63835 | 1.216875 |

Table S26 Variable contribution and permutation importance in environmental factors affecting habitat suitability of *F. ferax* at 1km resolution.

| Variable | Percent contribution | Permutation importance |
| --- | --- | --- |
| Summer Mean Temperature | 17.19 | 18.92 |
| Bio4 Temperature Seasonality | 14.46 | 3.97 |
| Spring Precipitation | 14.13 | 8.49 |
| Bio15 Precipitation Seasonality | 12.93 | 23.69 |
| Bio7 Temperature Annual Range | 12.74 | 7.36 |
| Agrotype | 11.10 | 7.11 |
| Summer Sunshine Duration | 9.21 | 16.74 |
| Distance to other rivers | 3.31 | 2.19 |
| Bio12 Annual Precipitation | 1.45 | 4.21 |
| Slope | 1.26 | 0.93 |
| Distance to Dry Valleys | 0.97 | 3.09 |
| The amount of direct sunlight in spring | 0.82 | 0.23 |
| The amount of direct sunlight in summer | 0.43 | 3.06 |

Figure S14 Response of suitable habitat for *F. ferax* to climate change and the impact of dispersal ability on its distribution at 1km and 30m resolutions. The left side shows the prediction results with a resolution of 1km, and the right side shows the prediction results with a resolution of 30m. 2000 represents the situation at the beginning of this century, “1” denotes the middle of the century (2041-2060) and “2” denotes the end of the century (2081-2100). G represents the GISS-E2-1-G model, M represents the MRI-ESM2-0 model, 26 represents the sharing economy path SSP1-26, 45 represents the sharing economy path SSP2-45, 85 represents the sharing economy path SSP5-85. Grey represents the study area, blue indicates suitable habitats, and pink denotes suitable habitats that are inaccessible under a dispersal limitation of 10m/yr. "1km" and "30m" represent the spatial resolutions.

#### 1.1.4.7*Fargesia obliqua*

Table S27 Variable contribution and permutation importance in environmental factors affecting habitat suitability of *F. obliqua* at 30m resolution.

| Variable | Percent contribution | Permutation importance |
| --- | --- | --- |
| Bio15 Precipitation Seasonality | 44.94 | 41.90 |
| Summer Mean Temperature | 16.44 | 20.00 |
| Bio7 Temperature Annual Range | 10.57 | 2.49 |
| Bio4 Temperature Seasonality | 6.93 | 9.83 |
| Slope | 3.73 | 2.53 |
| Spring Precipitation | 3.49 | 4.52 |
| Agrotype | 3.13 | 0.38 |
| Distance to other rivers | 2.67 | 0.56 |
| Bio12 Annual Precipitation | 2.55 | 10.01 |
| The amount of direct sunlight in spring | 2.13 | 1.44 |
| Summer Sunshine Duration | 1.58 | 0.92 |
| The amount of direct sunlight in summer | 1.01 | 2.87 |
| Distance to Dry Valleys | 0.84 | 2.54 |

Table S28 Variable contribution and permutation importance in environmental factors affecting habitat suitability of *F. obliqua* at 1km resolution.

| Variable | Percent contribution | Permutation importance |
| --- | --- | --- |
| Bio15 Precipitation Seasonality | 51.88 | 31.63 |
| Summer Mean Temperature | 13.59 | 11.41 |
| Bio7 Temperature Annual Range | 12.98 | 3.86 |
| Agrotype | 7.58 | 4.13 |
| The amount of direct sunlight in summer | 4.74 | 1.43 |
| Bio4 Temperature Seasonality | 2.39 | 2.68 |
| Bio12 Annual Precipitation | 2.12 | 33.65 |
| Distance to Dry Valleys | 1.47 | 10.32 |
| The amount of direct sunlight in spring | 0.88 | 0.18 |
| Summer Sunshine Duration | 0.73 | 0.14 |
| Spring Precipitation | 0.70 | 0.24 |
| Distance to other rivers | 0.54 | 0.16 |
| Slope | 0.39 | 0.16 |

Figure S15 Response of suitable habitat for *F. obliqua* to climate change and the impact of dispersal ability on its distribution at 1km and 30m resolutions. The left side shows the prediction results with a resolution of 1km, and the right side shows the prediction results with a resolution of 30m. 2000 represents the situation at the beginning of this century, “1” denotes the middle of the century (2041-2060) and “2” denotes the end of the century (2081-2100). G represents the GISS-E2-1-G model, M represents the MRI-ESM2-0 model, 26 represents the sharing economy path SSP1-26, 45 represents the sharing economy path SSP2-45, 85 represents the sharing economy path SSP5-85. Grey represents the study area, blue indicates suitable habitats, and pink denotes suitable habitats that are inaccessible under a dispersal limitation of 10m/yr. "1km" and "30m" represent the spatial resolutions.

### 1.1.5*Yushania*

#### 1.1.5.1*Yushania brevipaniculata*

Table S29 Variable contribution and permutation importance in environmental factors affecting habitat suitability of *Y. brevipaniculata* at 30m resolution

| Variable | Percent contribution | Permutation importance |
| --- | --- | --- |
| Summer Mean Temperature | 22.26 | 15.00 |
| Bio7 Temperature Annual Range | 14.24 | 24.59 |
| Bio4 Temperature Seasonality | 14.14 | 25.49 |
| Bio15 Precipitation Seasonality | 14.13 | 2.38 |
| Bio12 Annual Precipitation | 13.12 | 9.45 |
| Spring Precipitation | 9.51 | 19.09 |
| Slope | 4.70 | 1.57 |
| Agrotype | 2.95 | 0.99 |
| Distance to other rivers | 1.84 | 0.12 |
| Summer Sunshine Duration | 1.14 | 0.25 |
| Distance to Dry Valleys | 0.89 | 0.51 |
| The amount of direct sunlight in spring | 0.55 | 0.12 |
| The amount of direct sunlight in summer | 0.52 | 0.45 |

Table S30 Variable contribution and permutation importance in environmental factors affecting habitat suitability of *Y. brevipaniculata* at 1km resolution

| Variable | Percent contribution | Permutation importance |
| --- | --- | --- |
| Summer Mean Temperature | 22.93 | 17.18 |
| Bio15 Precipitation Seasonality | 21.08 | 7.21 |
| Bio4 Temperature Seasonality | 15.97 | 28.63 |
| Bio7 Temperature Annual Range | 12.36 | 18.81 |
| Agrotype | 9.68 | 5.25 |
| Bio12 Annual Precipitation | 7.09 | 5.62 |
| Spring Precipitation | 3.68 | 11.06 |
| Summer Sunshine Duration | 1.52 | 1.95 |
| Distance to other rivers | 1.46 | 0.57 |
| Slope | 1.41 | 1.38 |
| The amount of direct sunlight in spring | 1.30 | 1.14 |
| The amount of direct sunlight in summer | 1.06 | 1.10 |
| Distance to Dry Valleys | 0.46 | 0.10 |

Figure S16 Response of suitable habitat for *Y. brevipaniculata* to climate change and the impact of dispersal ability on its distribution at 1km and 30m resolutions. The left side shows the prediction results with a resolution of 1km, and the right side shows the prediction results with a resolution of 30m. 2000 represents the situation at the beginning of this century, “1” denotes the middle of the century (2041-2060) and “2” denotes the end of the century (2081-2100). G represents the GISS-E2-1-G model, M represents the MRI-ESM2-0 model, 26 represents the sharing economy path SSP1-26, 45 represents the sharing economy path SSP2-45, 85 represents the sharing economy path SSP5-85. Grey represents the study area, blue indicates suitable habitats, and pink denotes suitable habitats that are inaccessible under a dispersal limitation of 10m/yr. "1km" and "30m" represent the spatial resolutions.

#### 1.1.5.2*Yushania lineolate*

Table S31 Variable contribution and permutation importance in environmental factors affecting habitat suitability of *Y. lineolate* at 30m resolution.

| Variable | Percent contribution | Permutation importance |
| --- | --- | --- |
| Bio7 Temperature Annual Range | 32.92 | 30.97 |
| Bio4 Temperature Seasonality | 16.77 | 12.13 |
| Bio15 Precipitation Seasonality | 13.74 | 13.37 |
| Summer Mean Temperature | 9.71 | 8.23 |
| Agrotype | 8.78 | 6.12 |
| Bio12 Annual Precipitation | 6.04 | 8.84 |
| Slope | 4.36 | 9.05 |
| Distance to other rivers | 2.55 | 1.21 |
| Spring Precipitation | 1.34 | 2.94 |
| The amount of direct sunlight in spring | 1.22 | 2.37 |
| Summer Sunshine Duration | 1.16 | 1.22 |
| The amount of direct sunlight in summer | 1.10 | 2.09 |
| Distance to Dry Valleys | 0.32 | 1.46 |

Table S32 Variable contribution and permutation importance in environmental factors affecting habitat suitability of *Y. lineolate* at 1km resolution.

| Variable | Percent contribution | Permutation importance |
| --- | --- | --- |
| Bio7 Temperature Annual Range | 37.05 | 13.67 |
| Bio4 Temperature Seasonality | 18.56 | 14.89 |
| Summer Mean Temperature | 14.06 | 16.16 |
| Bio15 Precipitation Seasonality | 10.64 | 8.63 |
| Bio12 Annual Precipitation | 6.31 | 22.74 |
| Agrotype | 5.61 | 6.35 |
| Distance to other rivers | 1.79 | 1.28 |
| Slope | 1.56 | 3.37 |
| Summer Sunshine Duration | 1.23 | 2.39 |
| The amount of direct sunlight in summer | 1.11 | 5.56 |
| Spring Precipitation | 1.01 | 3.14 |
| The amount of direct sunlight in spring | 0.79 | 1.21 |
| Distance to Dry Valleys | 0.29 | 0.62 |

Figure S17 Response of suitable habitat for *Y. lineolate* to climate change and the impact of dispersal ability on its distribution at 1km and 30m resolutions. The left side shows the prediction results with a resolution of 1km, and the right side shows the prediction results with a resolution of 30m. 2000 represents the situation at the beginning of this century, “1” denotes the middle of the century (2041-2060) and “2” denotes the end of the century (2081-2100). G represents the GISS-E2-1-G model, M represents the MRI-ESM2-0 model, 26 represents the sharing economy path SSP1-26, 45 represents the sharing economy path SSP2-45, 85 represents the sharing economy path SSP5-85. Grey represents the study area, blue indicates suitable habitats, and pink denotes suitable habitats that are inaccessible under a dispersal limitation of 10m/yr. "1km" and "30m" represent the spatial resolutions.

#### 1.1.5.3*Yushania maculate*

Table S33 Variable contribution and permutation importance in environmental factors affecting habitat suitability of *Y. maculate* at 30m resolution.

| Variable | Percent contribution | Permutation importance |
| --- | --- | --- |
| Bio7 Temperature Annual Range | 36.00 | 16.30 |
| Summer Mean Temperature | 11.64 | 41.20 |
| Bio4 Temperature Seasonality | 9.30 | 3.70 |
| Agrotype | 9.17 | 5.43 |
| Bio12 Annual Precipitation | 8.86 | 10.46 |
| Bio15 Precipitation Seasonality | 8.16 | 6.82 |
| Spring Precipitation | 4.98 | 9.20 |
| Slope | 3.65 | 2.61 |
| The amount of direct sunlight in summer | 3.14 | 2.20 |
| Distance to other rivers | 3.06 | 1.20 |
| The amount of direct sunlight in spring | 1.27 | 0.64 |
| Summer Sunshine Duration | 0.75 | 0.21 |
| Distance to Dry Valleys | 0.01 | 0.02 |

Table S34 Variable contribution and permutation importance in environmental factors affecting habitat suitability of *Y. maculate* at 1km resolution.

| Variable | Percent contribution | Permutation importance |
| --- | --- | --- |
| Bio7 Temperature Annual Range | 46.12 | 7.24 |
| Summer Mean Temperature | 10.53 | 27.09 |
| Bio15 Precipitation Seasonality | 8.31 | 2.83 |
| Bio4 Temperature Seasonality | 7.90 | 1.80 |
| Agrotype | 7.29 | 6.17 |
| Bio12 Annual Precipitation | 7.17 | 27.82 |
| Spring Precipitation | 6.66 | 19.88 |
| The amount of direct sunlight in summer | 2.00 | 2.39 |
| Slope | 1.61 | 2.41 |
| Distance to other rivers | 1.09 | 1.02 |
| The amount of direct sunlight in spring | 0.76 | 0.42 |
| Summer Sunshine Duration | 0.56 | 0.93 |
| Distance to Dry Valleys | 0 | 0 |

Figure S18 Response of suitable habitat for *Y. maculate* to climate change and the impact of dispersal ability on its distribution at 1km and 30m resolutions. The left side shows the prediction results with a resolution of 1km, and the right side shows the prediction results with a resolution of 30m. 2000 represents the situation at the beginning of this century, “1” denotes the middle of the century (2041-2060) and “2” denotes the end of the century (2081-2100). G represents the GISS-E2-1-G model, M represents the MRI-ESM2-0 model, 26 represents the sharing economy path SSP1-26, 45 represents the sharing economy path SSP2-45, 85 represents the sharing economy path SSP5-85. Grey represents the study area, blue indicates suitable habitats, and pink denotes suitable habitats that are inaccessible under a dispersal limitation of 10m/yr. "1km" and "30m" represent the spatial resolutions.

#### 1.1.5.4*Yushania glauca*

Table S35 Variable contribution and permutation importance in environmental factors affecting habitat suitability of *Y. glauca* at 30m resolution.

| Variable | Percent contribution | Permutation importance |
| --- | --- | --- |
| Bio7 Temperature Annual Range | 20.89 | 1.61 |
| Bio15 Precipitation Seasonality | 17.57 | 8.33 |
| Bio12 Annual Precipitation | 16.65 | 30.14 |
| Summer Mean Temperature | 15.37 | 25.91 |
| Spring Precipitation | 12.09 | 15.78 |
| Bio4 Temperature Seasonality | 6.53 | 5.02 |
| Agrotype | 5.01 | 5.05 |
| Slope | 1.82 | 1.77 |
| The amount of direct sunlight in summer | 1.51 | 1.87 |
| Distance to other rivers | 1.19 | 2.71 |
| Summer Sunshine Duration | 0.84 | 1.11 |
| The amount of direct sunlight in spring | 0.50 | 0.66 |
| Distance to Dry Valleys | 0.04 | 0.05 |

Table S36 Variable contribution and permutation importance in environmental factors affecting habitat suitability of *Y. glauca* at 1km resolution.

| Variable | Percent contribution | Permutation importance |
| --- | --- | --- |
| Bio7 Temperature Annual Range | 22.27 | 0.76 |
| Agrotype | 19.47 | 1.51 |
| Bio15 Precipitation Seasonality | 17.02 | 2.33 |
| Bio12 Annual Precipitation | 13.76 | 44.74 |
| Summer Mean Temperature | 11.07 | 44.08 |
| Spring Precipitation | 5.53 | 2.71 |
| Bio4 Temperature Seasonality | 5.08 | 0.39 |
| The amount of direct sunlight in summer | 1.90 | 0.80 |
| Slope | 1.36 | 1.34 |
| Summer Sunshine Duration | 1.19 | 0.85 |
| Distance to other rivers | 1.01 | 0.37 |
| The amount of direct sunlight in spring | 0.31 | 0.12 |
| Distance to Dry Valleys | 0.04 | 0 |

Figure S19 Response of suitable habitat for *Y. glauca* to climate change and the impact of dispersal ability on its distribution at 1km and 30m resolutions. The left side shows the prediction results with a resolution of 1km, and the right side shows the prediction results with a resolution of 30m. 2000 represents the situation at the beginning of this century, “1” denotes the middle of the century (2041-2060) and “2” denotes the end of the century (2081-2100). G represents the GISS-E2-1-G model, M represents the MRI-ESM2-0 model, 26 represents the sharing economy path SSP1-26, 45 represents the sharing economy path SSP2-45, 85 represents the sharing economy path SSP5-85. Grey represents the study area, blue indicates suitable habitats, and pink denotes suitable habitats that are inaccessible under a dispersal limitation of 10m/yr. "1km" and "30m" represent the spatial resolutions.

#### 1.1.5.5*Yushania ailuropodine*

Table S37 Variable contribution and permutation importance in environmental factors affecting habitat suitability of *Y. ailuropodine* at 1km resolution.

| Variable | Percent contribution | Permutation importance |
| --- | --- | --- |
| Bio12 Annual Precipitation | 18.58 | 15.20 |
| Bio15 Precipitation Seasonality | 17.03 | 15.76 |
| Bio7 Temperature Annual Range | 13.57 | 3.33 |
| Spring Precipitation | 11.93 | 1.88 |
| Summer Mean Temperature | 10.58 | 49.02 |
| Bio4 Temperature Seasonality | 6.82 | 4.66 |
| Agrotype | 5.76 | 3.62 |
| Summer Sunshine Duration | 4.08 | 1.66 |
| Slope | 3.80 | 0.43 |
| The amount of direct sunlight in spring | 3.01 | 1.49 |
| The amount of direct sunlight in summer | 2.46 | 0.96 |
| Distance to other rivers | 2.36 | 2.00 |
| Distance to Dry Valleys | 0.03 | 0 |

Table S38 Variable contribution and permutation importance in environmental factors affecting habitat suitability of *Y. ailuropodine* at 1km resolution.

| Variable | Percent contribution | Permutation importance |
| --- | --- | --- |
| Agrotype | 24.12 | 9.52 |
| Bio15 Precipitation Seasonality | 20.64 | 6.68 |
| Bio12 Annual Precipitation | 18.24 | 45.64 |
| Bio7 Temperature Annual Range | 15.20 | 6.94 |
| Spring Precipitation | 12.45 | 22.05 |
| Summer Mean Temperature | 5.21 | 3.06 |
| Bio4 Temperature Seasonality | 1.43 | 5.32 |
| The amount of direct sunlight in spring | 0.99 | 0.13 |
| Distance to other rivers | 0.96 | 0.41 |
| Slope | 0.42 | 0.08 |
| Distance to Dry Valleys | 0.21 | 0.12 |
| Summer Sunshine Duration | 0.13 | 0.02 |
| The amount of direct sunlight in summer | 0.00 | 0.04 |

Figure S20 Response of suitable habitat for *Y. ailuropodine* to climate change and the impact of dispersal ability on its distribution at 1km and 30m resolutions. The left side shows the prediction results with a resolution of 1km, and the right side shows the prediction results with a resolution of 30m. 2000 represents the situation at the beginning of this century, “1” denotes the middle of the century (2041-2060) and “2” denotes the end of the century (2081-2100). G represents the GISS-E2-1-G model, M represents the MRI-ESM2-0 model, 26 represents the sharing economy path SSP1-26, 45 represents the sharing economy path SSP2-45, 85 represents the sharing economy path SSP5-85. Grey represents the study area, blue indicates suitable habitats, and pink denotes suitable habitats that are inaccessible under a dispersal limitation of 10m/yr. "1km" and "30m" represent the spatial resolutions.

#### 1.1.5.6*Yushania mabianensis*

Table S39 Variable contribution and permutation importance in environmental factors affecting habitat suitability of *Y. mabianensis* at 30m resolution.

| Variable | Percent contribution | Permutation importance |
| --- | --- | --- |
| Bio15 Precipitation Seasonality | 25.21 | 27.34 |
| Bio12 Annual Precipitation | 22.41 | 5.75 |
| Bio7 Temperature Annual Range | 17.72 | 21.41 |
| Agrotype | 11.20 | 11.97 |
| Spring Precipitation | 7.88 | 10.94 |
| Summer Mean Temperature | 5.38 | 18.50 |
| Bio4 Temperature Seasonality | 4.65 | 0.43 |
| The amount of direct sunlight in spring | 1.96 | 1.02 |
| Slope | 1.49 | 1.45 |
| Distance to other rivers | 1.16 | 0.23 |
| Summer Sunshine Duration | 0.42 | 0.57 |
| Distance to Dry Valleys | 0.41 | 0.08 |
| The amount of direct sunlight in summer | 0.10 | 0.31 |

Table S40 Variable contribution and permutation importance in environmental factors affecting habitat suitability of *Y. mabianensis* at 1km resolution.

| Variable | Percent contribution | Permutation importance |
| --- | --- | --- |
| Agrotype | 24.97 | 16.39 |
| Bio15 Precipitation Seasonality | 18.25 | 13.50 |
| Bio12 Annual Precipitation | 17.41 | 7.94 |
| Bio7 Temperature Annual Range | 16.12 | 47.29 |
| Summer Mean Temperature | 7.82 | 4.10 |
| Bio4 Temperature Seasonality | 3.43 | 0.57 |
| Distance to other rivers | 3.28 | 1.64 |
| Spring Precipitation | 3.26 | 3.64 |
| Summer Sunshine Duration | 2.32 | 2.10 |
| Slope | 1.72 | 2.35 |
| The amount of direct sunlight in summer | 0.59 | 0.25 |
| Distance to Dry Valleys | 0.44 | 0.03 |
| The amount of direct sunlight in spring | 0.39 | 0.21 |

Figure S21 Response of suitable habitat for *Y. mabianensis* to climate change and the impact of dispersal ability on its distribution at 1km and 30m resolutions. The left side shows the prediction results with a resolution of 1km, and the right side shows the prediction results with a resolution of 30m. 2000 represents the situation at the beginning of this century, “1” denotes the middle of the century (2041-2060) and “2” denotes the end of the century (2081-2100). G represents the GISS-E2-1-G model, M represents the MRI-ESM2-0 model, 26 represents the sharing economy path SSP1-26, 45 represents the sharing economy path SSP2-45, 85 represents the sharing economy path SSP5-85. Grey represents the study area, blue indicates suitable habitats, and pink denotes suitable habitats that are inaccessible under a dispersal limitation of 10m/yr. "1km" and "30m" represent the spatial resolutions.

#### 1.1.5.7*Yushania dafengdingensis*

Table S41 Variable contribution and permutation importance in environmental factors affecting habitat suitability of *Y. dafengdingensis* at 30m resolution.

| Variable | Percent contribution | Permutation importance |
| --- | --- | --- |
| Bio7 Temperature Annual Range | 32.70 | 40.01 |
| Bio15 Precipitation Seasonality | 20.00 | 16.73 |
| Spring Precipitation | 10.10 | 8.76 |
| Bio12 Annual Precipitation | 8.80 | 3.62 |
| Distance to other rivers | 5.84 | 0.56 |
| Agrotype | 5.35 | 3.10 |
| Summer Mean Temperature | 5.00 | 12.07 |
| Bio4 Temperature Seasonality | 4.58 | 4.62 |
| The amount of direct sunlight in summer | 2.44 | 1.40 |
| Slope | 2.04 | 4.06 |
| Summer Sunshine Duration | 1.52 | 0.09 |
| The amount of direct sunlight in spring | 1.24 | 2.55 |
| Distance to Dry Valleys | 0.39 | 2.43 |

Table S42 Variable contribution and permutation importance in environmental factors affecting habitat suitability of *Y. dafengdingensis* at 1km resolution.

| Variable | Percent contribution | Permutation importance |
| --- | --- | --- |
| Bio7 Temperature Annual Range | 35.65 | 65.27 |
| Bio15 Precipitation Seasonality | 25.76 | 27.50 |
| Bio12 Annual Precipitation | 12.04 | 1.46 |
| Agrotype | 10.02 | 1.38 |
| Spring Precipitation | 4.67 | 3.24 |
| Distance to other rivers | 3.28 | 0.20 |
| Summer Mean Temperature | 3.13 | 0.13 |
| Bio4 Temperature Seasonality | 1.95 | 0 |
| Summer Sunshine Duration | 1.14 | 0.68 |
| Slope | 0.84 | 0.01 |
| Distance to Dry Valleys | 0.59 | 0.01 |
| The amount of direct sunlight in spring | 0.58 | 0.12 |
| The amount of direct sunlight in summer | 0.36 | 0 |

Figure S22 Response of suitable habitat for *Y. dafengdingensis* to climate change and the impact of dispersal ability on its distribution at 1km and 30m resolutions. The left side shows the prediction results with a resolution of 1km, and the right side shows the prediction results with a resolution of 30m. 2000 represents the situation at the beginning of this century, “1” denotes the middle of the century (2041-2060) and “2” denotes the end of the century (2081-2100). G represents the GISS-E2-1-G model, M represents the MRI-ESM2-0 model, 26 represents the sharing economy path SSP1-26, 45 represents the sharing economy path SSP2-45, 85 represents the sharing economy path SSP5-85. Grey represents the study area, blue indicates suitable habitats, and pink denotes suitable habitats that are inaccessible under a dispersal limitation of 10m/yr. "1km" and "30m" represent the spatial resolutions.

Table S43 Monthly average temperature data verification from 1970 – 2000

| Month | ZD&JZ1km | | | ZD&JCD30m | | | JZ1km&JCD30m | | |
| --- | --- | --- | --- | --- | --- | --- | --- | --- | --- |
|  | PCC | RMSE | MAE | PCC | RMSE | MAE | PCC | RMSE | MAE |
| 1 | 0.947 | 1.245 | 0.904 | 0.950 | 5.360 | 5.235 | 0.998 | 5.618 | 5.614 |
| 2 | 0.932 | 1.367 | 1.005 | 0.936 | 7.232 | 7.119 | 0.998 | 7.531 | 7.528 |
| 3 | 0.933 | 1.301 | 0.980 | 0.934 | 12.305 | 12.236 | 0.998 | 12.171 | 12.169 |
| 4 | 0.950 | 1.215 | 0.958 | 0.952 | 16.945 | 16.904 | 0.999 | 16.733 | 16.731 |
| 5 | 0.962 | 1.096 | 0.833 | 0.963 | 19.749 | 19.719 | 0.999 | 19.757 | 19.756 |
| 6 | 0.967 | 1.006 | 0.737 | 0.969 | 21.671 | 21.649 | 0.999 | 21.795 | 21.794 |
| 7 | 0.966 | 1.028 | 0.736 | 0.968 | 23.163 | 23.142 | 0.999 | 23.390 | 23.390 |
| 8 | 0.966 | 1.004 | 0.813 | 0.968 | 23.315 | 23.294 | 0.999 | 23.267 | 23.267 |
| 9 | 0.956 | 1.161 | 0.827 | 0.958 | 19.557 | 19.527 | 0.999 | 19.900 | 19.900 |
| 10 | 0.956 | 1.110 | 0.846 | 0.958 | 15.601 | 15.564 | 0.999 | 15.731 | 15.730 |
| 11 | 0.960 | 1.196 | 0.862 | 0.962 | 10.837 | 10.785 | 0.999 | 11.251 | 11.250 |
| 12 | 0.949 | 1.180 | 1.000 | 0.951 | 7.016 | 6.918 | 0.999 | 6.990 | 6.987 |

Note: ZD – Monthly mean temperature data measured at 42 stations in the study area; JZ1km – 1 km monthly mean temperature dataset for China (1901–2022) (Peng, 2019); JCD30m – Downscaled monthly mean temperature data; PCC – Pearson correlation coefficient; RMSE – Root mean squared error; MAE – Mean absolute error.

Table S44 Monthly average precipitation data verification from 1970 – 2000

| Month | JZ1km&JCD30m | | |
| --- | --- | --- | --- |
|  | PCC | PCC | PCC |
| 1 | 0.999 | 11.371 | 11.370 |
| 2 | 1.000 | 15.190 | 15.189 |
| 3 | 0.999 | 24.213 | 24.211 |
| 4 | 1.000 | 53.496 | 53.495 |
| 5 | 0.999 | 99.537 | 99.535 |
| 6 | 0.999 | 161.432 | 161.428 |
| 7 | 0.999 | 213.627 | 213.603 |
| 8 | 0.999 | 214.049 | 214.016 |
| 9 | 0.999 | 136.019 | 136.015 |
| 10 | 1.000 | 72.527 | 72.526 |
| 11 | 1.000 | 29.586 | 29.585 |
| 12 | 0.999 | 12.561 | 12.559 |

Note: JZ1km – 1 km monthly mean precipitation dataset for China (1901–2022) (Peng, 2020); all others as above.

| Month | JZ1km&JCD30m 2041 – 2060 | | | | | | JZ1km&JCD30m 2081 – 2100 | | | | | |
| --- | --- | --- | --- | --- | --- | --- | --- | --- | --- | --- | --- | --- |
|  | Temperature | | | Precipitation | | | Temperature | | | Precipitation | | |
|  | PCC | RMSE | MAE | PCC | RMSE | MAE | PCC | RMSE | MAE | PCC | RMSE | MAE |
| 1 | 0.961 | 68.980 | 64.902 | 0.996 | 123.106 | 107.573 | 0.971 | 58.230 | 54.795 | 0.993 | 134.195 | 116.665 |
| 2 | 0.964 | 88.046 | 83.429 | 0.993 | 185.928 | 166.296 | 0.965 | 80.591 | 76.170 | 0.995 | 165.891 | 149.621 |
| 3 | 0.959 | 115.845 | 110.562 | 0.996 | 295.207 | 279.784 | 0.962 | 112.977 | 107.725 | 0.989 | 630.806 | 615.493 |
| 4 | 0.974 | 159.393 | 155.334 | 0.958 | 518.442 | 505.800 | 0.975 | 150.450 | 146.379 | 0.986 | 630.806 | 615.493 |
| 5 | 0.982 | 181.628 | 177.979 | 0.987 | 1043.985 | 1020.715 | 0.983 | 178.049 | 174.329 | 0.987 | 957.949 | 946.580 |
| 6 | 0.993 | 206.621 | 203.424 | 0.988 | 1430.448 | 1399.940 | 0.992 | 209.408 | 206.017 | 0.991 | 1385.465 | 1348.220 |
| 7 | 0.993 | 223.155 | 220.395 | 0.953 | 3166.892 | 3009.834 | 0.994 | 221.146 | 218.236 | 0.953 | 2845.056 | 2687.240 |
| 8 | 0.994 | 228.687 | 225.759 | 0.987 | 2339.291 | 2127.050 | 0.994 | 218.913 | 215.790 | 0.989 | 1843.122 | 1709.584 |
| 9 | 0.990 | 191.833 | 188.973 | 0.969 | 1857.464 | 1832.361 | 0.991 | 185.101 | 182.040 | 0.961 | 1676.859 | 1646.270 |
| 10 | 0.987 | 146.962 | 143.424 | 0.972 | 755.492 | 737.634 | 0.985 | 141.513 | 137.712 | 0.939 | 722.036 | 710.079 |
| 11 | 0.978 | 113.042 | 108.159 | 0.980 | 307.937 | 284.827 | 0.983 | 105.946 | 101.191 | 0.986 | 320.914 | 295.705 |
| 12 | 0.978 | 67.332 | 63.581 | 0.986 | 146.479 | 126.336 | 0.973 | 64.669 | 60.787 | 0.997 | 138.693 | 118.231 |

Table S45 Monthly average temperature and monthly precipitation data verification for 2041 – 2060, 2081 – 2100 （Model M SSP1-26）

Table S46 Monthly average temperature and monthly precipitation data verification for 2041 – 2060, 2081 – 2100 （Model M SSP2-45）

| Month | JZ1km&JCD30m 2041 – 2060 | | | | | | JZ1km&JCD30m 2081 – 2100 | | | | | |
| --- | --- | --- | --- | --- | --- | --- | --- | --- | --- | --- | --- | --- |
|  | Temperature | | | Precipitation | | | Temperature | | | Precipitation | | |
|  | PCC | RMSE | MAE | PCC | RMSE | MAE | PCC | RMSE | MAE | PCC | RMSE | MAE |
| 1 | 0.967 | 69.471 | 65.989 | 0.997 | 102.858 | 89.541 | 0.969 | 69.218 | 65.557 | 0.994 | 116.009 | 101.124 |
| 2 | 0.958 | 78.197 | 74.430 | 0.997 | 150.856 | 137.048 | 0.958 | 98.988 | 93.971 | 0.996 | 150.620 | 137.093 |
| 3 | 0.954 | 115.768 | 110.674 | 0.997 | 287.996 | 273.088 | 0.955 | 124.537 | 119.617 | 0.998 | 282.177 | 266.921 |
| 4 | 0.974 | 160.819 | 156.944 | 0.985 | 541.608 | 528.156 | 0.972 | 164.513 | 160.803 | 0.986 | 523.930 | 512.577 |
| 5 | 0.983 | 187.849 | 184.224 | 0.980 | 934.657 | 921.492 | 0.983 | 192.491 | 189.077 | 0.995 | 1158.141 | 1137.234 |
| 6 | 0.991 | 213.804 | 210.314 | 0.957 | 1030.986 | 995.348 | 0.992 | 223.189 | 220.092 | 0.995 | 1443.503 | 1397.148 |
| 7 | 0.993 | 231.311 | 228.356 | 0.952 | 2110.673 | 2028.858 | 0.993 | 231.273 | 228.610 | 0.984 | 2946.831 | 2800.932 |
| 8 | 0.994 | 232.296 | 229.229 | 0.995 | 2338.152 | 2144.201 | 0.994 | 239.122 | 236.181 | 0.995 | 2182.084 | 1985.418 |
| 9 | 0.991 | 192.893 | 189.941 | 0.950 | 1643.113 | 1618.595 | 0.990 | 199.107 | 196.170 | 0.972 | 1703.494 | 1680.432 |
| 10 | 0.985 | 149.854 | 146.209 | 0.942 | 502.818 | 490.701 | 0.985 | 157.735 | 154.331 | 0.988 | 684.651 | 669.986 |
| 11 | 0.981 | 111.853 | 107.183 | 0.979 | 215.558 | 199.988 | 0.982 | 112.805 | 108.247 | 0.998 | 259.306 | 237.914 |
| 12 | 0.978 | 67.708 | 64.553 | 0.998 | 124.013 | 106.070 | 0.976 | 73.962 | 70.546 | 0.998 | 132.160 | 113.346 |

Table S47 Monthly average temperature and monthly precipitation data verification for 2041 – 2060, 2081 – 2100 (Model M SSP5-85)

| Month | JZ1km&JCD30m 2041 – 2060 | | | | | | JZ1km&JCD30m 2081 – 2100 | | | | | |
| --- | --- | --- | --- | --- | --- | --- | --- | --- | --- | --- | --- | --- |
|  | Temperature | | | Precipitation | | | Temperature | | | Precipitation | | |
|  | PCC | RMSE | MAE | PCC | RMSE | MAE | PCC | RMSE | MAE | PCC | RMSE | MAE |
| 1 | 0.970 | 76.722 | 73.239 | 0.996 | 106.275 | 91.089 | 0.972 | 86.856 | 83.236 | 0.995 | 123.625 | 108.052 |
| 2 | 0.959 | 94.509 | 89.721 | 0.997 | 129.107 | 117.407 | 0.975 | 112.163 | 107.088 | 0.992 | 162.209 | 146.605 |
| 3 | 0.953 | 127.890 | 123.321 | 0.993 | 296.874 | 282.227 | 0.956 | 146.433 | 142.506 | 0.989 | 319.785 | 305.069 |
| 4 | 0.974 | 163.011 | 159.430 | 0.980 | 504.806 | 493.212 | 0.912 | 193.140 | 190.288 | 0.923 | 597.428 | 581.414 |
| 5 | 0.982 | 187.674 | 184.317 | 0.988 | 1036.509 | 1016.876 | 0.927 | 219.659 | 216.587 | 0.981 | 1044.281 | 1032.765 |
| 6 | 0.992 | 218.252 | 215.033 | 0.980 | 1402.875 | 1367.635 | 0.915 | 253.449 | 250.442 | 0.976 | 1326.238 | 1297.926 |
| 7 | 0.993 | 234.148 | 231.413 | 0.958 | 2521.624 | 2386.032 | 0.950 | 268.181 | 265.546 | 0.961 | 2184.242 | 2100.892 |
| 8 | 0.994 | 237.704 | 234.662 | 0.992 | 1913.504 | 1758.546 | 0.963 | 270.812 | 267.833 | 0.970 | 1397.721 | 1291.280 |
| 9 | 0.991 | 202.134 | 199.387 | 0.949 | 1903.311 | 1874.913 | 0.988 | 229.305 | 226.573 | 0.857 | 1568.502 | 1549.905 |
| 10 | 0.985 | 155.490 | 152.093 | 0.981 | 749.083 | 735.585 | 0.982 | 168.100 | 165.024 | 0.906 | 659.730 | 649.202 |
| 11 | 0.981 | 111.594 | 107.077 | 0.994 | 210.975 | 195.289 | 0.964 | 124.389 | 120.403 | 0.964 | 252.223 | 235.561 |
| 12 | 0.973 | 79.961 | 76.502 | 0.997 | 109.961 | 94.042 | 0.962 | 81.035 | 77.834 | 0.994 | 117.441 | 101.937 |

Table S48 Test of the fit degree between the 30m resolution climate downscaling data and the monthly average temperature and monthly precipitation of 1 km.

| Month | cur1km&30m | | M26FH1km&30m | | M26LF1km&30m | | M45FH1km&30m | | M45LF1km&30m | | M85FH1km&30m | | M85LF1km&30m | |
| --- | --- | --- | --- | --- | --- | --- | --- | --- | --- | --- | --- | --- | --- | --- |
|  | Temper-ature | Precipit-ation | Temper-ature | Precipit-ation | Temper-ature | Precipit-ation | Temper-ature | Precipit-ation | Temper-ature | Precipit-ation | Temper-ature | Precipit-ation | Temper-ature | Precipit-ation |
| 1 | 0.997 | 0.999 | 0.924 | 0.995 | 0.945 | 0.988 | 0.941 | 0.996 | 0.944 | 0.990 | 0.948 | 0.993 | 0.944 | 0.993 |
| 2 | 0.997 | 0.999 | 0.925 | 0.989 | 0.929 | 0.992 | 0.922 | 0.996 | 0.923 | 0.994 | 0.925 | 0.995 | 0.921 | 0.993 |
| 3 | 0.997 | 0.999 | 0.923 | 0.994 | 0.929 | 0.980 | 0.914 | 0.995 | 0.914 | 0.998 | 0.911 | 0.988 | 0.922 | 0.993 |
| 4 | 0.997 | 0.999 | 0.954 | 0.914 | 0.956 | 0.973 | 0.955 | 0.970 | 0.949 | 0.970 | 0.954 | 0.959 | 0.947 | 0.956 |
| 5 | 0.998 | 0.999 | 0.970 | 0.974 | 0.971 | 0.973 | 0.971 | 0.959 | 0.970 | 0.991 | 0.968 | 0.975 | 0.967 | 0.968 |
| 6 | 0.998 | 0.998 | 0.989 | 0.978 | 0.989 | 0.984 | 0.986 | 0.907 | 0.988 | 0.993 | 0.988 | 0.966 | 0.989 | 0.977 |
| 7 | 0.998 | 0.998 | 0.990 | 0.911 | 0.991 | 0.910 | 0.990 | 0.906 | 0.989 | 0.972 | 0.990 | 0.918 | 0.992 | 0.940 |
| 8 | 0.998 | 0.998 | 0.992 | 0.977 | 0.992 | 0.981 | 0.991 | 0.992 | 0.991 | 0.992 | 0.991 | 0.986 | 0.990 | 0.934 |
| 9 | 0.998 | 0.998 | 0.983 | 0.944 | 0.984 | 0.927 | 0.985 | 0.904 | 0.983 | 0.951 | 0.985 | 0.908 | 0.985 | 0.813 |
| 10 | 0.998 | 0.999 | 0.978 | 0.950 | 0.976 | 0.888 | 0.975 | 0.882 | 0.975 | 0.974 | 0.975 | 0.965 | 0.977 | 0.867 |
| 11 | 0.998 | 0.999 | 0.962 | 0.959 | 0.972 | 0.973 | 0.969 | 0.957 | 0.970 | 0.996 | 0.969 | 0.988 | 0.967 | 0.948 |
| 12 | 0.998 | 0.999 | 0.959 | 0.978 | 0.949 | 0.995 | 0.962 | 0.997 | 0.959 | 0.998 | 0.952 | 0.996 | 0.962 | 0.995 |

Table S49 Total area (km^2^) of different land cover classes within the whole study area and mapped climate refugia across multi-scenarios in 2025.

| Class_Name | Whole Study Area | Refugia (1 km, No Dispersal) | Refugia (1 km, 10 m/yr Dispersal) | Refugia (30 m, No Dispersal) | Refugia (30 m, 10 m/yr Dispersal) |
| --- | --- | --- | --- | --- | --- |
| Cropland | 15256.39 | 563.42 | 286.88 | 232.53 | 118.53 |
| Forest | 77219.73 | 12243.06 | 6141.1 | 3767.71 | 2421.48 |
| Shrub | 1096.42 | 113.65 | 58.01 | 24.58 | 16.57 |
| Grassland | 32987.61 | 780.13 | 469.04 | 725 | 262.31 |
| Water | 729.54 | 3.63 | 2.28 | 12.51 | 5.3 |
| Snow/Ice | 678.82 | 15.97 | 9.06 | 8.77 | 4.35 |
| Barren | 1684.88 | 5.46 | 3.93 | 11.75 | 3.54 |
| Impervious | 937.66 | 2.57 | 0.78 | 6.02 | 2.27 |
| Wetland | 308.64 | 0 | 0 | 2.24 | 0 |

Table S50 Percentage composition (%) of different land cover classes within the whole study area and mapped climate refugia across multi-scenarios in 2025.

| Class_Name | Whole Study Area | Refugia (1 km, No Dispersal) | Refugia (1 km, 10 m/yr Dispersal) | Refugia (30 m, No Dispersal) | Refugia (30 m, 10 m/yr Dispersal) |
| --- | --- | --- | --- | --- | --- |
| Cropland | 11.66 | 4.1 | 4.12 | 4.85 | 4.18 |
| Forest | 58.99 | 89.18 | 88.09 | 78.64 | 85.43 |
| Shrub | 0.84 | 0.83 | 0.83 | 0.51 | 0.58 |
| Grassland | 25.2 | 5.68 | 6.73 | 15.13 | 9.25 |
| Water | 0.56 | 0.03 | 0.03 | 0.26 | 0.19 |
| Snow/Ice | 0.52 | 0.12 | 0.13 | 0.18 | 0.15 |
| Barren | 1.29 | 0.04 | 0.06 | 0.25 | 0.12 |
| Impervious | 0.72 | 0.02 | 0.01 | 0.13 | 0.08 |
| Wetland | 0.24 | 0 | 0 | 0.05 | 0 |

Table S51 Statistics of total and high-altitude (3000–4500 m) distribution characteristics for 20 bamboo species

under the SSP1-2.6 scenario based on the GISS-E2-1-G model

| Species | Data type | Baseline area | Baseline area (3000–4500 m) | Baseline proportion (3000–4500 m) (%) | G126_mid area | G126_mid area (3000–4500 m) | G126_mid proportion (3000–4500 m) (%) | G126_end area | G126_end area (3000–4500 m) | G126_end proportion (3000–4500 m) (%) |
| --- | --- | --- | --- | --- | --- | --- | --- | --- | --- | --- |
| *Chimonobambusa szechuanensis* | 1km | 8661 | 3 | 0.03% | 15218 | 10 | 0.07% | 14354 | 70 | 0.49% |
|  | 1kmdis10m |  |  |  | 3575 | 1 | 0.03% | 2772 | 1 | 0.04% |
|  | 30m | 8183.3202 | 2.0007 | 0.02% | 23052.7368 | 893.5731 | 3.88% | 23194.2654 | 1278.7236 | 5.51% |
|  | 30mdis10m |  |  |  | 10607.445 | 41.4459 | 0.39% | 13233.4434 | 53.5977 | 0.41% |
| *Yushania glauca* | 1km | 2609 | 258 | 9.89% | 22194 | 430 | 1.94% | 22507 | 600 | 2.67% |
|  | 1kmdis10m |  |  |  | 1879 | 102 | 5.43% | 1767 | 97 | 5.49% |
|  | 30m | 2681.4483 | 263.8773 | 9.84% | 2069.5977 | 677.8305 | 32.75% | 2058.768 | 533.4219 | 25.91% |
|  | 30mdis10m |  |  |  | 817.7292 | 88.3971 | 10.81% | 574.1757 | 90.3402 | 15.73% |
| *Phyllostachys bissetii* | 1km | 7103 | 0 | 0.00% | 3643 | 5 | 0.14% | 4366 | 15 | 0.34% |
|  | 1kmdis10m |  |  |  | 2592 | 0 | 0.00% | 2149 | 0 | 0.00% |
|  | 30m | 6406.9146 | 0.4185 | 0.01% | 10993.509 | 817.9866 | 7.44% | 13201.5474 | 626.7573 | 4.75% |
|  | 30mdis10m |  |  |  | 5260.2849 | 12.7998 | 0.24% | 7724.0421 | 24.7347 | 0.32% |
| *Yushania maculata* | 1km | 5778 | 1772 | 30.67% | 16031 | 1799 | 11.22% | 16762 | 1791 | 10.68% |
|  | 1kmdis10m |  |  |  | 4530 | 1355 | 29.91% | 3880 | 1122 | 28.92% |
|  | 30m | 5406.8625 | 1681.2603 | 31.09% | 26879.9283 | 2099.5965 | 7.81% | 18624.1788 | 1401.1812 | 7.52% |
|  | 30mdis10m |  |  |  | 10745.0667 | 1320.9741 | 12.29% | 8631.9207 | 974.5677 | 11.29% |
| *Fargesia scabrida* | 1km | 4876 | 173 | 3.55% | 1795 | 14 | 0.78% | 5084 | 17 | 0.33% |
|  | 1kmdis10m |  |  |  | 134 | 9 | 6.72% | 102 | 5 | 4.90% |
|  | 30m | 7459.812 | 793.4553 | 10.64% | 677.016 | 0.981 | 0.14% | 1383.0579 | 2.3679 | 0.17% |
|  | 30mdis10m |  |  |  | 573.6843 | 0.7065 | 0.12% | 723.3975 | 0.2097 | 0.03% |
| *Yushania dafengdingensis* | 1km | 5453 | 846 | 15.51% | 5956 | 1007 | 16.91% | 6580 | 938 | 14.26% |
|  | 1kmdis10m |  |  |  | 4013 | 705 | 17.57% | 3692 | 663 | 17.96% |
|  | 30m | 1624.689 | 277.8129 | 17.10% | 11584.0548 | 4187.1744 | 36.15% | 10137.0357 | 3870.7092 | 38.18% |
|  | 30mdis10m |  |  |  | 5340.3075 | 1609.5753 | 30.14% | 5663.4075 | 1977.2172 | 34.91% |
| *Yushania brevipaniculata* | 1km | 11495 | 635 | 5.52% | 1434 | 247 | 17.22% | 2064 | 145 | 7.03% |
|  | 1kmdis10m |  |  |  | 863 | 71 | 8.23% | 588 | 38 | 6.46% |
|  | 30m | 13616.19 | 768.9438 | 5.65% | 99.7398 | 49.1148 | 49.24% | 103.0761 | 44.3142 | 42.99% |
|  | 30mdis10m |  |  |  | 67.9383 | 29.097 | 42.83% | 38.7153 | 20.9646 | 54.15% |
| *Arundinaria spanostachya* | 1km | 2083 | 1393 | 66.87% | 3558 | 1707 | 47.98% | 1764 | 819 | 46.43% |
|  | 1kmdis10m |  |  |  | 1511 | 1004 | 66.45% | 852 | 506 | 59.39% |
|  | 30m | 1363.7655 | 1028.7909 | 75.44% | 2379.1617 | 698.5593 | 29.36% | 1611.6795 | 306.9117 | 19.04% |
|  | 30mdis10m |  |  |  | 238.3398 | 128.655 | 53.98% | 365.1174 | 184.9923 | 50.67% |
| *Fargesia ferax* | 1km | 9092 | 1128 | 12.41% | 1433 | 178 | 12.42% | 1841 | 215 | 11.68% |
|  | 1kmdis10m |  |  |  | 1415 | 170 | 12.01% | 1161 | 126 | 10.85% |
|  | 30m | 5022.6597 | 447.1425 | 8.90% | 28.1628 | 7.9344 | 28.17% | 28.4508 | 7.83 | 27.52% |
|  | 30mdis10m |  |  |  | 28.098 | 7.92 | 28.19% | 26.8569 | 7.7067 | 28.70% |
| *Fargesia robusta* | 1km | 6583 | 267 | 4.06% | 14522 | 1016 | 7.00% | 13587 | 675 | 4.97% |
|  | 1kmdis10m |  |  |  | 2125 | 80 | 3.76% | 1297 | 35 | 2.70% |
|  | 30m | 5650.8849 | 289.782 | 5.13% | 21058.5312 | 9955.7235 | 47.28% | 16776.5832 | 5612.0517 | 33.45% |
|  | 30mdis10m |  |  |  | 12117.843 | 4428.3609 | 36.54% | 12104.892 | 3724.1523 | 30.77% |
| *Fargesia nitida* | 1km | 17563 | 6627 | 37.73% | 21867 | 87 | 0.40% | 27493 | 84 | 0.31% |
|  | 1kmdis10m |  |  |  | 5104 | 79 | 1.55% | 5019 | 46 | 0.92% |
|  | 30m | 17192.2572 | 6639.8868 | 38.62% | 7865.6022 | 357.3693 | 4.54% | 10407.1608 | 225.2601 | 2.16% |
|  | 30mdis10m |  |  |  | 1023.9273 | 302.922 | 29.58% | 1483.2495 | 173.0574 | 11.67% |
| *Arundinaria faberi* | 1km | 18063 | 4875 | 26.99% | 5331 | 12 | 0.23% | 5894 | 20 | 0.34% |
|  | 1kmdis10m |  |  |  | 2567 | 12 | 0.47% | 1969 | 9 | 0.46% |
|  | 30m | 15221.7054 | 4219.3269 | 27.72% | 608.1822 | 273.4263 | 44.96% | 256.3317 | 58.0203 | 22.63% |
|  | 30mdis10m |  |  |  | 300.7341 | 220.0527 | 73.17% | 118.4589 | 47.4642 | 40.07% |
| *Yushania mabianensis* | 1km | 8325 | 915 | 10.99% | 5633 | 1490 | 26.45% | 6991 | 1890 | 27.03% |
|  | 1kmdis10m |  |  |  | 3189 | 648 | 20.32% | 2824 | 606 | 21.46% |
|  | 30m | 2765.8845 | 144.6372 | 5.23% | 2087.2926 | 42.0849 | 2.02% | 1969.9965 | 74.3526 | 3.77% |
|  | 30mdis10m |  |  |  | 1751.0085 | 32.0904 | 1.83% | 1424.0592 | 36.6678 | 2.57% |
| *Fargesia rufa* | 1km | 4129 | 51 | 1.24% | 363 | 25 | 6.89% | 1686 | 21 | 1.25% |
|  | 1kmdis10m |  |  |  | 64 | 9 | 14.06% | 21 | 4 | 19.05% |
|  | 30m | 5903.9262 | 324.5157 | 5.50% | 728.4699 | 171.0234 | 23.48% | 4234.2138 | 443.259 | 10.47% |
|  | 30mdis10m |  |  |  | 263.8206 | 101.9754 | 38.65% | 864.8199 | 151.5186 | 17.52% |
| *Chimonobambusa tumidissinoda* | 1km | 1975 | 37 | 1.87% | 1457 | 70 | 4.80% | 1886 | 173 | 9.17% |
|  | 1kmdis10m |  |  |  | 231 | 8 | 3.46% | 115 | 5 | 4.35% |
|  | 30m | 2016.4428 | 73.5921 | 3.65% | 3750.9696 | 1251.7344 | 33.37% | 6282.8964 | 2774.9097 | 44.17% |
|  | 30mdis10m |  |  |  | 836.9838 | 174.015 | 20.79% | 1341.0495 | 280.1727 | 20.89% |
| *Fargesia denudate* | 1km | 9463 | 2636 | 27.86% | 696 | 10 | 1.44% | 2736 | 47 | 1.72% |
|  | 1kmdis10m |  |  |  | 444 | 10 | 2.25% | 398 | 10 | 2.51% |
|  | 30m | 10640.826 | 3289.6809 | 30.92% | 3475.611 | 11.3283 | 0.33% | 6694.2 | 50.5953 | 0.76% |
|  | 30mdis10m |  |  |  | 2365.767 | 11.3184 | 0.48% | 3853.971 | 40.0806 | 1.04% |
| *Chimonobambusa opienensis* | 1km | 3087 | 10 | 0.32% | 25742 | 292 | 1.13% | 22162 | 330 | 1.49% |
|  | 1kmdis10m |  |  |  | 2945 | 9 | 0.31% | 2913 | 9 | 0.31% |
|  | 30m | 3567.3525 | 114.5889 | 3.21% | 53049.834 | 10417.9878 | 19.64% | 47893.0671 | 9287.5833 | 19.39% |
|  | 30mdis10m |  |  |  | 10292.1048 | 734.9499 | 7.14% | 13643.4312 | 1208.0889 | 8.85% |
| *Yushania lineolata* | 1km | 12052 | 2563 | 21.27% | 8294 | 492 | 5.93% | 7865 | 541 | 6.88% |
|  | 1kmdis10m |  |  |  | 3673 | 388 | 10.56% | 2821 | 167 | 5.92% |
|  | 30m | 12282.5601 | 2430.8352 | 19.79% | 2271.375 | 497.1753 | 21.89% | 984.6405 | 363.5136 | 36.92% |
|  | 30mdis10m |  |  |  | 2090.0394 | 482.2119 | 23.07% | 700.1298 | 253.1691 | 36.16% |
| *Fargesia obliqua* | 1km | 6045 | 1403 | 23.21% | 33 | 7 | 21.21% | 38 | 8 | 21.05% |
|  | 1kmdis10m |  |  |  | 33 | 7 | 21.21% | 32 | 6 | 18.75% |
|  | 30m | 5115.3462 | 1241.2341 | 24.26% | 50.0868 | 29.5389 | 58.98% | 333.1818 | 158.3361 | 47.52% |
|  | 30mdis10m |  |  |  | 31.8339 | 11.6604 | 36.63% | 222.5457 | 102.978 | 46.27% |
| *Yushania ailuropodina* | 1km | 6358 | 907 | 14.27% | 11687 | 1882 | 16.10% | 13317 | 2418 | 18.16% |
|  | 1kmdis10m |  |  |  | 5200 | 733 | 14.10% | 5094 | 704 | 13.82% |
|  | 30m | 3745.3851 | 766.557 | 20.47% | 29849.2497 | 5881.2966 | 19.70% | 25434.1116 | 5164.4133 | 20.31% |
|  | 30mdis10m |  |  |  | 10308.2679 | 3933.9234 | 38.16% | 10148.6358 | 3318.489 | 32.70% |

1. Data sources and periods: cur represents the actual distribution at the beginning of this century (baseline period, 2000s). gis2646 and gis2681 (or mri2646 and mri2681, depending on the specific table title) represent the projected results for the mid-21st century (2041–2060) and the end of the 21st century (2081–2100), respectively, under the corresponding Shared Socioeconomic Pathway (SSP) scenarios based on the GISS-E2-1-G model (or MRI-ESM2-0 model). The numbers in the column headers (e.g., 26, 45, 85) correspond to the SSP1-2.6, SSP2-4.5, and SSP5-8.5 scenarios, respectively.
2. Resolutions and Dispersal Limitations: 1km and 30m denote different spatial resolutions. Data rows labeled with dis10m represent the actual accessible or usable suitable habitats for each bamboo species under a strict dispersal capability limitation of 10 meters per year (10 m/yr).

Table S52 Statistics of total and high-altitude (3000–4500 m) distribution characteristics for 20 bamboo species under the SSP2-4.5 scenario based on the GISS-E2-1-G model

| Species | Data type | Baseline area | Baseline area (3000–4500 m) | Baseline proportion (3000–4500 m) (%) | G245_mid area | G245_mid area (3000–4500 m) | G245_mid proportion (3000–4500 m) (%) | G245_end area | G245_end area (3000–4500 m) | G245_end proportion (3000–4500 m) (%) |
| --- | --- | --- | --- | --- | --- | --- | --- | --- | --- | --- |
| *Chimonobambusa szechuanensis* | 1km | 8661 | 3 | 0.03% | 5218 | 7 | 0.13% | 4693 | 9 | 0.19% |
|  | 1kmdis10m |  |  |  | 3905 | 2 | 0.05% | 3218 | 2 | 0.06% |
|  | 30m | 8183.3202 | 2.0007 | 0.02% | 2419.9965 | 145.7244 | 6.02% | 3286.3446 | 870.9615 | 26.50% |
|  | 30mdis10m |  |  |  | 1897.1496 | 15.723 | 0.83% | 1599.6933 | 57.3165 | 3.58% |
| *Yushania glauca* | 1km | 2609 | 258 | 9.89% | 2027 | 590 | 29.11% | 1891 | 658 | 34.80% |
|  | 1kmdis10m |  |  |  | 1306 | 208 | 15.93% | 1148 | 191 | 16.64% |
|  | 30m | 2681.4483 | 263.8773 | 9.84% | 2402.6238 | 1576.7703 | 65.63% | 2590.2387 | 2080.4706 | 80.32% |
|  | 30mdis10m |  |  |  | 1042.4547 | 457.5546 | 43.89% | 609.2712 | 276.1668 | 45.33% |
| *Phyllostachys bissetii* | 1km | 7103 | 0 | 0.00% | 6007 | 8 | 0.13% | 5921 | 32 | 0.54% |
|  | 1kmdis10m |  |  |  | 3334 | 0 | 0.00% | 2753 | 0 | 0.00% |
|  | 30m | 6406.9146 | 0.4185 | 0.01% | 1830.2175 | 95.4225 | 5.21% | 2358.7074 | 293.9211 | 12.46% |
|  | 30mdis10m |  |  |  | 1011.4938 | 6.6096 | 0.65% | 1047.6099 | 19.4616 | 1.86% |
| *Yushania maculata* | 1km | 5778 | 1772 | 30.67% | 5420 | 3076 | 56.75% | 5726 | 3479 | 60.76% |
|  | 1kmdis10m |  |  |  | 3379 | 1573 | 46.55% | 2965 | 1497 | 50.49% |
|  | 30m | 5406.8625 | 1681.2603 | 31.09% | 2194.4817 | 1965.2634 | 89.55% | 2546.7741 | 2057.7231 | 80.80% |
|  | 30mdis10m |  |  |  | 1583.9334 | 1418.1363 | 89.53% | 1690.9902 | 1380.4452 | 81.64% |
| *Fargesia scabrida* | 1km | 4876 | 173 | 3.55% | 3041 | 436 | 14.34% | 3180 | 642 | 20.19% |
|  | 1kmdis10m |  |  |  | 1744 | 64 | 3.67% | 1416 | 61 | 4.31% |
|  | 30m | 7459.812 | 793.4553 | 10.64% | 3398.2623 | 2568.2067 | 75.57% | 1797.0264 | 1343.5839 | 74.77% |
|  | 30mdis10m |  |  |  | 1054.0449 | 445.2021 | 42.24% | 1164.7593 | 779.76 | 66.95% |
| *Yushania dafengdingensis* | 1km | 5453 | 846 | 15.51% | 5128 | 931 | 18.16% | 6456 | 1107 | 17.15% |
|  | 1kmdis10m |  |  |  | 4480 | 727 | 16.23% | 4387 | 721 | 16.43% |
|  | 30m | 1624.689 | 277.8129 | 17.10% | 5109.9696 | 2397.9834 | 46.93% | 9462.2211 | 4049.0811 | 42.79% |
|  | 30mdis10m |  |  |  | 3168.9567 | 999.684 | 31.55% | 5284.4076 | 1834.236 | 34.71% |
| *Yushania brevipaniculata* | 1km | 11495 | 635 | 5.52% | 13811 | 2960 | 21.43% | 12999 | 3370 | 25.93% |
|  | 1kmdis10m |  |  |  | 8276 | 515 | 6.22% | 7200 | 509 | 7.07% |
|  | 30m | 13616.19 | 768.9438 | 5.65% | 16179.714 | 5414.8086 | 33.47% | 16032.8592 | 7960.5225 | 49.65% |
|  | 30mdis10m |  |  |  | 11973.123 | 2503.3752 | 20.91% | 10874.3472 | 3756.1599 | 34.54% |
| *Arundinaria spanostachya* | 1km | 2083 | 1393 | 66.87% | 1007 | 848 | 84.21% | 735 | 637 | 86.67% |
|  | 1kmdis10m |  |  |  | 641 | 524 | 81.75% | 364 | 304 | 83.52% |
|  | 30m | 1363.7655 | 1028.7909 | 75.44% | 106.4916 | 86.202 | 80.95% | 42.39 | 33.7203 | 79.55% |
|  | 30mdis10m |  |  |  | 66.8205 | 60.1884 | 90.07% | 20.4525 | 18.1008 | 88.50% |
| *Fargesia ferax* | 1km | 9092 | 1128 | 12.41% | 9195 | 2038 | 22.16% | 8631 | 1894 | 21.94% |
|  | 1kmdis10m |  |  |  | 7114 | 1012 | 14.23% | 6544 | 926 | 14.15% |
|  | 30m | 5022.6597 | 447.1425 | 8.90% | 1917.5319 | 738.6309 | 38.52% | 1728.8586 | 472.4775 | 27.33% |
|  | 30mdis10m |  |  |  | 1621.3014 | 550.4868 | 33.95% | 1480.3731 | 391.3227 | 26.43% |
| *Fargesia robusta* | 1km | 6583 | 267 | 4.06% | 9414 | 1919 | 20.38% | 10457 | 2613 | 24.99% |
|  | 1kmdis10m |  |  |  | 4647 | 178 | 3.83% | 4430 | 175 | 3.95% |
|  | 30m | 5650.8849 | 289.782 | 5.13% | 21901.1589 | 10604.5695 | 48.42% | 27604.8693 | 16016.274 | 58.02% |
|  | 30mdis10m |  |  |  | 14434.2504 | 4483.0296 | 31.06% | 17505.2718 | 6971.4837 | 39.83% |
| *Fargesia nitida* | 1km | 17563 | 6627 | 37.73% | 22417 | 14964 | 66.75% | 23949 | 16979 | 70.90% |
|  | 1kmdis10m |  |  |  | 13340 | 6372 | 47.77% | 12649 | 6318 | 49.95% |
|  | 30m | 17192.2572 | 6639.8868 | 38.62% | 25057.773 | 21172.3002 | 84.49% | 28359.9288 | 23836.5324 | 84.05% |
|  | 30mdis10m |  |  |  | 16062.8103 | 12799.4571 | 79.68% | 18291.6369 | 15353.5383 | 83.94% |
| *Arundinaria faberi* | 1km | 18063 | 4875 | 26.99% | 14274 | 7279 | 50.99% | 13219 | 7370 | 55.75% |
|  | 1kmdis10m |  |  |  | 10119 | 3920 | 38.74% | 8597 | 3592 | 41.78% |
|  | 30m | 15221.7054 | 4219.3269 | 27.72% | 15977.9781 | 9726.4287 | 60.87% | 17668.6731 | 12462.6411 | 70.54% |
|  | 30mdis10m |  |  |  | 12986.3007 | 7005.6783 | 53.95% | 13997.2734 | 9101.3274 | 65.02% |
| *Yushania mabianensis* | 1km | 8325 | 915 | 10.99% | 9051 | 1815 | 20.05% | 10034 | 2121 | 21.14% |
|  | 1kmdis10m |  |  |  | 5953 | 827 | 13.89% | 5791 | 820 | 14.16% |
|  | 30m | 2765.8845 | 144.6372 | 5.23% | 405.0045 | 118.0575 | 29.15% | 289.629 | 81.3852 | 28.10% |
|  | 30mdis10m |  |  |  | 395.397 | 110.5407 | 27.96% | 273.4101 | 72.6984 | 26.59% |
| *Fargesia rufa* | 1km | 4129 | 51 | 1.24% | 3081 | 104 | 3.38% | 3368 | 173 | 5.14% |
|  | 1kmdis10m |  |  |  | 1760 | 15 | 0.85% | 1532 | 13 | 0.85% |
|  | 30m | 5903.9262 | 324.5157 | 5.50% | 4393.0422 | 3086.406 | 70.26% | 6137.478 | 4757.004 | 77.51% |
|  | 30mdis10m |  |  |  | 1979.8992 | 1226.5443 | 61.95% | 2702.3274 | 2074.5864 | 76.77% |
| *Chimonobambusa tumidissinoda* | 1km | 1975 | 37 | 1.87% | 1041 | 71 | 6.82% | 640 | 76 | 11.88% |
|  | 1kmdis10m |  |  |  | 736 | 5 | 0.68% | 396 | 5 | 1.26% |
|  | 30m | 2016.4428 | 73.5921 | 3.65% | 497.2887 | 325.1043 | 65.38% | 387.4212 | 295.9992 | 76.40% |
|  | 30mdis10m |  |  |  | 116.5869 | 34.1181 | 29.26% | 75.8367 | 31.0581 | 40.95% |
| *Fargesia denudate* | 1km | 9463 | 2636 | 27.86% | 6795 | 2442 | 35.94% | 7011 | 2381 | 33.96% |
|  | 1kmdis10m |  |  |  | 5957 | 1775 | 29.80% | 5624 | 1609 | 28.61% |
|  | 30m | 10640.826 | 3289.6809 | 30.92% | 1669.5018 | 861.2577 | 51.59% | 1393.1181 | 735.5799 | 52.80% |
|  | 30mdis10m |  |  |  | 1583.8488 | 776.484 | 49.03% | 1252.4283 | 610.6806 | 48.76% |
| *Chimonobambusa opienensis* | 1km | 3087 | 10 | 0.32% | 1899 | 144 | 7.58% | 1842 | 219 | 11.89% |
|  | 1kmdis10m |  |  |  | 1245 | 9 | 0.72% | 944 | 7 | 0.74% |
|  | 30m | 3567.3525 | 114.5889 | 3.21% | 5983.7157 | 2002.7511 | 33.47% | 9045.0594 | 5027.9373 | 55.59% |
|  | 30mdis10m |  |  |  | 1101.4893 | 265.8267 | 24.13% | 1612.0107 | 404.4771 | 25.09% |
| *Yushania lineolata* | 1km | 12052 | 2563 | 21.27% | 9677 | 3498 | 36.15% | 9477 | 3582 | 37.80% |
|  | 1kmdis10m |  |  |  | 6912 | 2020 | 29.22% | 5883 | 1841 | 31.29% |
|  | 30m | 12282.5601 | 2430.8352 | 19.79% | 6147.4023 | 1906.7229 | 31.02% | 8415.6084 | 2335.3128 | 27.75% |
|  | 30mdis10m |  |  |  | 5720.6817 | 1602.9846 | 28.02% | 7922.0277 | 2081.0916 | 26.27% |
| *Fargesia obliqua* | 1km | 6045 | 1403 | 23.21% | 5594 | 2033 | 36.34% | 4742 | 1801 | 37.98% |
|  | 1kmdis10m |  |  |  | 3599 | 967 | 26.87% | 3046 | 806 | 26.46% |
|  | 30m | 5115.3462 | 1241.2341 | 24.26% | 13196.2005 | 10220.6943 | 77.45% | 11039.8608 | 8860.3875 | 80.26% |
|  | 30mdis10m |  |  |  | 7263.9405 | 4486.5477 | 61.76% | 6273.6597 | 4131.0441 | 65.85% |
| *Yushania ailuropodina* | 1km | 6358 | 907 | 14.27% | 3876 | 1171 | 30.21% | 3022 | 1066 | 35.27% |
|  | 1kmdis10m |  |  |  | 3056 | 726 | 23.76% | 2092 | 608 | 29.06% |
|  | 30m | 3745.3851 | 766.557 | 20.47% | 3653.2746 | 3227.5998 | 88.35% | 4897.2258 | 4059.4248 | 82.89% |
|  | 30mdis10m |  |  |  | 2831.2218 | 2509.9848 | 88.65% | 3396.2949 | 2935.2582 | 86.43% |

Note: The definitions of abbreviations and indicators in this table are identical to those in Table S51.

Table S53 Statistics of total and high-altitude (3000–4500 m) distribution characteristics for 20 bamboo species under the SSP5-8.5 scenario based on the GISS-E2-1-G model

| Species | Data type | Baseline area | Baseline area (3000–4500 m) | Baseline proportion (3000–4500 m) (%) | G585_mid area | G585_mid area (3000–4500 m) | G585_mid proportion (3000–4500 m) (%) | G585_end area | G585_end area (3000–4500 m) | G585_end proportion (3000–4500 m) (%) |
| --- | --- | --- | --- | --- | --- | --- | --- | --- | --- | --- |
| *Chimonobambusa szechuanensis* | 1km | 8661 | 3 | 0.03% | 5106  3582 | 23 | 0.45% | 3624 | 325 | 8.97% |
|  | 1kmdis10m |  |  |  |  | 3 | 0.08% | 1404 | 3 | 0.21% |
|  | 30m | 8183.3202 | 2.0007 | 0.02% | 3871.6911 | 731.475 | 18.89% | 9423.6588 | 4303.9575 | 45.67% |
|  | 30mdis10m |  |  |  | 2119.9581 | 42.1866 | 1.99% | 1969.7661 | 178.3854 | 9.06% |
| *Yushania glauca* | 1km | 2609 | 258 | 9.89% | 2184 | 805 | 36.86% | 3335 | 2407 | 72.17% |
|  | 1kmdis10m |  |  |  | 1211 | 191 | 15.77% | 432 | 96 | 22.22% |
|  | 30m | 2681.4483 | 263.8773 | 9.84% | 3061.5579 | 2646.1854 | 86.43% | 2856.2202 | 2470.1877 | 86.48% |
|  | 30mdis10m |  |  |  | 447.7437 | 198.8712 | 44.42% | 30.267 | 4.8204 | 15.93% |
| *Phyllostachys bissetii* | 1km | 7103 | 0 | 0.00% | 5863 | 21 | 0.36% | 2734 | 113 | 4.13% |
|  | 1kmdis10m |  |  |  | 2616 | 0 | 0.00% | 534 | 0 | 0.00% |
|  | 30m | 6406.9146 | 0.4185 | 0.01% | 3174.4377 | 499.5135 | 15.74% | 2636.4555 | 2202.8202 | 83.55% |
|  | 30mdis10m |  |  |  | 1035.3699 | 9.4392 | 0.91% | 119.6739 | 35.1135 | 29.34% |
| *Yushania maculata* | 1km | 5778 | 1772 | 30.67% | 6387 | 3638 | 56.96% | 8550 | 7069 | 82.68% |
|  | 1kmdis10m |  |  |  | 3614 | 1633 | 45.19% | 1724 | 1203 | 69.78% |
|  | 30m | 5406.8625 | 1681.2603 | 31.09% | 4575.5118 | 3885.7122 | 84.92% | 1513.1736 | 335.2545 | 22.16% |
|  | 30mdis10m |  |  |  | 2669.2425 | 2279.5038 | 85.40% | 720.0702 | 141.0219 | 19.58% |
| *Fargesia scabrida* | 1km | 4876 | 173 | 3.55% | 2598 | 483 | 18.59% | 1463 | 1055 | 72.11% |
|  | 1kmdis10m |  |  |  | 1322 | 70 | 5.30% | 157 | 50 | 31.85% |
|  | 30m | 7459.812 | 793.4553 | 10.64% | 424.7145 | 92.6604 | 21.82% | 65.3832 | 50.0166 | 76.50% |
|  | 30mdis10m |  |  |  | 342.2313 | 62.3961 | 18.23% | 14.8437 | 4.4829 | 30.20% |
| *Yushania dafengdingensis* | 1km | 5453 | 846 | 15.51% | 5241 | 1077 | 20.55% | 10535 | 2019 | 19.16% |
|  | 1kmdis10m |  |  |  | 4101 | 720 | 17.56% | 4080 | 720 | 17.65% |
|  | 30m | 1624.689 | 277.8129 | 17.10% | 5727.8763 | 3515.1741 | 61.37% | 21790.6821 | 6005.0871 | 27.56% |
|  | 30mdis10m |  |  |  | 2905.3863 | 1315.7874 | 45.29% | 6498.6129 | 2011.3992 | 30.95% |
| *Yushania brevipaniculata* | 1km | 11495 | 635 | 5.52% | 12920 | 3270 | 25.31% | 14251 | 5514 | 38.69% |
|  | 1kmdis10m |  |  |  | 6644 | 532 | 8.01% | 4383 | 473 | 10.79% |
|  | 30m | 13616.19 | 768.9438 | 5.65% | 15823.719 | 6767.1675 | 42.77% | 24368.8707 | 12424.041 | 50.98% |
|  | 30mdis10m |  |  |  | 10192.23 | 2699.9379 | 26.49% | 8075.5272 | 3018.2121 | 37.37% |
| *Arundinaria spanostachya* | 1km | 2083 | 1393 | 66.87% | 1417 | 1163 | 82.07% | 2141 | 1472 | 68.75% |
|  | 1kmdis10m |  |  |  | 702 | 565 | 80.48% | 311 | 214 | 68.81% |
|  | 30m | 1363.7655 | 1028.7909 | 75.44% | 258.804 | 237.0357 | 91.59% | 10.1988 | 0.2241 | 2.20% |
|  | 30mdis10m |  |  |  | 16.5852 | 15.2343 | 91.85% | 0.0846 | 0 | 0.00% |
| *Fargesia ferax* | 1km | 9092 | 1128 | 12.41% | 6875 | 1260 | 18.33% | 7502 | 1957 | 26.09% |
|  | 1kmdis10m |  |  |  | 5200 | 647 | 12.44% | 4646 | 590 | 12.70% |
|  | 30m | 5022.6597 | 447.1425 | 8.90% | 1011.1095 | 290.0763 | 28.69% | 2107.4373 | 187.1145 | 8.88% |
|  | 30mdis10m |  |  |  | 842.1327 | 198.2385 | 23.54% | 946.9953 | 100.0854 | 10.57% |
| *Fargesia robusta* | 1km | 6583 | 267 | 4.06% | 9973 | 2403 | 24.10% | 15966 | 7322 | 45.86% |
|  | 1kmdis10m |  |  |  | 4554 | 219 | 4.81% | 4048 | 219 | 5.41% |
|  | 30m | 5650.8849 | 289.782 | 5.13% | 25109.253 | 14096.4327 | 56.14% | 41595.5358 | 23736.4011 | 57.06% |
|  | 30mdis10m |  |  |  | 14407.6131 | 5089.5621 | 35.33% | 18328.6179 | 6919.2171 | 37.75% |
| *Fargesia nitida* | 1km | 17563 | 6627 | 37.73% | 23476 | 16202 | 69.02% | 24758 | 20929 | 84.53% |
|  | 1kmdis10m |  |  |  | 13364 | 6449 | 48.26% | 8931 | 5860 | 65.61% |
|  | 30m | 17192.2572 | 6639.8868 | 38.62% | 24671.8278 | 20653.4565 | 83.71% | 12367.206 | 9800.3754 | 79.24% |
|  | 30mdis10m |  |  |  | 15444.567 | 12369.9789 | 80.09% | 8021.4048 | 6780.2391 | 84.53% |
| *Arundinaria faberi* | 1km | 18063 | 4875 | 26.99% | 14276 | 7758 | 54.34% | 15190 | 11455 | 75.41% |
|  | 1kmdis10m |  |  |  | 8381 | 3312 | 39.52% | 4270 | 2335 | 54.68% |
|  | 30m | 15221.7054 | 4219.3269 | 27.72% | 14995.3914 | 9667.6695 | 64.47% | 17472.3894 | 11823.9372 | 67.67% |
|  | 30mdis10m |  |  |  | 11070.8442 | 6180.1227 | 55.82% | 8735.3793 | 5303.3949 | 60.71% |
| *Yushania mabianensis* | 1km | 8325 | 915 | 10.99% | 9904 | 2185 | 22.06% | 14771 | 6131 | 41.51% |
|  | 1kmdis10m |  |  |  | 5695 | 820 | 14.40% | 5325 | 818 | 15.36% |
|  | 30m | 2765.8845 | 144.6372 | 5.23% | 69.5295 | 19.0494 | 27.40% | 8.7246 | 2.4363 | 27.92% |
|  | 30mdis10m |  |  |  | 63.5445 | 14.4927 | 22.81% | 7.5807 | 2.2779 | 30.05% |
| *Fargesia rufa* | 1km | 4129 | 51 | 1.24% | 1959 | 75 | 3.83% | 1001 | 294 | 29.37% |
|  | 1kmdis10m |  |  |  | 1084 | 13 | 1.20% | 119 | 6 | 5.04% |
|  | 30m | 5903.9262 | 324.5157 | 5.50% | 2124.3195 | 1521.6237 | 71.63% | 489.9681 | 487.6434 | 99.53% |
|  | 30mdis10m |  |  |  | 1076.9319 | 801.891 | 74.46% | 15.6609 | 15.6222 | 99.75% |
| *Chimonobambusa tumidissinoda* | 1km | 1975 | 37 | 1.87% | 665 | 90 | 13.53% | 46 | 7 | 15.22% |
|  | 1kmdis10m |  |  |  | 395 | 10 | 2.53% | 18 | 0 | 0.00% |
|  | 30m | 2016.4428 | 73.5921 | 3.65% | 206.739 | 122.4666 | 59.24% | 12.3471 | 6.3909 | 51.76% |
|  | 30mdis10m |  |  |  | 62.8758 | 38.3301 | 60.96% | 1.6002 | 1.3671 | 85.43% |
| *Fargesia denudate* | 1km | 9463 | 2636 | 27.86% | 4203 | 1406 | 33.45% | 1275 | 336 | 26.35% |
|  | 1kmdis10m |  |  |  | 3936 | 1177 | 29.90% | 1068 | 220 | 20.60% |
|  | 30m | 10640.826 | 3289.6809 | 30.92% | 389.3697 | 179.829 | 46.18% | 66.0285 | 15.3963 | 23.32% |
|  | 30mdis10m |  |  |  | 374.7852 | 165.3597 | 44.12% | 61.2585 | 11.1924 | 18.27% |
| *Chimonobambusa opienensis* | 1km | 3087 | 10 | 0.32% | 2662 | 247 | 9.28% | 784 | 140 | 17.86% |
|  | 1kmdis10m |  |  |  | 1229 | 6 | 0.49% | 95 | 3 | 3.16% |
|  | 30m | 3567.3525 | 114.5889 | 3.21% | 12003.7284 | 4797.2592 | 39.96% | 5878.017 | 882.5067 | 15.01% |
|  | 30mdis10m |  |  |  | 1603.0512 | 380.241 | 23.72% | 891.1008 | 245.9583 | 27.60% |
| *Yushania lineolata* | 1km | 12052 | 2563 | 21.27% | 9183 | 3279 | 35.71% | 7331 | 4161 | 56.76% |
|  | 1kmdis10m |  |  |  | 6071 | 1723 | 28.38% | 2968 | 947 | 31.91% |
|  | 30m | 12282.5601 | 2430.8352 | 19.79% | 5172.5304 | 1352.3463 | 26.14% | 5872.5333 | 1009.9206 | 17.20% |
|  | 30mdis10m |  |  |  | 4631.9229 | 1065.9096 | 23.01% | 4351.8978 | 812.385 | 18.67% |
| *Fargesia obliqua* | 1km | 6045 | 1403 | 23.21% | 3462 | 1284 | 37.09% | 3754 | 1594 | 42.46% |
|  | 1kmdis10m |  |  |  | 1936 | 571 | 29.49% | 1145 | 310 | 27.07% |
|  | 30m | 5115.3462 | 1241.2341 | 24.26% | 6766.3872 | 6062.7537 | 89.60% | 189.5166 | 127.0404 | 67.03% |
|  | 30mdis10m |  |  |  | 2460.4992 | 1834.6338 | 74.56% | 94.1679 | 42.9219 | 45.58% |
| *Yushania ailuropodina* | 1km | 6358 | 907 | 14.27% | 3744 | 1479 | 39.50% | 1828 | 1137 | 62.20% |
|  | 1kmdis10m |  |  |  | 1934 | 553 | 28.59% | 555 | 257 | 46.31% |
|  | 30m | 3745.3851 | 766.557 | 20.47% | 5041.2222 | 4509.9972 | 89.46% | 851.3604 | 561.8196 | 65.99% |
|  | 30mdis10m |  |  |  | 3689.1207 | 3282.1974 | 88.97% | 450.918 | 361.4364 | 80.16% |

Note: The definitions of abbreviations and indicators in this table are identical to those in Table S51.

Table S54 Statistics of total and high-altitude (3000–4500 m) distribution characteristics for 20 bamboo species under the SSP1-2.6 scenario based on the MRI-ESM2-0 model

| Species | Data type | Baseline area | Baseline area (3000–4500 m) | Baseline proportion (3000–4500 m) (%) | M126_mid area | M126_mid area (3000–4500 m) | M126_mid proportion (3000–4500 m) (%) | M126_end area | M126_end area (3000–4500 m) | M126_end proportion (3000–4500 m) (%) |
| --- | --- | --- | --- | --- | --- | --- | --- | --- | --- | --- |
| *Chimonobambusa szechuanensis* | 1km | 8661 | 3 | 0.03% | 13466 | 2 | 0.01% | 12758 | 3 | 0.02% |
|  | 1kmdis10m |  |  |  | 4219 | 1 | 0.02% | 3412 | 0 | 0.00% |
|  | 30m | 8183.3202 | 2.0007 | 0.02% | 14316.6663 | 870.3531 | 6.08% | 15389.7372 | 951.1047 | 6.18% |
|  | 30mdis10m |  |  |  | 6034.9005 | 21.6234 | 0.36% | 7033.2867 | 39.6351 | 0.56% |
| *Yushania glauca* | 1km | 2609 | 258 | 9.89% | 16970 | 440 | 2.59% | 19645 | 613 | 3.12% |
|  | 1kmdis10m |  |  |  | 1918 | 155 | 8.08% | 1725 | 137 | 7.94% |
|  | 30m | 2681.4483 | 263.8773 | 9.84% | 2906.1585 | 637.6752 | 21.94% | 2466.4338 | 636.1614 | 25.79% |
|  | 30mdis10m |  |  |  | 990.3969 | 217.854 | 22.00% | 821.781 | 186.6933 | 22.72% |
| *Phyllostachys bissetii* | 1km | 7103 | 0 | 0.00% | 5398 | 6 | 0.11% | 5102 | 0 | 0.00% |
|  | 1kmdis10m |  |  |  | 2765 | 0 | 0.00% | 2040 | 0 | 0.00% |
|  | 30m | 6406.9146 | 0.4185 | 0.01% | 12719.2977 | 160.173 | 1.26% | 14169.1491 | 684.1395 | 4.83% |
|  | 30mdis10m |  |  |  | 5581.5867 | 5.4279 | 0.10% | 6177.2895 | 11.502 | 0.19% |
| *Yushania maculata* | 1km | 5778 | 1772 | 30.67% | 14454 | 1607 | 11.12% | 14638 | 1469 | 10.04% |
|  | 1kmdis10m |  |  |  | 3992 | 1150 | 28.81% | 2712 | 595 | 21.94% |
|  | 30m | 5406.8625 | 1681.2603 | 31.09% | 10156.8042 | 802.0539 | 7.90% | 8931.8412 | 369.5067 | 4.14% |
|  | 30mdis10m |  |  |  | 2576.7396 | 308.916 | 11.99% | 2825.3547 | 192.2859 | 6.81% |
| *Fargesia scabrida* | 1km | 4876 | 173 | 3.55% | 13394 | 286 | 2.14% | 12826 | 246 | 1.92% |
|  | 1kmdis10m |  |  |  | 674 | 25 | 3.71% | 460 | 22 | 4.78% |
|  | 30m | 7459.812 | 793.4553 | 10.64% | 2982.6585 | 119.295 | 4.00% | 3329.2485 | 523.908 | 15.74% |
|  | 30mdis10m |  |  |  | 1075.2921 | 22.8474 | 2.12% | 1027.6308 | 3.3894 | 0.33% |
| *Yushania dafengdingensis* | 1km | 5453 | 846 | 15.51% | 6933 | 575 | 8.29% | 9853 | 879 | 8.92% |
|  | 1kmdis10m |  |  |  | 4125 | 522 | 12.65% | 4111 | 516 | 12.55% |
|  | 30m | 1624.689 | 277.8129 | 17.10% | 6230.5137 | 2048.9535 | 32.89% | 11094.5601 | 3718.2501 | 33.51% |
|  | 30mdis10m |  |  |  | 3362.3829 | 1137.5766 | 33.83% | 5359.6053 | 1752.4098 | 32.70% |
| *Yushania brevipaniculata* | 1km | 11495 | 635 | 5.52% | 3353 | 53 | 1.58% | 2412 | 62 | 2.57% |
|  | 1kmdis10m |  |  |  | 1186 | 21 | 1.77% | 539 | 12 | 2.23% |
|  | 30m | 13616.19 | 768.9438 | 5.65% | 258.2694 | 94.5594 | 36.61% | 251.2485 | 100.4319 | 39.97% |
|  | 30mdis10m |  |  |  | 45.8874 | 5.1876 | 11.31% | 27.1377 | 2.8368 | 10.45% |
| *Arundinaria spanostachya* | 1km | 2083 | 1393 | 66.87% | 2915 | 1485 | 50.94% | 1170 | 595 | 50.85% |
|  | 1kmdis10m |  |  |  | 1121 | 772 | 68.87% | 439 | 302 | 68.79% |
|  | 30m | 1363.7655 | 1028.7909 | 75.44% | 764.2719 | 367.1613 | 48.04% | 697.0446 | 250.7769 | 35.98% |
|  | 30mdis10m |  |  |  | 317.7981 | 195.0345 | 61.37% | 322.8525 | 200.7522 | 62.18% |
| *Fargesia ferax* | 1km | 9092 | 1128 | 12.41% | 1880 | 272 | 14.47% | 1484 | 353 | 23.79% |
|  | 1kmdis10m |  |  |  | 1654 | 268 | 16.20% | 854 | 163 | 19.09% |
|  | 30m | 5022.6597 | 447.1425 | 8.90% | 48.7971 | 27.4914 | 56.34% | 26.8794 | 7.7508 | 28.84% |
|  | 30mdis10m |  |  |  | 29.2329 | 8.8182 | 30.17% | 26.7462 | 7.7391 | 28.94% |
| *Fargesia robusta* | 1km | 6583 | 267 | 4.06% | 15864 | 120 | 0.76% | 16104 | 87 | 0.54% |
|  | 1kmdis10m |  |  |  | 1351 | 8 | 0.59% | 910 | 1 | 0.11% |
|  | 30m | 5650.8849 | 289.782 | 5.13% | 14163.4134 | 339.4485 | 2.40% | 15878.3967 | 1401.2253 | 8.82% |
|  | 30mdis10m |  |  |  | 7239.8754 | 271.5156 | 3.75% | 7772.3352 | 532.2987 | 6.85% |
| *Fargesia nitida* | 1km | 17563 | 6627 | 37.73% | 33507 | 79 | 0.24% | 32019 | 43 | 0.13% |
|  | 1kmdis10m |  |  |  | 5318 | 66 | 1.24% | 4522 | 13 | 0.29% |
|  | 30m | 17192.2572 | 6639.8868 | 38.62% | 15476.3109 | 139.0086 | 0.90% | 11931.8175 | 172.5561 | 1.45% |
|  | 30mdis10m |  |  |  | 2075.2884 | 114.0894 | 5.50% | 1620.9729 | 90.1134 | 5.56% |
| *Arundinaria faberi* | 1km | 18063 | 4875 | 26.99% | 8798 | 9 | 0.10% | 7256 | 3 | 0.04% |
|  | 1kmdis10m |  |  |  | 3265 | 8 | 0.25% | 1843 | 1 | 0.05% |
|  | 30m | 15221.7054 | 4219.3269 | 27.72% | 2446.9884 | 69.7527 | 2.85% | 780.7725 | 54.7776 | 7.02% |
|  | 30mdis10m |  |  |  | 259.137 | 61.6392 | 23.79% | 151.5645 | 45.729 | 30.17% |
| *Yushania mabianensis* | 1km | 8325 | 915 | 10.99% | 5195 | 984 | 18.94% | 6015 | 1327 | 22.06% |
|  | 1kmdis10m |  |  |  | 2763 | 501 | 18.13% | 2508 | 492 | 19.62% |
|  | 30m | 2765.8845 | 144.6372 | 5.23% | 3629.8233 | 152.5365 | 4.20% | 2620.1232 | 106.3494 | 4.06% |
|  | 30mdis10m |  |  |  | 2340.7056 | 97.0371 | 4.15% | 1868.1858 | 63.1449 | 3.38% |
| *Fargesia rufa* | 1km | 4129 | 51 | 1.24% | 4391 | 318 | 7.24% | 5378 | 767 | 14.26% |
|  | 1kmdis10m |  |  |  | 1130 | 30 | 2.65% | 961 | 30 | 3.12% |
|  | 30m | 5903.9262 | 324.5157 | 5.50% | 6742.7271 | 3116.2689 | 46.22% | 9827.0865 | 4870.8639 | 49.57% |
|  | 30mdis10m |  |  |  | 1498.7412 | 314.3961 | 20.98% | 2224.4616 | 807.0975 | 36.28% |
| *Chimonobambusa tumidissinoda* | 1km | 1975 | 37 | 1.87% | 2981 | 27 | 0.91% | 2872 | 47 | 1.64% |
|  | 1kmdis10m |  |  |  | 465 | 7 | 1.51% | 239 | 5 | 2.09% |
|  | 30m | 2016.4428 | 73.5921 | 3.65% | 3579.5412 | 392.7294 | 10.97% | 4539.1446 | 222.7725 | 4.91% |
|  | 30mdis10m |  |  |  | 977.7114 | 55.2168 | 5.65% | 1193.6817 | 13.2264 | 1.11% |
| *Fargesia denudate* | 1km | 9463 | 2636 | 27.86% | 16331 | 986 | 6.04% | 20491 | 2322 | 11.33% |
|  | 1kmdis10m |  |  |  | 5358 | 717 | 13.38% | 5075 | 648 | 12.77% |
|  | 30m | 10640.826 | 3289.6809 | 30.92% | 10680.2244 | 761.6133 | 7.13% | 10709.8254 | 957.2175 | 8.94% |
|  | 30mdis10m |  |  |  | 5369.2542 | 583.6932 | 10.87% | 6428.0133 | 657.36 | 10.23% |
| *Chimonobambusa opienensis* | 1km | 3087 | 10 | 0.32% | 10262 | 203 | 1.98% | 12035 | 272 | 2.26% |
|  | 1kmdis10m |  |  |  | 2407 | 8 | 0.33% | 1876 | 5 | 0.27% |
|  | 30m | 3567.3525 | 114.5889 | 3.21% | 22328.5932 | 1718.8308 | 7.70% | 20090.4678 | 2795.1345 | 13.91% |
|  | 30mdis10m |  |  |  | 3428.7372 | 332.1792 | 9.69% | 3900.0051 | 399.3651 | 10.24% |
| *Yushania lineolata* | 1km | 12052 | 2563 | 21.27% | 5642 | 286 | 5.07% | 5280 | 476 | 9.02% |
|  | 1kmdis10m |  |  |  | 2693 | 207 | 7.69% | 1953 | 93 | 4.76% |
|  | 30m | 12282.5601 | 2430.8352 | 19.79% | 992.1141 | 199.1907 | 20.08% | 732.8475 | 186.9156 | 25.51% |
|  | 30mdis10m |  |  |  | 919.0269 | 146.4867 | 15.94% | 621.6804 | 158.5224 | 25.50% |
| *Fargesia obliqua* | 1km | 6045 | 1403 | 23.21% | 110 | 26 | 23.64% | 1828 | 493 | 26.97% |
|  | 1kmdis10m |  |  |  | 102 | 26 | 25.49% | 102 | 26 | 25.49% |
|  | 30m | 5115.3462 | 1241.2341 | 24.26% | 3250.1637 | 1585.5975 | 48.79% | 4330.7181 | 2791.0773 | 64.45% |
|  | 30mdis10m |  |  |  | 3066.0048 | 1417.8843 | 46.25% | 3540.4704 | 2091.7845 | 59.08% |
| *Yushania ailuropodina* | 1km | 6358 | 907 | 14.27% | 13175 | 1547 | 11.74% | 12935 | 2291 | 17.71% |
|  | 1kmdis10m |  |  |  | 6001 | 850 | 14.16% | 5381 | 788 | 14.64% |
|  | 30m | 3745.3851 | 766.557 | 20.47% | 14481.0351 | 1475.3988 | 10.19% | 12888.6984 | 1613.5182 | 12.52% |
|  | 30mdis10m |  |  |  | 2940.885 | 652.0482 | 22.17% | 1985.7438 | 257.2362 | 12.95% |

Note: The definitions of abbreviations and indicators in this table are identical to those in Table S51.

Table 55 Statistics of total and high-altitude (3000–4500 m) distribution characteristics for 20 bamboo species under the SSP2-4.5 scenario based on the MRI-ESM2-0 model

| Species | Data type | Baseline area | Baseline area (3000–4500 m) | Baseline proportion (3000–4500 m) (%) | M245_mid area | M245_mid area (3000–4500 m) | M245_mid proportion (3000–4500 m) (%) | M245_end area | M245_end area (3000–4500 m) | M245_end proportion (3000–4500 m) (%) |
| --- | --- | --- | --- | --- | --- | --- | --- | --- | --- | --- |
| *Chimonobambusa szechuanensis* | 1km | 8661 | 3 | 0.03% | 4613 | 8 | 0.17% | 4442 | 21 | 0.47% |
|  | 1kmdis10m |  |  |  | 3263 | 1 | 0.03% | 2597 | 1 | 0.04% |
|  | 30m | 8183.3202 | 2.0007 | 0.02% | 2360.4786 | 233.7192 | 9.90% | 4309.4268 | 1183.0851 | 27.45% |
|  | 30mdis10m |  |  |  | 1627.9803 | 29.8503 | 1.83% | 2203.3125 | 114.1821 | 5.18% |
| *Yushania glauca* | 1km | 2609 | 258 | 9.89% | 1145 | 447 | 39.04% | 2145 | 973 | 45.36% |
|  | 1kmdis10m |  |  |  | 842 | 217 | 25.77% | 755 | 210 | 27.81% |
|  | 30m | 2681.4483 | 263.8773 | 9.84% | 1745.514 | 1232.8461 | 70.63% | 1999.7289 | 1800.9117 | 90.06% |
|  | 30mdis10m |  |  |  | 783.6435 | 502.6041 | 64.14% | 322.5645 | 262.8351 | 81.48% |
| *Phyllostachys bissetii* | 1km | 7103 | 0 | 0.00% | 5339 | 8 | 0.15% | 5806 | 127 | 2.19% |
|  | 1kmdis10m |  |  |  | 3190 | 0 | 0.00% | 1867 | 0 | 0.00% |
|  | 30m | 6406.9146 | 0.4185 | 0.01% | 555.84 | 5.6052 | 1.01% | 1209.4074 | 186.3504 | 15.41% |
|  | 30mdis10m |  |  |  | 379.899 | 2.1519 | 0.57% | 168.7212 | 10.6947 | 6.34% |
| *Yushania maculata* | 1km | 5778 | 1772 | 30.67% | 4214 | 2508 | 59.52% | 5925 | 3871 | 65.33% |
|  | 1kmdis10m |  |  |  | 2972 | 1521 | 51.18% | 2427 | 1472 | 60.65% |
|  | 30m | 5406.8625 | 1681.2603 | 31.09% | 2434.8834 | 1775.1933 | 72.91% | 7443.9297 | 5071.077 | 68.12% |
|  | 30mdis10m |  |  |  | 1729.9431 | 1213.641 | 70.15% | 2585.0007 | 1915.1991 | 74.09% |
| *Fargesia scabrida* | 1km | 4876 | 173 | 3.55% | 8166 | 2117 | 25.92% | 4840 | 1738 | 35.91% |
|  | 1kmdis10m |  |  |  | 3559 | 145 | 4.07% | 1325 | 90 | 6.79% |
|  | 30m | 7459.812 | 793.4553 | 10.64% | 19912.1589 | 10345.9644 | 51.96% | 4040.559 | 3707.5509 | 91.76% |
|  | 30mdis10m |  |  |  | 10732.2741 | 3842.5383 | 35.80% | 1570.6269 | 1300.7421 | 82.82% |
| *Yushania dafengdingensis* | 1km | 5453 | 846 | 15.51% | 4508 | 775 | 17.19% | 525 | 100 | 19.05% |
|  | 1kmdis10m |  |  |  | 4029 | 719 | 17.85% | 525 | 100 | 19.05% |
|  | 30m | 1624.689 | 277.8129 | 17.10% | 3608.4906 | 1435.4181 | 39.78% | 1775.4786 | 1353.2643 | 76.22% |
|  | 30mdis10m |  |  |  | 2651.3271 | 916.6212 | 34.57% | 346.0176 | 202.9689 | 58.66% |
| *Yushania brevipaniculata* | 1km | 11495 | 635 | 5.52% | 10065 | 2459 | 24.43% | 4405 | 1059 | 24.04% |
|  | 1kmdis10m |  |  |  | 6628 | 510 | 7.69% | 2904 | 246 | 8.47% |
|  | 30m | 13616.19 | 768.9438 | 5.65% | 11662.0299 | 5704.002 | 48.91% | 3768.5907 | 1569.2706 | 41.64% |
|  | 30mdis10m |  |  |  | 7030.8747 | 2281.2606 | 32.45% | 2540.2122 | 600.1155 | 23.62% |
| *Arundinaria spanostachya* | 1km | 2083 | 1393 | 66.87% | 1146 | 898 | 78.36% | 992 | 757 | 76.31% |
|  | 1kmdis10m |  |  |  | 928 | 714 | 76.94% | 315 | 268 | 85.08% |
|  | 30m | 1363.7655 | 1028.7909 | 75.44% | 29.8422 | 21.1203 | 70.77% | 266.9832 | 228.6036 | 85.62% |
|  | 30mdis10m |  |  |  | 24.7581 | 19.7523 | 79.78% | 3.0456 | 2.7756 | 91.13% |
| *Fargesia ferax* | 1km | 9092 | 1128 | 12.41% | 9921 | 2371 | 23.90% | 5272 | 1388 | 26.33% |
|  | 1kmdis10m |  |  |  | 7554 | 1085 | 14.36% | 4297 | 785 | 18.27% |
|  | 30m | 5022.6597 | 447.1425 | 8.90% | 6392.6928 | 2253.5163 | 35.25% | 315.5436 | 99.5103 | 31.54% |
|  | 30mdis10m |  |  |  | 4845.3822 | 1182.2958 | 24.40% | 283.3425 | 69.2991 | 24.46% |
| *Fargesia robusta* | 1km | 6583 | 267 | 4.06% | 7266 | 1941 | 26.71% | 4221 | 1618 | 38.33% |
|  | 1kmdis10m |  |  |  | 3835 | 186 | 4.85% | 1406 | 101 | 7.18% |
|  | 30m | 5650.8849 | 289.782 | 5.13% | 3495.3804 | 1609.8471 | 46.06% | 3459.1086 | 2781.3645 | 80.41% |
|  | 30mdis10m |  |  |  | 2629.4643 | 895.1904 | 34.04% | 2191.4658 | 1615.2129 | 73.70% |
| *Fargesia nitida* | 1km | 17563 | 6627 | 37.73% | 20914 | 14477 | 69.22% | 20804 | 15477 | 74.39% |
|  | 1kmdis10m |  |  |  | 12377 | 6329 | 51.14% | 10064 | 5684 | 56.48% |
|  | 30m | 17192.2572 | 6639.8868 | 38.62% | 23261.2029 | 19994.1039 | 85.95% | 12380.2065 | 9372.2247 | 75.70% |
|  | 30mdis10m |  |  |  | 15187.5765 | 12515.9355 | 82.41% | 8334.8946 | 6913.1673 | 82.94% |
| *Arundinaria faberi* | 1km | 18063 | 4875 | 26.99% | 14655 | 6862 | 46.82% | 5253 | 2731 | 51.99% |
|  | 1kmdis10m |  |  |  | 11634 | 4334 | 37.25% | 4264 | 1939 | 45.47% |
|  | 30m | 15221.7054 | 4219.3269 | 27.72% | 14491.4319 | 10080.153 | 69.56% | 5979.672 | 2793.0438 | 46.71% |
|  | 30mdis10m |  |  |  | 10937.1474 | 7229.8557 | 66.10% | 5414.7636 | 2433.2211 | 44.94% |
| *Yushania mabianensis* | 1km | 8325 | 915 | 10.99% | 6974 | 1383 | 19.83% | 4769 | 1210 | 25.37% |
|  | 1kmdis10m |  |  |  | 5496 | 777 | 14.14% | 3143 | 592 | 18.84% |
|  | 30m | 2765.8845 | 144.6372 | 5.23% | 1326.2382 | 773.253 | 58.30% | 2.5605 | 1.0521 | 41.09% |
|  | 30mdis10m |  |  |  | 738.8892 | 293.0697 | 39.66% | 2.3553 | 0.8469 | 35.96% |
| *Fargesia rufa* | 1km | 4129 | 51 | 1.24% | 8377 | 1049 | 12.52% | 8380 | 1275 | 15.21% |
|  | 1kmdis10m |  |  |  | 3362 | 48 | 1.43% | 2451 | 42 | 1.71% |
|  | 30m | 5903.9262 | 324.5157 | 5.50% | 16155.8307 | 10047.4011 | 62.19% | 15494.0922 | 11791.5408 | 76.10% |
|  | 30mdis10m |  |  |  | 8113.4586 | 3298.2102 | 40.65% | 5031.5661 | 3858.129 | 76.68% |
| *Chimonobambusa tumidissinoda* | 1km | 1975 | 37 | 1.87% | 1871 | 25 | 1.34% | 718 | 131 | 18.25% |
|  | 1kmdis10m |  |  |  | 1038 | 7 | 0.67% | 278 | 4 | 1.44% |
|  | 30m | 2016.4428 | 73.5921 | 3.65% | 1617.5988 | 146.9961 | 9.09% | 2602.899 | 2381.0418 | 91.48% |
|  | 30mdis10m |  |  |  | 1316.8296 | 63.2556 | 4.80% | 30.3201 | 19.107 | 63.02% |
| *Fargesia denudate* | 1km | 9463 | 2636 | 27.86% | 19111 | 9595 | 50.21% | 20058 | 10483 | 52.26% |
|  | 1kmdis10m |  |  |  | 9281 | 2612 | 28.14% | 8692 | 2507 | 28.84% |
|  | 30m | 10640.826 | 3289.6809 | 30.92% | 28300.545 | 17756.0055 | 62.74% | 7441.0695 | 3380.5287 | 45.43% |
|  | 30mdis10m |  |  |  | 22038.8157 | 12357.4212 | 56.07% | 5895.7497 | 3272.9265 | 55.51% |
| *Chimonobambusa opienensis* | 1km | 3087 | 10 | 0.32% | 1110 | 16 | 1.44% | 2942 | 191 | 6.49% |
|  | 1kmdis10m |  |  |  | 941 | 6 | 0.64% | 759 | 5 | 0.66% |
|  | 30m | 3567.3525 | 114.5889 | 3.21% | 1537.3971 | 408.6135 | 26.58% | 16443.9144 | 4340.8683 | 26.40% |
|  | 30mdis10m |  |  |  | 1048.8546 | 247.0932 | 23.56% | 2878.3467 | 663.4053 | 23.05% |
| *Yushania lineolata* | 1km | 12052 | 2563 | 21.27% | 8222 | 3530 | 42.93% | 7810 | 3486 | 44.64% |
|  | 1kmdis10m |  |  |  | 6611 | 2206 | 33.37% | 3836 | 1697 | 44.24% |
|  | 30m | 12282.5601 | 2430.8352 | 19.79% | 12622.536 | 4787.8587 | 37.93% | 5008.4478 | 1494.0288 | 29.83% |
|  | 30mdis10m |  |  |  | 11401.2558 | 3854.4849 | 33.81% | 4743.2277 | 1254.3102 | 26.44% |
| *Fargesia obliqua* | 1km | 6045 | 1403 | 23.21% | 6755 | 3269 | 48.39% | 4129 | 1630 | 39.48% |
|  | 1kmdis10m |  |  |  | 4251 | 1261 | 29.66% | 2869 | 869 | 30.29% |
|  | 30m | 5115.3462 | 1241.2341 | 24.26% | 19116.9306 | 13374.3438 | 69.96% | 4909.3731 | 3132.1089 | 63.80% |
|  | 30mdis10m |  |  |  | 15108.8202 | 9489.0231 | 62.80% | 4350.3876 | 2586.9186 | 59.46% |
| *Yushania ailuropodina* | 1km | 6358 | 907 | 14.27% | 4593 | 1265 | 27.54% | 6225 | 2027 | 32.56% |
|  | 1kmdis10m |  |  |  | 3946 | 840 | 21.29% | 3199 | 808 | 25.26% |
|  | 30m | 3745.3851 | 766.557 | 20.47% | 1978.0452 | 1089.4959 | 55.08% | 5592.8169 | 4015.9152 | 71.80% |
|  | 30mdis10m |  |  |  | 1616.2506 | 870.6708 | 53.87% | 2042.6508 | 1124.4672 | 55.05% |

Note: The definitions of abbreviations and indicators in this table are identical to those in Table S51.

Table 56 Statistics of total and high-altitude (3000–4500 m) distribution characteristics for 20 bamboo species under the SSP5-8.5 scenario based on the MRI-ESM2-0 model

| Species | Data type | Baseline area | Baseline area (3000–4500 m) | Baseline proportion (3000–4500 m) (%) | M585_mid area | M585_mid area (3000–4500 m) | M585_mid proportion (3000–4500 m) (%) | M585_end area | M585_end area (3000–4500 m) | M585_end proportion (3000–4500 m) (%) |
| --- | --- | --- | --- | --- | --- | --- | --- | --- | --- | --- |
| *Chimonobambusa szechuanensis* | 1km | 8661 | 3 | 0.03% | 3805 | 4 | 0.11% | 2307 | 122 | 5.29% |
|  | 1kmdis10m |  |  |  | 2906 | 1 | 0.03% | 886 | 1 | 0.11% |
|  | 30m | 8183.3202 | 2.0007 | 0.02% | 919.0917 | 66.0681 | 7.19% | 589.1823 | 512.1819 | 86.93% |
|  | 30mdis10m |  |  |  | 776.6451 | 17.6274 | 2.27% | 20.8512 | 2.4156 | 11.58% |
| *Yushania glauca* | 1km | 2609 | 258 | 9.89% | 1519 | 590 | 38.84% | 1110 | 757 | 68.20% |
|  | 1kmdis10m |  |  |  | 1077 | 234 | 21.73% | 427 | 186 | 43.56% |
|  | 30m | 2681.4483 | 263.8773 | 9.84% | 1177.1487 | 785.8161 | 66.76% | 2913.0336 | 1024.3872 | 35.17% |
|  | 30mdis10m |  |  |  | 757.7604 | 393.9165 | 51.98% | 226.0665 | 149.1003 | 65.95% |
| *Phyllostachys bissetii* | 1km | 7103 | 0 | 0.00% | 4203 | 7 | 0.17% | 1561 | 141 | 9.03% |
|  | 1kmdis10m |  |  |  | 2147 | 0 | 0.00% | 192 | 0 | 0.00% |
|  | 30m | 6406.9146 | 0.4185 | 0.01% | 351.162 | 12.2382 | 3.49% | 1014.7023 | 954.5328 | 94.07% |
|  | 30mdis10m |  |  |  | 166.4298 | 1.3905 | 0.84% | 25.4691 | 10.1151 | 39.72% |
| *Yushania maculata* | 1km | 5778 | 1772 | 30.67% | 3742 | 2387 | 63.79% | 3419 | 3005 | 87.89% |
|  | 1kmdis10m |  |  |  | 2649 | 1493 | 56.36% | 904 | 797 | 88.16% |
|  | 30m | 5406.8625 | 1681.2603 | 31.09% | 1896.6123 | 1437.7788 | 75.81% | 34.3827 | 3.1626 | 9.20% |
|  | 30mdis10m |  |  |  | 1310.7735 | 980.6895 | 74.82% | 6.147 | 1.998 | 32.50% |
| *Fargesia scabrida* | 1km | 4876 | 173 | 3.55% | 5496 | 1884 | 34.28% | 6145 | 5292 | 86.12% |
|  | 1kmdis10m |  |  |  | 1814 | 105 | 5.79% | 329 | 88 | 26.75% |
|  | 30m | 7459.812 | 793.4553 | 10.64% | 10482.8526 | 9058.3047 | 86.41% | 2414.6514 | 921.7755 | 38.17% |
|  | 30mdis10m |  |  |  | 3733.3926 | 2854.9125 | 76.47% | 432.0378 | 141.6906 | 32.80% |
| *Yushania dafengdingensis* | 1km | 5453 | 846 | 15.51% | 1528 | 242 | 15.84% | 5649 | 1299 | 23.00% |
|  | 1kmdis10m |  |  |  | 1495 | 238 | 15.92% | 1405 | 236 | 16.80% |
|  | 30m | 1624.689 | 277.8129 | 17.10% | 1151.6949 | 295.7958 | 25.68% | 7356.3174 | 2367.2682 | 32.18% |
|  | 30mdis10m |  |  |  | 788.6916 | 152.6715 | 19.36% | 1044.1656 | 253.5588 | 24.28% |
| *Yushania brevipaniculata* | 1km | 11495 | 635 | 5.52% | 7267 | 1389 | 19.11% | 10306 | 4010 | 38.91% |
|  | 1kmdis10m |  |  |  | 5604 | 341 | 6.08% | 3499 | 307 | 8.77% |
|  | 30m | 13616.19 | 768.9438 | 5.65% | 7494.1362 | 3023.2278 | 40.34% | 17291.277 | 7634.1672 | 44.15% |
|  | 30mdis10m |  |  |  | 5486.3865 | 1291.7862 | 23.55% | 4022.9145 | 877.5207 | 21.81% |
| *Arundinaria spanostachya* | 1km | 2083 | 1393 | 66.87% | 968 | 748 | 77.27% | 331 | 289 | 87.31% |
|  | 1kmdis10m |  |  |  | 704 | 548 | 77.84% | 109 | 101 | 92.66% |
|  | 30m | 1363.7655 | 1028.7909 | 75.44% | 144.4374 | 57.3597 | 39.71% | 1.2672 | 0.9738 | 76.85% |
|  | 30mdis10m |  |  |  | 105.7005 | 47.9304 | 45.35% | #VALUE! | #VALUE! | NA |
| *Fargesia ferax* | 1km | 9092 | 1128 | 12.41% | 8412 | 2231 | 26.52% | 9829 | 4431 | 45.08% |
|  | 1kmdis10m |  |  |  | 6479 | 1014 | 15.65% | 4517 | 975 | 21.59% |
|  | 30m | 5022.6597 | 447.1425 | 8.90% | 4089.9087 | 2261.5956 | 55.30% | 1402.0596 | 512.9262 | 36.58% |
|  | 30mdis10m |  |  |  | 2756.9124 | 1130.4234 | 41.00% | 918.2637 | 241.0299 | 26.25% |
| *Fargesia robusta* | 1km | 6583 | 267 | 4.06% | 5494 | 1714 | 31.20% | 6692 | 2614 | 39.06% |
|  | 1kmdis10m |  |  |  | 2161 | 151 | 6.99% | 417 | 66 | 15.83% |
|  | 30m | 5650.8849 | 289.782 | 5.13% | 2469.5244 | 335.5461 | 13.59% | 10944.1962 | 2766.3489 | 25.28% |
|  | 30mdis10m |  |  |  | 1751.9949 | 142.0812 | 8.11% | 1502.9424 | 318.2409 | 21.17% |
| *Fargesia nitida* | 1km | 17563 | 6627 | 37.73% | 18930 | 13886 | 73.35% | 17994 | 15233 | 84.66% |
|  | 1kmdis10m |  |  |  | 10001 | 5645 | 56.44% | 5725 | 4314 | 75.35% |
|  | 30m | 17192.2572 | 6639.8868 | 38.62% | 12396.1725 | 9754.3998 | 78.69% | 6493.5288 | 3032.9586 | 46.71% |
|  | 30mdis10m |  |  |  | 7794.3393 | 6223.4352 | 79.85% | 1998.0063 | 703.584 | 35.21% |
| *Arundinaria faberi* | 1km | 18063 | 4875 | 26.99% | 9408 | 4416 | 46.94% | 12814 | 8335 | 65.05% |
|  | 1kmdis10m |  |  |  | 8070 | 3188 | 39.50% | 4310 | 2372 | 55.03% |
|  | 30m | 15221.7054 | 4219.3269 | 27.72% | 12137.5224 | 6544.7091 | 53.92% | 14889.3777 | 7262.9793 | 48.78% |
|  | 30mdis10m |  |  |  | 8816.8176 | 4166.541 | 47.26% | 8248.4478 | 3591.1008 | 43.54% |
| *Yushania mabianensis* | 1km | 8325 | 915 | 10.99% | 4662 | 1129 | 24.22% | 7616 | 3096 | 40.65% |
|  | 1kmdis10m |  |  |  | 3649 | 625 | 17.13% | 2668 | 622 | 23.31% |
|  | 30m | 2765.8845 | 144.6372 | 5.23% | 424.7631 | 295.4574 | 69.56% | 4.4595 | 3.1194 | 69.95% |
|  | 30mdis10m |  |  |  | 313.875 | 188.4636 | 60.04% | 3.1707 | 2.3553 | 74.28% |
| *Fargesia rufa* | 1km | 4129 | 51 | 1.24% | 8399 | 893 | 10.63% | 11226 | 3448 | 30.71% |
|  | 1kmdis10m |  |  |  | 3122 | 47 | 1.51% | 2525 | 47 | 1.86% |
|  | 30m | 5903.9262 | 324.5157 | 5.50% | 17250.741 | 12688.0119 | 73.55% | 13700.6883 | 10813.8177 | 78.93% |
|  | 30mdis10m |  |  |  | 6154.1316 | 3804.2181 | 61.82% | 3901.6899 | 2898.1809 | 74.28% |
| *Chimonobambusa tumidissinoda* | 1km | 1975 | 37 | 1.87% | 1269 | 27 | 2.13% | 1141 | 152 | 13.32% |
|  | 1kmdis10m |  |  |  | 857 | 7 | 0.82% | 421 | 5 | 1.19% |
|  | 30m | 2016.4428 | 73.5921 | 3.65% | 1048.9284 | 273.6846 | 26.09% | 958.185 | 677.0358 | 70.66% |
|  | 30mdis10m |  |  |  | 693.8784 | 22.9923 | 3.31% | 83.6766 | 46.422 | 55.48% |
| *Fargesia denudate* | 1km | 9463 | 2636 | 27.86% | 19447 | 9193 | 47.27% | 20754 | 9978 | 48.08% |
|  | 1kmdis10m |  |  |  | 8886 | 2440 | 27.46% | 8542 | 2401 | 28.11% |
|  | 30m | 10640.826 | 3289.6809 | 30.92% | 19136.9655 | 10268.0775 | 53.66% | 12301.7076 | 4390.0605 | 35.69% |
|  | 30mdis10m |  |  |  | 14572.9566 | 8083.6686 | 55.47% | 5715.6345 | 2621.9655 | 45.87% |
| *Chimonobambusa opienensis* | 1km | 3087 | 10 | 0.32% | 1428 | 36 | 2.52% | 222 | 58 | 26.13% |
|  | 1kmdis10m |  |  |  | 1022 | 7 | 0.68% | 106 | 5 | 4.72% |
|  | 30m | 3567.3525 | 114.5889 | 3.21% | 2362.9419 | 267.4557 | 11.32% | 1319.823 | 165.8619 | 12.57% |
|  | 30mdis10m |  |  |  | 1550.1474 | 197.4744 | 12.74% | 373.2138 | 89.5698 | 24.00% |
| *Yushania lineolata* | 1km | 12052 | 2563 | 21.27% | 8043 | 3025 | 37.61% | 7049 | 5121 | 72.65% |
|  | 1kmdis10m |  |  |  | 6250 | 1921 | 30.74% | 2538 | 1336 | 52.64% |
|  | 30m | 12282.5601 | 2430.8352 | 19.79% | 8400.2409 | 2607.7275 | 31.04% | 8479.7028 | 2198.6703 | 25.93% |
|  | 30mdis10m |  |  |  | 7713.5616 | 2128.7304 | 27.60% | 7514.3682 | 1734.6411 | 23.08% |
| *Fargesia obliqua* | 1km | 6045 | 1403 | 23.21% | 7605  4395 | 3398 | 44.68% | 13168 | 6255 | 47.50% |
|  | 1kmdis10m |  |  |  |  | 1191 | 27.10% | 4294 | 1137 | 26.48% |
|  | 30m | 5115.3462 | 1241.2341 | 24.26% | 21518.0307 | 14536.8567 | 67.56% | 41479.5816 | 28153.9962 | 67.87% |
|  | 30mdis10m |  |  |  | 16595.3619 | 9880.6005 | 59.54% | 29265.5682 | 18768.8619 | 64.13% |
| *Yushania ailuropodina* | 1km | 6358 | 907 | 14.27% | 3852 | 886 | 23.00% | 4081 | 1984 | 48.62% |
|  | 1kmdis10m |  |  |  | 3621 | 761 | 21.02% | 2018 | 667 | 33.05% |
|  | 30m | 3745.3851 | 766.557 | 20.47% | 1319.6385 | 754.3413 | 57.16% | 88.3404 | 36.7083 | 41.55% |
|  | 30mdis10m |  |  |  | 947.9655 | 518.3109 | 54.68% | 16.5456 | 2.3742 | 14.35% |

Note: The definitions of abbreviations and indicators in this table are identical to those in Table S51.
